# Trial-level characteristics associate with treatment effect estimates: a systematic review of meta-epidemiological studies

**DOI:** 10.1186/s12874-022-01650-5

**Published:** 2022-06-15

**Authors:** Huan Wang, Jinlu Song, Yali Lin, Wenjie Dai, Yinyan Gao, Lang Qin, Yancong Chen, Wilson Tam, Irene XY Wu, Vincent CH Chung

**Affiliations:** 1 5/F, Xiangya School of Public Health, No. 238, Shang-ma-yuan-ling Alley, Kaifu district, Changsha, China; 2 Alice Lee Centre for Nursing Studies, National University of Singapore, Singapore, Singapore; 3 Hunan Provincial Key Laboratory of Clinical Epidemiology, Changsha, Hunan China; 4 Jockey Club School of Public Health and Primary Care, The Chinese University of Hong Kong, Hong Kong, China; 5 School of Chinese Medicine, The Chinese University of Hong Kong, Hong Kong, China

**Keywords:** Meta-epidemiological study, Randomized controlled trial, Systematic review, Treatment effect estimates, Trial-level characteristic

## Abstract

**Background:**

To summarize the up-to-date empirical evidence on trial-level characteristics of randomized controlled trials associated with treatment effect estimates.

**Methods:**

A systematic review searched three databases up to August 2020. Meta-epidemiological (ME) studies of randomized controlled trials on intervention effect were eligible. We assessed the methodological quality of ME studies using a self-developed criterion. Associations between treatment effect estimates and trial-level characteristics were presented using forest plots.

**Results:**

Eighty ME studies were included, with 25/80 (31%) being published after 2015. Less than one-third ME studies critically appraised the included studies (26/80, 33%), published a protocol (23/80, 29%), and provided a list of excluded studies with justifications (12/80, 15%). Trials with high or unclear (versus low) risk of bias on sequence generation (3/14 for binary outcome and 1/6 for continuous outcome), allocation concealment (11/18 and 1/6), double blinding (5/15 and 2/4) and smaller sample size (4/5 and 2/2) significantly associated with larger treatment effect estimates. Associations between high or unclear risk of bias on allocation concealment (5/6 for binary outcome and 1/3 for continuous outcome), double blinding (4/5 and 1/3) and larger treatment effect estimates were more frequently observed for subjective outcomes. The associations between treatment effect estimates and non-blinding of outcome assessors were removed in trials using multiple observers to reach consensus for both binary and continuous outcomes. Some trial characteristics in the Cochrane risk-of-bias (RoB2) tool have not been covered by the included ME studies, including using validated method for outcome measures and selection of the reported results from multiple outcome measures or multiple analysis based on results (e.g., significance of the results).

**Conclusions:**

Consistently significant associations between larger treatment effect estimates and high or unclear risk of bias on sequence generation, allocation concealment, double blinding and smaller sample size were found. The impact of allocation concealment and double blinding were more consistent for subjective outcomes. The methodological and reporting quality of included ME studies were dissatisfactory. Future ME studies should follow the corresponding reporting guideline. Specific guidelines for conducting and critically appraising ME studies are needed.

**Supplementary Information:**

The online version contains supplementary material available at 10.1186/s12874-022-01650-5.

## Background

Randomized controlled trial (RCT) is regarded as the best reliable study design for evaluating the efficacy or effectiveness of healthcare interventions [1, 2]. The results of RCTs could be the cornerstone of supporting clinical practice and improving public health policy decision [1]. However, defects in the design, conduct, analysis, interpretation and report have a substantial impact on the internal validity of RCTs, further distort the results of systematic reviews based on them, and ultimately cause inappropriate clinical decisions [3–5]. For example, a large body of empirical evidence has indicated that high or unclear risk of bias on allocation concealment [6–8], lack of blinding [2, 8, 9], smaller sample size [4, 10, 11], and single center trial [5, 12] showed larger treatment effect estimates. Therefore, it is urgent to identify these factors that could contort treatment effect estimates so as to ensure the authenticity of conclusions drawn from RCTs by scientifically rigorous design and methodology [8].

Based on the results of meta-analyses, meta-epidemiological (ME) study is a method of exploring the influence of specific trial-level characteristic on treatment effect estimates [12]. The Cochrane risk-of-bias (RoB) tool, which is widely used for assessing the risk of bias of RCTs, was developed based on evidence generated from ME studies [13, 14]. Related systematic reviews of ME studies have been published in 2016 with literature search date up to May 2015 [15, 16]. However, an increasing number of ME studies have been published after May 2015, which have not been included in the previous systematic reviews [15, 16]. Some of those newly published ME studies showed inconsistent results on the associations between treatment effect estimates and trial-level characteristics, such as drop out [17, 18], Medline indexed [4, 19] and double blinding [described as double blinding or ≥ 2 key parties (participants, personnel, outcome assessors) were blinded] [8, 20], while other newly published ME studies explored additional trial-level characteristics, which have not been investigated by the previous ME studies, neither did they have been covered by the previous systematic reviews accordingly [15, 16] (e.g., trial protocol registration [3, 21] and patient − reported outcome measures) [22]. So it is necessary for us to update the evidence.

This systematic review aimed to 1) summarize the empirical evidence regarding ME studies that investigated the associations between trial-level characteristics of RCTs and treatment effect estimates; 2) inform future best practice in RCT design as well as to provide empirical evidence for updating critical appraisal tool (e.g., The Cochrane RoB tool) for RCT; 3) describe characteristics of ME studies and methods used for the critical appraisal of ME studies, which will serve as a foundation for further development.

## Methods

### Protocol and registration

We performed and reported this systematic review with reference to guidance from the Cochrane Handbook for Systematic Reviews of Interventions [13] and the Preferred Reporting Items for Systematic Reviews and Meta-Analyses (PRISMA) recommendations [23]. The protocol of this study was registered on the PROSPERO (CRD42020200947).

### Eligibility criteria

A ME study of RCTs, which assessed the efficacy, effectiveness or safety of an intervention was eligible, and the intervention can be therapeutic or preventive (e.g., vaccines). We only included ME study if it examined the differences in treatment effect estimates stratified by variation in trial-level characteristics (e.g., method of allocation concealment). There were no restrictions on language and publication date.

We excluded ME studies that compared treatment effect estimates between RCTs and observational studies. ME studies comparing treatment effect estimates according to different quantitative methodological quality scores of RCTs (e.g., Jadad scale, ranged from 0 to 5 scores) were excluded as such method has been abandoned [24]. Conference abstracts, protocols, animal experiments, commentary, editorial or statistical methodology papers, and ME studies based on a single meta-analysis were excluded, as well. The most up-to-date version was included if the same ME study was published in different journals or was updated, with the remaining versions being regarded as supplementary sources for data extraction and critical appraisal.

### Literature search

Related systematic reviews [15, 16] have been published in 2016, which have conducted comprehensive literature search and identified eligible ME studies published before 2015. By adopting the common practice of previous updated systematic reviews [25, 26], we referred to the search strategies of previous systematic review [15] and searched PubMed, Embase, and Web of science with "meta-epidemiology", "treatment effect" and related keywords from January 2015 to August 2020. Reference lists of previously published systematic reviews [15, 16] as well as the identified ME studies were screened for additional studies. Although basing on the literature search results from the previous systematic reviews [15, 16] is a post-hoc decision, we believe it is an optimal choice in terms of saving time, manpower and resources without much (if any) compromising of the comprehensiveness of literature identification. Detailed search strategies were shown in Additional file 1: Appendix 1.

### Study selection and data extraction

All the retrieved citations were screened firstly based on titles and abstracts, and full texts of the remaining potentially eligible literatures were further assessed. Bibliographical characteristics of all of the eligible ME studies, including both searched by ourselves and references from the previously published systematic reviews [15, 16], were extracted using a self-developed form based on the previous systematic review [15]. The data extraction form has been piloted and refined among a sample of five ME studies. The study selection and data extraction were conducted by two trained researchers (HW, JL, WJ, YY, LQ and YC) in duplication. Any disagreement was discussed for consensus or consulted a senior researcher (IXYW). The following information was extracted from each ME study (Additional file 2: Appendix 2):

General characteristics of ME studies: year of publication; type of publication (journal article; agency report); involvement of epidemiologists/statisticians (referred to the definition reported by Delgado-Rodriguez et al. [27]); funding sources (public; private); type of intervention (pharmacology; non-pharmacology); medical conditions classified with the International Classification of Diseases 11th version (ICD-11); trial-level characteristics evaluated: some trial-level characteristics that included in the Cochrane RoB tool (e.g., sequence generation and allocation concealment), and others like sample size (larger sample, smaller sample) and number of centers (multicenter, single-center). Besides the above-mentioned pre-specified characteristics, we also included additional trial-level characteristics as post-hoc ones [e.g., publication language (English language, language other than English) and study design (parallel group, cross-over)] for the purpose of comprehensiveness.; type of outcome measure (binary; continuous; time-to-event); data sources for ME (collected meta-analyses, or trials, or previous ME studies);

Characteristics of the collections of meta-analysis: data sources (Cochrane review; non-Cochrane review); type of meta-analysis (aggregated data; individual participant data; network meta-analysis); management of overlapping meta-analyses; minimum number of trials per meta-analysis; criteria of selecting one meta-analysis from systematic review including more than one meta-analysis; data extraction sources (individual trial and/or systematic review);

Characteristic of quantitative analyses: statistical methods; methods used to account for clustering of trials within meta-analyses and to adjust meta-confounders; information related to heterogeneity and whether reported the direction of interpreting the results (e.g., stated that ratio of odds ratio (ROR) < 1 showed larger treatment effect estimates for trials with smaller sample size, as compared with larger sample size).

### Methodological quality assessment

To the best of our knowledge, there was no published tool specifically for evaluating the methodological quality of ME study. Hence, we used a self-developed criterion consisting of 16 items based on the AMSTAR 2 (A MeaSurement Tool to Assess systematic Reviews-2) [28] and the criteria used in a related systematic review published by Dechartres and colleagues [15]. Inclusion of these 16 items was based on consensus among all co-authors, with five items derived from AMSTAR 2 [28] and the remaining 11 items from Dechartres and colleagues’ criteria (Additional file 3: Appendix 3) [15]. Pairs of trained researchers (HW, JL and YL) independently assessed the methodological quality of included ME studies [15, 16]. Discrepancies were resolved by discussion or consulting a senior researcher (IXYW) when they persisted.

### Data analysis

All the results were narratively summarized and presented. Frequency (%) with their corresponding 95% confidence interval (CI) was used to summarize binary outcome, while median and interquartile or range for continuous outcome. Differences in treatment effect estimates were measured with ratio of effect size (e.g., ROR) for binary outcome and differences in standardized mean difference (SMD) for continuous outcome. Differences in treatment effect estimates were re-calculated to ensure a ratio of effect size less than 1 or a difference in SMD less than 0 reveal larger treatment effect estimates for trials with high or unclear risk of bias, or for trials with the second element (e.g., larger sample versus smaller sample, smaller sample was regarded as the second element). Associations between treatment effect estimates and trial-level characteristics were presented with forest plots. Similar to the previous systematic review [15], we did not combine the results from different ME studies instead of presenting them by forest plots due to the potential overlaps among ME studies. Results of subgroup analyses based on trial-level characteristics (e.g., type of outcome) or meta-analysis-level characteristics (e.g., type of review) were presented when available. All data analyses were conducted using R 3.6.1 (http://www.R-project.org, the R Foundation for Statistical Computing, Vienna, Austria).

## Results

**Fig. 1:**
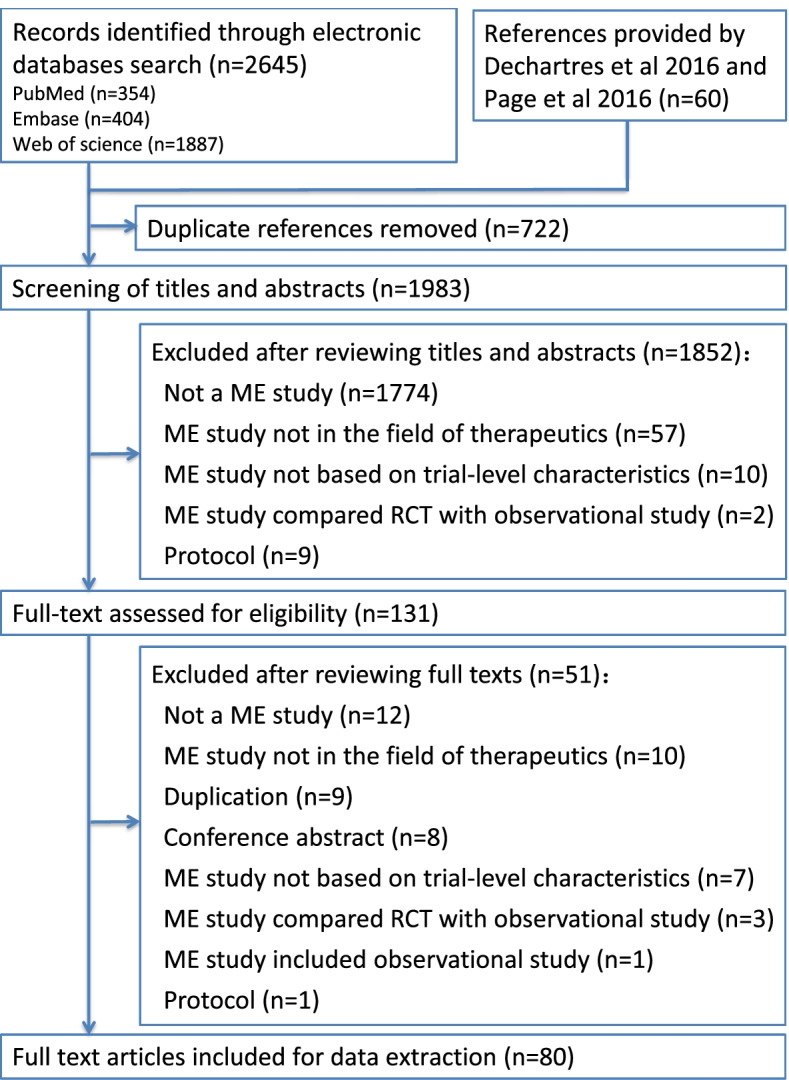
PRISMA flowchart: the literature search and selection of meta-epidemiological study on trial-level characteristics related to treatment effect estimates. ME, meta-epidemiological; RCT, randomized controlled trial

Overall, 2705 citations were identified based on electronic databases search and reference lists checking. After excluding duplications, the remaining 1983 records were screened by their titles and abstracts. Accordingly, 131 went through full text assessments, with 80 ME studies (Additional file 4: Appendix 4) being included, and the remaining 51 being excluded with reasons (Additional file 5: Appendix 5). Figure 1 describes the results of literature search and process of literature selection.

### Bibliographical characteristics

#### General characteristics

The 80 ME studies were published between 1995 and 2020 (median: 2013), with 25/80 (31%) being published after 2015 (the time of the last systematic review published) (Additional file 6: Appendix 6). Most ME studies were published as journal articles (76/80, 95%). Among them, 26/80 (32%) were published in general journals and 50/80 (62%) were published in medical specialty journals, including 26/80 (32%) in epidemiology/biostatistics journals. Moreover, 56/77 (73%) ME studies involved at least one epidemiologist/statistician. Among the 64 ME studies that provided funding information, only two (2/64, 3%) received funding from private sources, 48/64 (75%) from public sources while the remaining 14 (14/64, 22%) did not receive any funding support (Table 1).

**Table 1:** General characteristics of the 80 included meta-epidemiological (ME) studies^a^

**General characteristics**	**No. of ME study**^b^	**% (95% CI)**
**Median publication year, range**	2013	1995–2020
**Type of publication**		
Journal article	76	95 (90 to 100)
Agency report	4	5 (0 to 10)
**Journal articles**		
General journal	26	32 (22 to 43)
Medical specialty journal	50	62 (52 to 73)
Epidemiology/biostatistics	26	32 (22 to 43)
**Involvement of epidemiologists/statisticians**^c^	56/77	73 (63 to 83)
**Funding sources**^c^		
Public	48/64	75 (64 to 86)
Private	2/64	3 (0 to 8)
None	14/64	22 (12 to 32)
**Type of intervention**^c^		
Pharmacological intervention	5/62	8 (1 to 15)
Non-pharmacological intervention	6/62	10 (2 to 17)
Both	51/62	82 (72 to 92)
**Medical conditions**^c^		
Various medical areas	48/72	67 (56 to 78)
Diseases of the digestive system	9/72	12 (5 to 20)
Pregnancy, childbirth or the puerperium	5/72	7 (1 to 13)
Diseases of the musculoskeletal system or connective tissue	5/72	7 (1 to 13)
Others^e^	5/72	7 (1 to 13)
**Top five trial-level characteristics evaluated**		
Allocation concealment	30	38 (27 to 48)
Sequence generation	24	30 (20 to 40)
Double blinding^d^	19	24 (14 to 33)
Blinding of outcome assessors	18	22 (13 to 32)
Blinding of participants	13	16 (8 to 24)
**Type of outcome measure**^f^		
Binary	60	75 (65 to 85)
Continuous	37	46 (35 to 57)
Time-to-event	5	6 (1 to 12)
**Type of study design for literature search**		
Collection of meta-analyses	63	79 (70 to 88)
Collection of trials	11	14 (6 to 22)
Combination of previously published ME studies	6	8 (2 to 13)
**ME studies quantitatively synthesized a difference of treatment effect**	68	85 (77 to 93)

^a^Values are numbers of ME studies, percentage (95% confidence interval) unless stated otherwise. ^b^Denominators are 80 unless stated otherwise. ^c^Denominators are not equal to 80 as some ME studies did not report relate information

^d^Described as double-blinding or ≥ 2 key groups (participants, personnel, outcome assessors) were blinded. ^e^Others includes neoplasms (2, 2.8%), mental, behavioural or neurodevelopmental disorders (1, 1.4%), diseases of the nervous system (1, 1.4%), and symptoms, signs or clinical findings, not elsewhere classified (1, 1.4%). ^f^Total number was over 80, due to more than one type of outcome measure was used in some ME studies

Most (51/62, 82%) ME studies assessed both pharmacological and non-pharmacological interventions. Binary outcomes were included in 60/80 (75%) ME studies, while time-to-event outcomes were included in only 5/80 (6%). Two thirds (48/72, 67%) ME studies covered various medical areas, followed by diseases of the digestive system (9/72, 12%), pregnancy, childbirth, or the puerperium (5/72, 7%), and diseases of the musculoskeletal system or connective tissue (5/72, 7%) (Table 1, Additional file 7: Appendix 7). The most frequently evaluated trial-level characteristic was allocation concealment (30/80, 38%), followed with sequence generation (24/80, 30%), double blinding (19/80, 24%), blinding of outcome assessors (18/80, 22%), and blinding of participants (13/80, 16%). Additional file 8: Appendix 8 shows detailed trial-level characteristics evaluated in each ME study.

#### Details of the collected meta-analyses among the ME studies

Most (63/80, 79%) ME studies were based on data collected from meta-analyses, only 11/80 (14%) utilized data collected from trials and 6/80 (8%) directly collected data from previously published ME studies (Table 1). Among the 63 ME studies based on data from meta-analyses, 58 reported data sources, including 28/58 (48%) only considering Cochrane review, 3/58 (5%) only considering non-Cochrane review and 27/58 (47%) considering both. Most (58/63, 92%) ME studies were based on aggregated data meta-analyses, with the remaining five considered other type of meta-analyses, including both aggregated data and individual participant data (3/63, 5%), individual participant data only (1/63, 2%) and network of aggregated data only (1/63, 2%). Thirty-five (35/63, 56%) ME studies explicitly managed overlapping meta-analyses, whereas 28/63 (44%) did not report related information. The minimum number of trials included per meta-analysis ranged from one to ten, while 26/63 (41%) ME studies did not provide this information. When the included systematic review had more than one meta-analysis, forty-four (44/63, 70%) ME studies selected one meta-analysis from each systematic review, based on multiple criteria (20/44, 45%) or the primary outcome (10/44, 23%). Four ME studies (4/63, 6%) included all meta-analyses reported in systematic reviews without selection, while the remaining 15/63 (24%) did not mention relevant information (Table 2).

**Table 2:** Characteristics of 63 meta-epidemiological (ME) studies based on collection of meta-analyses^a^

**Characteristics**	**No. of ME study**^b^	**% (95% CI)**
**Data sources**^c^		
Cochrane review only	28/58	48 (35 to 62)
Non-Cochrane review only	3/58	5 (0 to 11)
Non-Cochrane review published in “high impact factor” journals	1/58	2 (0 to 5)
Both Cochrane and non-Cochrane review	27/58	47 (33 to 60)
Non-Cochrane review published in “high impact factor” journals and Cochrane	8/58	14 (5 to 23)
**Type of meta-analyses**		
Aggregated data only	58	92 (85 to 99)
Aggregated and individual participant data	3	5 (0 to 10)
Individual participant data only	1	2 (0 to 5)
Network of aggregated data only	1	2 (0 to 5)
**Management of overlapping meta-analyses**	35	56 (43 to 68)
**Reported minimum No. of trials included in each meta-analysis**	37	59 (46 to 71)
**Minimum No. of trials included in meta-analysis, median (Q1, Q3)**^**d**^**, range**^e^	3 (2,4)	1 to 10
**Criteria of selecting one meta-analysis within each systematic review**^f^		
Primary outcome	10/44	23 (10 to 36)
Largest number of studies	4/44	9 (0 to 18)
Objective	3/44	7 (0 to 15)
First outcome	2/44	4 (0 to 11)
Others^g^	5/44	11 (2 to 21)
More than one method	20/44	45 (30 to 61)
**Data extraction sources**^c^		
From individual trial	19/60	32 (20 to 44)
From meta-analysis	8/60	13 (4 to 22)
Both	33/60	55 (42 to 68)

^a^Values are numbers of ME studies, percentage (95% confidence interval) unless stated otherwise. ^b^Denominators are 63, unless stated otherwise. ^c^Denominators are not equal to 63 as some ME studies did not report relate information. ^d^Q1, Quartile 1. ^e^Among 63 ME studies, 26 ME studies did not report minimum number of trials included in meta-analysis. ^f^Denominator is 44, as four ME studies included all meta-analysis within each systematic review while 15 ME studies did not report related information. ^g^Others includes selection methods of first outcome statistically significant (1, 2.1%), mortality (1, 2.1%), most clinically relevant (1, 2.1%), most homogeneous (1, 2.1%) and at random (1, 2.1%)

#### Details of quantitative analyses among the ME studies

Most (68/80, 85%) ME studies quantitatively synthesized the difference of treatment effect estimates (Table 1). The most commonly used method for combining results was two-step approach (within-meta-analysis comparison and then combination) (43/68, 63%). Clustering of trials within a meta-analysis was accounted in 53 of the 61 (87%) ME studies based on data from meta-analyses. More than 70% ME studies assessed the heterogeneity during data synthesis (59/68, 87%), adjusted meta-confounders (54/68, 79%), and used random effect models to take into account variability across meta-analyses/trials (43/61, 70%). Sixty (60/68, 88%) ME studies clearly reported the direction of interpreting the results, while the remaining 8/68 (12%) did not provide this information. Forty-eight (48/68, 71%) ME studies conducted subgroup analyses either based on trial-level characteristics or meta-analysis-level characteristics (Table 3). Additional file 9: Appendix 9 presents detailed information on the subgroup analyses of the included ME studies.

**Table 3:** Characteristics of 68 meta-epidemiological (ME) studies quantitatively synthesized a difference of treatment effect estimates^a^

**Statistical analysis**	**No. of ME study**^b^	**% (95% CI)**
**Which model the ME study used to combine?**		
Two-step approach: within-meta-analysis comparison and combination	43	63 (52 to 75)
Logistic regression	7	10 (3 to 18)
Two-step approach: within-trial comparison and combination	5	7 (1 to 14)
Bayesian multilevel model	5	7 (1 to 14)
Meta-regression	3	4 (0 to 9)
More than one model^c^	5	7 (1 to 14)
**Accounted for clustering of trials within meta-analysis**^d^	53/61	87 (78 to 96)
**Used random effect models to account for variability across meta-analyses/trials**		
Yes^e^	43/61	70 (59 to 82)
No^e^	18/61	30 (18 to 41)
**Adjusted meta-confounders**	54	79 (70 to 89)
Based on subgroup analysis solely^e^	32/54	59 (46 to 73)
Based on multiple variable analysis solely^e^	6/54	11 (2 to 20)
Both^e^	16/54	30 (17 to 42)
If yes, as main analysis^e^	21/54	39 (26 to 52)
**Assessed the heterogeneity during analysis**	59	87 (78 to 95)
Based on qualitative domain solely (chi-square test)^e^	4/59	7 (0 to 13)
Based on quantitative domain solely (I^2^, τ^2^, φ^2^, F test)^e^	20/59	34 (22 to 46)
Both^e^	35/59	59 (46 to 72)
**Whether the author clearly reported the direction of interpretation of results**		
Yes	60	88 (80 to 96)
No	8	12 (4 to 20)
**Subgroup analyses performed**	48	71 (60 to 82)
Based on trial-level characteristics^e^	30/48	62 (48 to 77)
Based on meta-analysis-level characteristics^e^	1/48	2 (0 to 6)
Both^e^	17/48	35 (21 to 50)

^a^Values are numbers of ME studies, percentage (95% confidence interval) unless stated otherwise. ^b^Denominators are 68 unless stated otherwise. ^c^More than one model includes methods of using both two-step approach and multilevel model (3,4.9%), using both two-step approach and logistic regression (1,1.6%), and using both two-step approach, logistic regression and multilevel model (1,1.6%). ^d^Denominator is 61, as seven ME studies based on collection of trials are not applicable to this item. ^e^Denominator are not equal to 68, as some ME studies did not report related information

### Methodological quality

The included ME studies generally performed well in three items, with at least 90% compliance rates. These included giving a clear description of inclusion criteria and reasons for exclusion (74/80, 92%), reporting information related to conflicts of interest and funding supports (74/80, 92%), and providing a clear definition of trial characteristics evaluated in ME studies (72/80, 90%). On the other hand, less than one third ME studies fulfilled the following three methodological criteria: assessing the methodological quality of the included studies (26/80, 33%), publishing a protocol developed prior to the conduct of the ME study (23/80, 29%), and providing a list of excluded studies with justifications (12/80, 15%) (Table 4).

**Table 4:** Methodological quality of the sampled 80 meta-epidemiological (ME) studies

Methodological items	Yes		No	
	**No. of ME studies**	**% (95% CI)**	**No. of ME studies**	**% (95% CI)**
M1. Did the author state that they had published a protocol prior to the conduct of the ME study?	23	29 (19 to 39)	57	71 (61 to 81)
M2. Did the author use a comprehensive literature search strategy?	46	58 (46 to 69)	34	42 (31 to 54)
M3. Did the author give a clear description of inclusion criteria and reasons for exclusion?	74	92 (87 to 98)	6	8 (2 to 13)
M4. Whether selection process was reported?	69	86 (78 to 94)	11	14 (6 to 22)
M5. Did the author perform selection process in duplicate?	40	50 (39 to 61)	40	50 (39 to 61)
M6. Did the author perform data extraction in duplicate?^a^	52	88 (79 to 97)	7	12 (3 to 21)
M7. Did the author provide a list of excluded studies and justify the exclusions?^b^	12	15 (7 to 23)	65	81 (72 to 90)
M8. Did the author evaluate the heterogeneity between included meta-analyses or trials or ME studies?^c^	59	87 (78 to 95)	9	13 (5 to 22)
M8i. Did the author perform an investigation of sources of any heterogeneity?^c^	35	83 (72 to 95)	7	17 (5 to 28)
M9. Whether analysis was adjusted on meta-confounders for ME studies estimating a combined difference of treatment effect?^c^	54	79 (70 to 89)	14	21 (11 to 30)
M10. Whether clustering of trials within meta- analyses was taken into account for ME studies based on a collection of meta-analyses or previous ME studies?^c^	53	87 (78 to 96)	8	13 (4 to 22)
M11. Did the author report any potential sources of conflict of interest, including any funding they received for conducting the ME study?	74	92 (86 to 98)	6	8 (2 to 14)
M12. Whether checking experimental and control arms were reported?	40	51 (39 to 62)	40	49 (38 to 61)
M13. Whether the author reclassified of outcomes reported to have the same sense of interpretation?	56	71 (61 to 81)	24	29 (19 to 39)
M14. Did the author give a clear definition of trial characteristics evaluated in ME studies?	72	90 (83 to 97)	8	10 (3 to 17)
M15. Did the author assess the trial characteristics evaluated in duplicate?	64	80 (71 to 89)	16	20 (11 to 29)
M16. Did the author assess the methodological quality of the included ME studies or meta-analyses or trials?	26	33 (22 to 44)	54	67 (56 to 78)

^a^Denominator is 59 as 21 ME studies did not report related information. Among 52 ME studies perform data extraction in duplicate, 34 (57.6%) ME studies was fully or partly in duplicate, 6 (10.2%) was checked by a second reviewer, 3 (5.1%) mentioned contact to authors and 9 (15.3%) used more than one aforementioned method. ^b^Three ME studies provided a list of excluded studies but without reasons. ^c^Denominator is not equal to 80, as some ME studies were not applicable to this item

### Impact of trial-level characteristics on treatment effect estimates

#### Binary outcomes

**Fig. 2:**
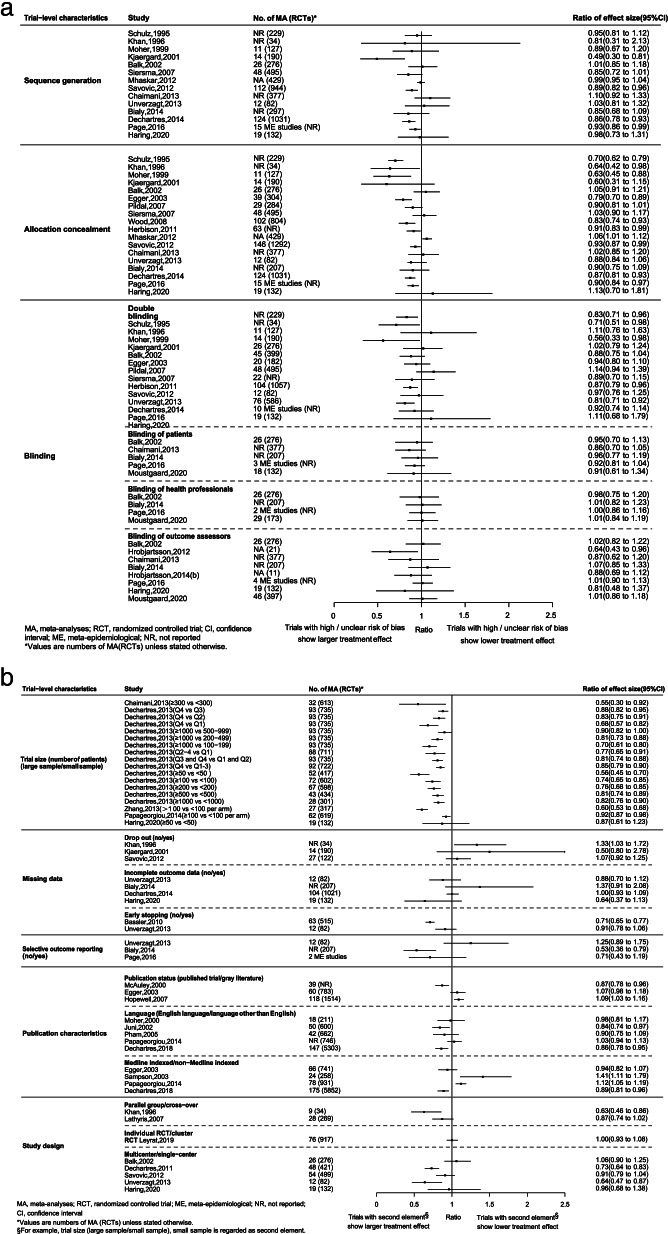
Associations between treatment effect estimates and trial-level characteristics for binary outcome

Eleven out of 14 (11/14) ME studies indicated that trials with high or unclear risk of bias for sequence generation showed associations with larger treatment effect estimates, three of which found such associations statistically significant. Fourteen out of 18 (14/18) ME studies showed trials with high of unclear risk of bias on allocation concealment were associated with larger treatment effect estimates (11 found statistically significant associations). Ten out of 15 (10/15) ME studies showed that trials with high or unclear risk of bias on double blinding related to larger treatment effect estimates, of which such associations in five ME studies were statistically significant. Aforementioned associations were also observed when blinding was considered separately as blinding of participants (5/5 ME studies), blinding of personnel (1/4 ME studies) and blinding of outcome assessors (4/8 ME studies). As for blinding of outcome assessor, one out of four ME studies showed statistically significant association) (Fig. 2).

All of (5/5) ME studies showed that trials with smaller sample size had an association with larger treatment effect estimates than that of trials with larger sample size, four of which found statistically significant associations. Above-mentioned significant association was especially seen in one ME study [11] regardless of the definition of smaller and larger sample size (e.g., Q1 versus Q4, < 50 versus ≥ 50) (Fig. 2, Additional file 10: Appendix 10). Two out of two (2/2) ME studies showed larger treatment effect estimates for early stopping trials, and such association was found statistically significant in 1/2 ME study. Inconsistencies in direction of point estimation on ratio of effect size were observed among the ME studies for trials with high or unclear risk of bias in incomplete outcome data (4 ME studies) and selective outcome reporting (3 ME studies). All of three (3/3) ME studies showed that published trials, compared with grey literature, produced larger treatment effect estimates, with 2/3 ME studies showing statistically significant association. Four out of five (4/5) ME studies showed larger treatment effect estimates for trials published in language other than English, two of which found it statistically significant. Inconsistent results were seen in non-Medline indexed trials versus Medline indexed trials as well, with two (2/4) ME studies showing lower treatment effect estimates for non-Medline indexed trials, while remained two (2/4) indicating larger.

Results from four out of five (4/5) ME studies revealed that single-center trials were associated with larger treatment effect estimates than that of multi-center trials, so did cross over trials than that of parallel trials (2/2 ME studies). Such associations were found statistically significant in 2/4 and 1/2 ME studies, respectively. Two out of four (2/4) ME studies found that trials without conducting intention to treat analysis showed larger treatment effect estimates, one of which found it statistically significant. Nonetheless, no statistical association were found between trials with baseline imbalance (3 ME studies), existence of competing interests (2 ME studies) and industry funding (3 ME studies) and treatment effect estimates (Fig. 2).

One ME study [29] demonstrated that overall trials showed significantly much lower treatment effect estimates than that of first trial (ratio of effect size: 2.67, 95% CI: 2.12–3.37), although the remaining ME study [30] did not find such association (ratio of effect size: 1.03, 95% CI: 0.98–1.08). Several other trial-level characteristics including sufficient follow-up, placebo control and statistician involvement, among others have been investigated as well, with no significant associations being found (Additional file 10: Appendix 10).

#### Continuous outcomes

**Fig. 3:**
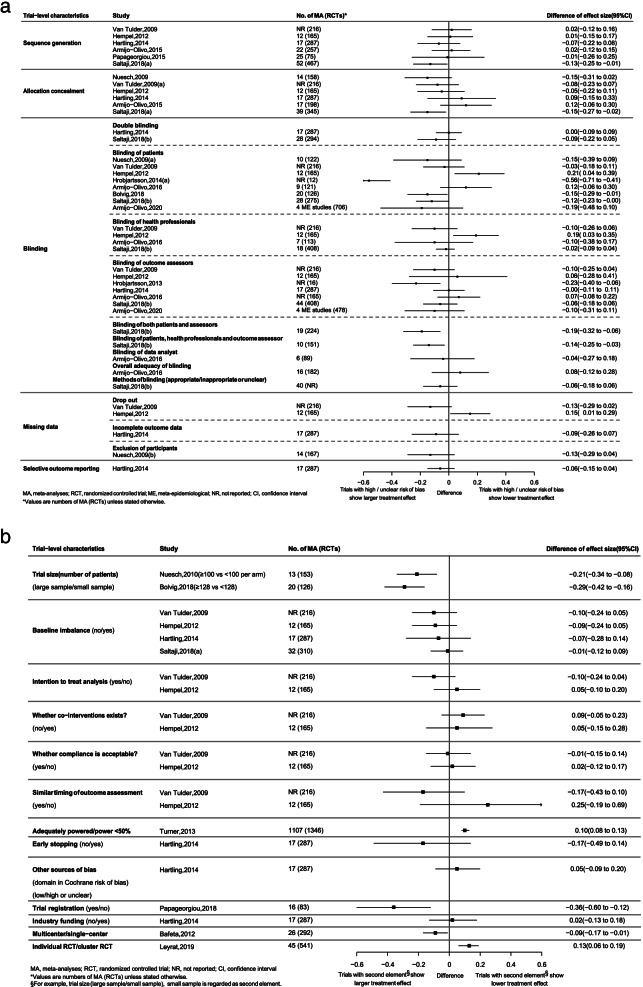
Associations between treatment effect estimates and trial-level characteristics for continuous outcome

Three out of six (3/6) ME studies reported the association between trials with high or unclear risk of bias on sequence generation and larger treatment effect estimates (1/3 ME study showing statistically significant association). Four out of six (4/6) ME studies showed trials with high or unclear risk of bias on allocation concealment related to larger treatment effect estimates, of which one ME study found it statistically significant. Inconsistencies in direction of point estimation on difference of effect size were seen among the ME studies when blinding was separately considered as three independent parties, including blinding of participants (8 ME studies), blinding of personnel (4 ME studies) and blinding of outcome assessors (7 ME studies). Such inconsistencies were removed when the three parties were considered at the same time as double blinding, with three out of four (3/4) ME studies showed larger treatment effect estimates for trials with high or unclear risk of bias (1/3 ME study found such association statistically significant) (Fig. 3).

Three ME studies consistently found that smaller sample size (or inadequate powered) trials were related to larger treatment effect estimates. One out of two (1/2) ME studies reported that trials with drop outs were associated with lower treatment effect estimates (Fig. 3), while the other ME study showed opposite direction. Additionally, single-center trials (1 ME study), individual RCT (versus cluster RCT) (1 ME study) and trials with no protocol registration (1 ME study) showed significant associations with larger treatment effect estimates. Most trial characteristics did not show any significant associations with treatment effect estimates in continuous outcomes, including early stopping (1 ME study), incomplete outcome reporting (1 ME study), selective outcome reporting (1 ME study), intention to treat analysis (2 ME studies), baseline imbalance (4 ME studies) and industry funded trials (1 ME study), among others (Fig. 3).

### Subgroup analyses

**Fig. 4:**
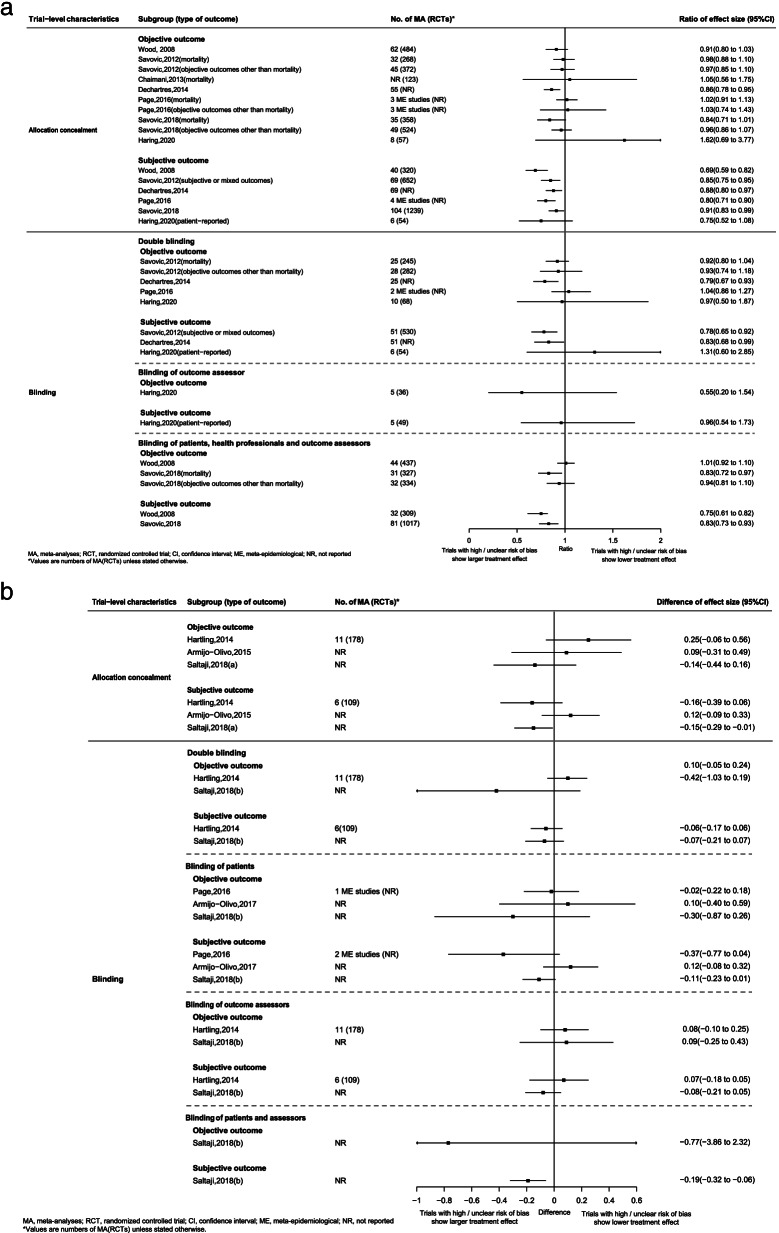
Associations between treatment effect estimates and trial-level characteristics based on type of outcome (objective and subjective outcome). **a** binary outcome; **b** continuous outcome

For binary outcomes, larger treatment effect estimates were observed in trials with high or unclear risk of bias on allocation concealment (6/6 ME studies for subjective outcome and 6/10 ME studies for objective outcome) and double blinding (4/5 ME studies for subjective outcome and 6/8 ME studies for objective outcome). The significant associations between high or unclear risk of bias and larger treatment effect estimates were much more frequently observed among subjective outcomes than that of objective outcomes [allocation concealment (5/6 versus 1/10 ME studies) and double blinding (4/5 versus 2/8 ME studies)] (Fig. 4-a). For continuous outcomes, trials with high or unclear risk of bias on allocation concealment (2/3 and 1/3 ME studies for subjective outcome and objective outcome, respectively) and double blinding (3/3 and 2/3 ME studies for subjective outcome and objective outcome, respectively) related to larger treatment effect estimates. However, 1/3 ME study found that above-mentioned associations were statistically significant only in the subjective outcome (Fig. 4-b).

For both binary and continuous outcomes, larger treatment effect estimates for trials with high or unclear risk of bias on blinding of outcome assessors were only observed in trials using single observer for non-blinded assessment (compared with trials using multiple observer consensus for non-blinded assessment) and trials with industry funding (trials with non-commercial funding) (Fig. 4).

For binary outcomes, larger treatment effect estimates for trials published in language other than English were only seen in trials with pharmacological intervention, using inactive control, focusing on complementary medicine and included in non-Cochrane review other than trials with non-pharmacological intervention, using active control, focusing on non-complementary medicine and included in Cochrane review (Additional file 11: Appendix 11-B-2, Appendix 11-B-3, Additional file 12: Appendix 12-B-1). For continuous outcomes, larger treatment effect estimates for trials with high or unclear risk of bias on blinding of participants were only demonstrated in non-pharmacological intervention trials (Additional file 11: Appendix 11-C-2), while the associations between treatment effect estimates and risk of bias for both blinding of participants and allocation concealment were only seen in complementary medicine trials (Additional file 11: Appendix 11-C-4). It is worth noted that larger treatment effect estimates in first trial as compared with subsequent trial were consistently observed regardless of the sample size (< 300 and > 300), risk of bias (low, unclear and high) or effect size (≤ 0.5 SMDs and > 0.5 SMDs) of the first trial for continuous outcomes (Additional file 12: Appendix 12-C-1). Such consistency has not been explored for binary outcomes. Details on subgroup analyses for both binary and continuous outcomes were displayed in Fig. 4, Additional file 11: Appendix 11 and Additional file 12: Appendix 12.

## Discussion

This systematic review identified 80 ME studies on intervention field, with almost one-third uncovered by the previous systematic reviews [15, 16]. The included ME studies covered various medical areas and interventions. An abundant of trial-level characteristics have been evaluated, varied from risk of bias domains (e.g., blinding) to language (English and non-English), and age of participants (e.g., children and adult), with allocation concealment, sequence generation and blinding being most commonly evaluated. On average, consistently significant associations with larger treatment effect estimates were observed in trials with high or unclear (versus low) risk of bias on sequence generation, allocation concealment, double blinding and smaller sample size. For allocation concealment and double blinding, the significant associations were more frequently observed in subjective outcomes. The impacts of missing outcome data and intention-to-treat included in the Cochrane RoB2 tool were uncertain. Furthermore, some characteristics in the Cochrane RoB2 tool have not been covered by the included ME studies yet, including using a validated method for outcome measures and selection of the reported results from multiple outcome measures or multiple analysis based on results (e.g., significance of the results).

Besides larger number and more updated ME studies were included when compared to the previous systematic reviews [15, 16], we identified some interesting findings in the subgroup analyses: i) High or unclear risk of bias on blinding of outcome assessors were significantly associated with larger treatment effect estimates in trials using single observer for non-blinded assessment for both binary and continuous outcomes. This finding indicates that when blinding of outcome assessor is not possible, reaching consensus by multiple assessors might be an alternative strategy to reduce potential detection bias; ii) larger treatment effect estimates for trials published in non-English (binary outcome), trials with high or unclear risk of bias on blinding of participants (continuous outcome) and allocation concealment (continuous outcome) were only seen in trials focusing on complementary medicine. A tentative explanation for the differences between these subgroups is that trials on complementary medicine had a higher probability of suffering from methodological flaws [31]; iii) larger treatment effect estimates in first trial as compared with subsequent trial were consistently observed, regardless of the trial size, risk of bias or effect size of the first trial for continuous outcomes, indicating the robustness of the association. However, such explorations are missing in binary outcomes, although inconsistencies were observed between the two available ME studies [29, 30]. That invites future ME studies to address.

Several reporting and methodological flaws among the sampled ME studies are worth to be noted. Over one-fifth ME studies missed reporting some key information such as funding sources, criteria used for selecting one meta-analysis within each systematic review and management of overlapping meta-analyses. Future ME studies are suggested to follow the corresponding reporting guideline [32] to improve their reporting and transparency. Commonly methodological flaws waiting for future ME studies to overcome included assessing the methodological quality of included studies, publishing a protocol, and providing a list of excluded studies with reasons. Furthermore, before the availability of a guideline for conducting ME studies, future ME studies could at least refer to existing publications regarding the statistical methods [33–35] and sample size [36] of a ME study.

Several additional key points regarding the conducting of ME studies worth discussed as well. Some preliminary steps are needed to reduce potential bias [37, 38] before combing differences in treatment effect estimates across meta-analyses or trials in a ME study. First, with regards to management of overlapping, using a study more than one time in the same quantitative analysis may overstate its sample size and number of events. Although it may produce greater precision and better robustness of the conclusions, the conclusion would be wrong [39]. However, almost half of the ME studies did not report whether overlapping meta-analyses were managed, which calls attentions from the future ME studies. Second, ensure the results from different meta-analyses have the same sense of interpretation [15] by checking experimental and control arm in each trial when two active interventions are compared [38], and reclassifying outcomes (e.g., survival re-coded as mortality) if needed [15]. However, only half of the ME studies reported information on whether experimental and control arm had been checked.

While using data from meta-analyses to assess the difference in treatment effect estimates, the results might be distorted by the presence of within- and between-meta-analysis heterogeneity if the clustering of trials within meta-analysis is not accounted for [40]. That was observed in more than 10% related ME studies. Being observational studies in nature, ME studies are generally at risk of confounding [38]. Despite repeated emphases [16, 33, 41], ME studies that completely controlled confounders are rare [42]. About four-fifth of the included ME studies adjusted meta-confounders, which have been improved compared to the previous systematic reviews [15, 16]. However, 59% adjusted confounders solely based on subgroup analysis, with very limited number of confounders being controlled at one time, indicating incomplete control of confounding. Alternatively, multiple variable analysis could be a better choice. Meanwhile, the selection of potential confounders is challenging, besides empirical evidence and theoretical consideration, the directed acyclic graph (DAG) approach proposed by Herbert [37] is recommended. Additionally, ME studies based on collection of trials could also reduce confounding through comparison within the same trial (e.g., compare blinding with non-blinding assessment) [15].

Further issues regarding confounding are that the association between blinding and treatment effect estimates were more consistent when more than one party (participants and assessors with/without personnel) was considered simultaneously as double blinding for both binary and continuous outcomes. During trial reporting, the CONSORT statement [43] encourages trial author to clearly state who is blinded rather than ambiguously state double blinding. However, in ME studies, blinding of different parties was generally correlated with each other (e.g., blinding of participants and blinding of personnel), accordingly, analyzing these parties separately without controlling the remaining ones might introduce confounding bias. Therefore, combining the three key parties (participants, personnel and outcome assessors) as one group might be an optimal choice for reducing confounding bias in ME studies. Similar consideration is needed for allocation concealment. We agree with Moustgaard et. al [1] that theoretically, the association between allocation concealment and treatment effect estimates should not depend on type of outcome (subjective or objective), which disagreed with available ME studies [16]. In theory, the confounding of blinding could be a major concern under such scenario. It is difficult to implement blinding especially blinding of participants and personnel when allocation sequence is unconcealed. Careful considerations of other confounders as well as the relationship among different trial characteristics are needed for future ME studies.

As agreeing with the previous systematic reviews [15, 16], this review also found that significant associations between trial-level characteristics and treatment effect estimates were much frequently seen in binary outcomes than that of continuous outcomes, including subgroup analyses. Larger sample of meta-analyses with more homogeneous data on binary outcomes [44] might contribute to the differences [15]. Although it have been raised by the previous systematic review [15], more attentions are still needed on continuous outcome for the future ME studies as results based on binary outcome may not be directly generalized to continuous outcome.

### Strengths and limitations

This systematic review has several strengths. First, no limit on medical areas and type of interventions ensured the generalizability of our results. Second, methodological quality of included ME studies has been assessed to inform where improvements are needed in the future. Third, comprehensive information related to subgroup analyses was extracted, and interesting subgroups like bias introduced by lack of blinding of outcome assessors might be removed by adopting multiple observer consensus [2] have been identified.

Some apparent limitations are worth noted in our study. First, some ME studies sometimes use “methodological study” or “research on research” to describe [45]. However, we directly adopted the literature search strategies from the previously published systematic review [15] to identify eligible ME studies. That did not include the aforementioned search terms, which probably led to missing some potentially eligible studies.

Second, there was no specific tool for assessing the methodological quality of ME studies. Therefore, we used a self-developed criterion through discussing within group members, without consulting external specialists.

Third, we extracted the results of unadjusted analysis for each ME study as nearly three-fifth ME studies adjusted confounders using subgroup analysis rather than multiple variables analysis (32/54, 59%) or did not report adjusted results (13/22, 59%).

Fourth, we did not combine the results quantitatively either for the main analyses or subgroup analyses due to the potential overlapping of meta-analyses and trials. Although we presented the results by considering both the statistically significant differences and the direction of treatment effect estimates to reduce the impact of solely based on vote counting. Without quantitative combination, the potential influence of Simpson’s paradox might not be completely removed. Furthermore, while conducting an ME study, duplications should be considered and removed [39]. However, among the 63 included ME studies based on collection of meta-analyses, only 35 (56%) managed the overlaps of RCTs. That calls for future ME studies to pay attention to the duplicated RCTs, especially when quantitative synthesis is conducted.

Fifth, only ME studies on intervention field were considered. Results from this review may not be generalized to other fields of ME studies, such as diagnosis accuracy [46–48], prognostic study [49, 50], and prediction models [51].

Sixth, related information of methodology and reporting was extracted based on publications, which may introduce bias if authors did not conduct as reported or did not report related information.

### Implications

Identifying trial-level characteristics that impact the treatment effect estimates is critical for both trial design and critical appraisal in the era of evidence-based medicine. In this updated systematic review, we collected additional empirical evidence about the associations between trial-level characteristics and treatment effect estimates. Authors of RCTs are suggested to account for trial characteristic that are significantly associated with treatment effect estimates, like sequence generation, allocation concealment, blinding and sample size when designing and conducting RCTs. When it is difficult to blind outcome assessors, a multiple assessors consensus strategy could be an alternative approach to reduce detection bias. When assessing the impact of blinding on treatment effect estimates in ME studies, combing the three key parties (participants, personnel and outcome assessors) of blinding as one group might reduce potential confounding.

## Conclusions

We found consistently significant associations between treatment effect estimates and sequence generation, allocation concealment, double blinding and sample size. The associations between treatment effect estimates and allocation concealment and double blinding were more consistent in trials using subjective outcomes. More ME studies are needed to assess the impact of trial characteristics in the Cochrane RoB2 tool without sufficient empirical evidence supported currently, including missing outcome data, intention-to-treat, methods used for outcome measures and selection of the reported results from multiple outcome measures or multiple analysis based on results (e.g., significance of the results). Furthermore, the methodological and reporting quality of included ME studies are dissatisfactory. Future researchers are recommended to reporting ME studies following the corresponding guideline [32]. Specific guidelines for conducting ME studies and assessing the methodological quality of ME studies are needed as well.

## Supplementary Information

**Additional file 1:** **Appendix 1.** Search strategy.

**Additional file 2:** **Appendix 2.** Bibliographical characteristics.

**Additional file 3:** **Appendix 3.** Methodological quality of meta-epidemiological (ME) studies.

**Additional file 4:** **Appendix 4.** List of included 80 meta-epidemiological studies.

**Additional file 5:** **Appendix 5.** List of excluded meta-epidemiological (ME) studies based on full text with reasons.

**Additional file 6:** **Appendix 6.** Number of meta-epidemiological studies on trial-level characteristics related to treatment effect estimates published by year.

**Additional file 7:** **Appendix 7.** Main characteristics of 80 meta-epidemiological (ME) studies.

**Additional file 8:** **Appendix 8.** Trial-level characteristics evaluated in 80 meta-epidemiological (ME) studies by chroNological order.

**Additional file 9:** **Appendix 9.** Details on the subgroup analyses in 48 meta-epidemiological (ME) studies.

**Additional file 10:** **Appendix 10.** Associations between treatment effect estimates and other trial-level characteristics for binary outcome.

**Additional file 11:** **Appendix 11.** Associations between treatment effect estimates and trial-level characteristics according to different subgroup analyses.

**Additional file 12:** **Appendix 12.** Results of additional subgroup analyses.

## Data Availability

The datasets used and/or analyzed during the current study are available from the corresponding author on reasonable request.

## Abbreviations

ME: Meta-epidemiological
RoB2: Cochrane risk-of-bias 2
RCT: Randomized controlled trial
PRISMA: Preferred Reporting Items for Systematic Reviews and Meta-Analyses
ICD-11: International Classification of Diseases 11th version
ROR: Ratio of odds ratio
AMSTAR 2: A MeaSurement Tool to Assess systematic Reviews-2
CI: Confidence interval
SMD: Standardized mean difference
DAG: Directed acyclic graph
