# Supplementary material for: Trial-level characteristics associate with treatment effect estimates: a systematic review of meta-epidemiological studies
Source: BMC Med Res Methodol. 2022 Jun 15;22:171. doi: 10.1186/s12874-022-01650-5 (PMC9202161; doi:10.1186/s12874-022-01650-5)
Supplement: Supplementary file 1 — Additional file 1: Appendix 1. Search strategy. [file 12874_2022_1650_MOESM1_ESM.docx]

**Appendix 1 Search strategy**

**PubMed**

| 1 | Search: meta-epidemiological[Title/Abstract] | 171 |
| --- | --- | --- |
| 2 | Search: meta-epidemiologic[Title/Abstract] | 43 |
| 3 | Search: metaepidemiological[Title/Abstract] | 179 |
| 4 | Search: metaepidemiologic[Title/Abstract] | 48 |
| 5 | Search: meta-epidemiology[Title/Abstract] | 47 |
| 6 | Search: (empirical[Title]) AND (bias[Title]) | 83 |
| 7 | Search: (comparison[Title]) AND (intervention effect[Title]) | 3 |
| 8 | Search: (comparison[Title]) AND (intervention effects[Title]) | 3 |
| 9 | Search: (comparison[Title]) AND (treatment effect[Title]) | 40 |
| 10 | Search: (comparison[Title]) AND (treatment effects[Title]) | 59 |
| 11 | Search: (impact[Title]) AND (intervention effect[Title]) | 2 |
| 12 | Search: (impact[Title]) AND (intervention effects[Title]) | 4 |
| 13 | Search: (impact[Title]) AND (treatment effect[Title]) | 27 |
| 14 | Search: (impact[Title]) AND (treatment effects[Title]) | 21 |
| 15 | Search: (influence[Title]) AND (intervention effect[Title]) | 3 |
| 16 | Search: (influence[Title]) AND (intervention effects[Title]) | 3 |
| 17 | Search: (influence[Title]) AND (treatment effect[Title]) | 19 |
| 18 | Search: (influence[Title]) AND (treatment effects[Title]) | 13 |
| 19 | Search: (investigating[Title]) AND (bias[Title]) | 73 |
| 20 | Search: (impact[Title]) AND (study characteristics[Title]) | 4 |
| 21 | Search: (impact[Title]) AND (conclusion*[Title]) | 56 |
| 22 | 1 OR 2 OR 3 OR 4 OR 5 OR 6 OR 7 OR 8 OR 9 OR 10 OR 11 OR 12 OR 13 OR 14  OR 15 OR 16 OR 17 OR 18 OR 19 OR 20 OR 21 Filters: from 2015/1/1 - 2020/7/25 | 354 |

**Embase**

| 1 | 'meta epidemiological':ab,ti | 192 |
| --- | --- | --- |
| 2 | 'meta epidemiologic':ab,ti | 42 |
| 3 | metaepidemiological:ab,ti | 12 |
| 4 | metaepidemiologic:ab,ti | 8 |
| 5 | 'meta epidemiology':ab,ti | 19 |
| 6 | empirical:ti AND bias:ti | 91 |
| 7 | comparison:ti AND 'intervention effect':ti | 2 |
| 8 | comparison:ti AND 'intervention effects':ti | 2 |
| 9 | comparison:ti AND 'treatment effect':ti | 49 |
| 10 | comparison:ti AND 'treatment effects':ti | 72 |
| 11 | impact:ti AND 'intervention effect':ti | 4 |
| 12 | impact:ti AND 'intervention effects':ti | 5 |
| 13 | impact:ti AND 'treatment effect':ti | 45 |
| 14 | impact:ti AND 'treatment effects':ti | 30 |
| 15 | influence:ti AND 'intervention effect':ti | 5 |
| 16 | influence:ti AND 'intervention effects':ti | 4 |
| 17 | influence:ti AND 'treatment effect':ti | 30 |
| 18 | influence:ti AND 'treatment effects':ti | 24 |
| 19 | investigating:ti AND bias:ti | 81 |
| 20 | impact:ti AND 'study characteristics':ti | 5 |
| 21 | impact:ti AND conclusion$:ti | 62 |
| 22 | #1 OR #2 OR #3 OR #4 OR #5 OR #6 OR #7 OR #8 OR #9 OR #10 OR #11 OR #12 OR #13 OR #14 OR #15 OR #16 OR #17 OR #18 OR #19 OR #20 OR #21 | 663 |
| 23 | (#1 OR #2 OR #3 OR #4 OR #5 OR #6 OR #7 OR #8 OR #9 OR #10 OR #11 OR #12 OR #13 OR #14 OR #15 OR #16 OR #17 OR #18 OR #19 OR #20 OR #21) AND [1-1-2015]/sd NOT [25-7-2020]/sd | 404 |

**Web of science**

| 1 | TI=meta-epidemiological OR AB=meta-epidemiological | 165 |
| --- | --- | --- |
| 2 | TI=meta-epidemiologic OR AB=meta-epidemiologic | 36 |
| 3 | TI=metaepidemiological OR AB=metaepidemiological | 6 |
| 4 | TI=metaepidemiologic OR AB=metaepidemiologic | 5 |
| 5 | TI=meta-epidemiology OR AB=meta-epidemiology | 14 |
| 6 | TI=empirical AND TI=bias | 273 |
| 7 | TI=comparison AND TI=intervention effect | 104 |
| 8 | TI=comparison AND TI=treatment effect | 910 |
| 9 | TI=impact AND TI=intervention effect | 93 |
| 10 | TI=impact AND TI=treatment effect | 505 |
| 11 | TI=influence AND TI=intervention effect | 58 |
| 12 | TI=influence AND TI=treatment effect | 338 |
| 13 | TI=investigating AND TI=bias | 229 |
| 14 | TI=impact AND TI=study characteristics | 497 |
| 15 | TI=impact AND TI=conclusion* | 91 |
| 16 | #15 OR #14 OR #13 OR #12 OR #11 OR #10 OR #9 OR #8 OR #7 OR #6 OR #5 OR #4 OR #3 OR #2 OR #1 | 2090 |
| 17 | #16  *IC Timespan=2015-2020* | 1887 |
