# Supplementary material for: Trial-level characteristics associate with treatment effect estimates: a systematic review of meta-epidemiological studies
Source: BMC Med Res Methodol. 2022 Jun 15;22:171. doi: 10.1186/s12874-022-01650-5 (PMC9202161; doi:10.1186/s12874-022-01650-5)
Supplement: Supplementary file 2 — Additional file 2: Appendix 2. Bibliographical characteristics. [file 12874_2022_1650_MOESM2_ESM.docx]

**Appendix 2 Bibliographical characteristics**

1. **General characteristics of reports of meta-epidemiological (ME) studies**
2. Year of publication: ______
3. Journal name: ______
4. Type of publication: ______

1. Journal article: ______

1) General journal

2) Medical specialty journal

Epidemiology/biostatistics

2. Agency report: _____ (Agency for Healthcare Research and Quality, Health Technology Assessment, et al.)

1. Involvement of epidemiologists/statisticians: ______

0=No; 1=Yes; 2=Not reported authors’ departments

1. Funding sources: ______

1=Public (Funding from public sources, such as government);

2=Private (Funding from private sources, such as businesses, foundations societies, and associations);

3=None;

4=Not reported;

5=Both 1 and 2

1. Type of intervention: ______

1=Pharmacological intervention; 2=Non-pharmacological intervention; 3=Both; 4= Not reported

1. Medical conditions: ______（[International](javascript:;) [Classification](javascript:;) [of](javascript:;) [Diseases](javascript:;), ICD-11）

1=Certain infectious or parasitic diseases;

2=Neoplasms;

3=Diseases of the blood or blood-forming organs;

4=Diseases of the immune system;

5=Endocrine, nutritional or metabolic diseases;

6=Mental, behavioral or neurodevelopmental disorders;

7=Sleep-wake disorders;

8=Diseases of the nervous system;

9=Diseases of the visual system;

10=Diseases of the ear or mastoid process;

11=Diseases of the circulatory system;

12=Diseases of the respiratory system;

13=Diseases of the digestive system;

14=Diseases of the skin;

15=Diseases of the musculoskeletal system or connective tissue;

16=Diseases of the genitourinary system;

17=Conditions related to sexual health;

18=Pregnancy, childbirth or the puerperium;

19=Certain conditions originating in the perinatal period;

20=Developmental anomalies;

21=Symptoms, signs or clinical findings, not elsewhere classified;

22=Injury, poisoning or certain other consequences of external causes;

23=External causes of morbidity or mortality;

24=Factors influencing health status or contact with health services;

25=Various medical areas;

26=Not reported

1. Trial characteristics (and its corresponding definitions) that were evaluated in the ME study: ______
2. Allocation concealment
3. Sequence generation
4. Double blinding [Described as double-blinding or ≥2 key groups (participants, personnel, outcome assessors) were blinded]
5. Drop out
6. Parallel group vs cross-over
7. Publication status (published trial vs gray literature)
8. Language (English language vs language other than English)
9. Study question well defined in introduction or methods
10. Placebo-control
11. Multi-center vs single-center
12. US vs other country
13. Adequate selection criteria reported
14. Randomization methodology criteria reported
15. Blinding of participants
16. Blinding of personnel
17. Blinding of outcome assessors
18. Valid statistical methods
19. Statistician involvement
20. Intention to treat analysis
21. Power calculation reported
22. Baseline characteristics reported
23. Baseline imbalance
24. Confounders accounted for
25. Dropouts reported
26. Reason for dropouts given
27. Percentage of dropouts
28. Findings support conclusion
29. Medline-indexed
30. Sample size
31. Power calculation adequate
32. Exclusion of participants
33. Trial publication date
34. Co-intervention
35. Compliance
36. Similar timing of the outcome assessment
37. Early stopping
38. Adult RCT vs children RCT
39. Publication in high-impact general medical journal vs other journals
40. Description of blinding procedure
41. Description of dropouts
42. Prespecification of alpha error
43. Prespecification of beta error
44. Less developed vs more developed countries
45. Elderly RCT vs adult RCT
46. Underpowered: power <50% vs adequately powered
47. Incomplete outcome data
48. Selective outcome reporting
49. Preintervention
50. Industry funding
51. Crossover
52. Sufficient follow-up
53. Other sources of bias
54. Overall risk of bias
55. Competing interest
56. Equal randomization
57. Parallel RCT vs split-mouth RCT
58. PubMed indexed vs non-PubMed indexed
59. Trial registration
60. First trial vs subsequent trials
61. Adjudication committee vs on-site assessment
62. Blinding of data analyst
63. Overall adequacy of blinding
64. Method of blinding (appropriate vs inappropriate or unclear)
65. Individual RCT vs cluster RCT
66. Registry-based RCT (RRCTs) vs conventional RCT (CRCTs)
67. Outcomes measured in other ways vs patient-reported outcome measures (PROMs)
68. All language publication vs English language publication
69. Non inferiority trial vs superiority trial
70. First trial vs overall trials
71. Primary outcomes adjustment
72. Type of outcome measure: ______

1=Binary; 2=Continuous; 3=Both 1 and 2; 4=Time-to-event; 5=Both 1,2 and 4

1. Type of study design for literature search: ______

1=Collection of meta-analysis

2=Collection of trials

3=Combination of previously published ME studies

1. Whether the ME study did quantitatively synthesize a difference of treatment effect: _____

0=No; 1=Yes

1. **C****haracteristics of ME studies based on collection of meta-analyses**
2. Data sources: ______

1=Cochrane review only;

2=Non-Cochrane review only;

2.1=Non-Cochrane review published in “high impact factor” journals;

3=Not reported the type of review;

4=Both Cochrane and non-Cochrane review;

4.1=Non-Cochrane review published in “high impact factor” journals and Cochrane review;

5=Other type of review ____

1. Type of meta-analyses: ______

1=Aggregated data only;

2=Aggregated and individual participant data (IPD);

3=IPD;

4=Network of aggregated data only;

5=Network of IPD;

1. Management of overlapping meta-analyses: ______

0=No; 1=Yes;

1. if Yes, how? ______
2. Reported minimum number of trials included in each meta-analysis: ______

0=No; 1=Yes;

1. if Yes, minimum number is ______
2. Criteria of selecting one meta-analysis within each systematic review: ______

1=Primary outcome; 2=Largest number of studies; 3=First outcome;

4=First outcome statistically significant; 5=Mortality;

6=Most clinically relevant; 7=Most homogeneous; 8=Objective;

9=At random; 10=No selection, all included; 11=Not reported;

12=Others_____

1. Data extraction sources: ______

1=From the report of each individual trial;

2=From the report of each systematic review;

3=Both;

4=NR

1. **Characteristics of ME studies quantitatively synthesized a difference of treatment effect estimates**
2. Which model the ME study used to combine: ______

1=Two-step approach (within-meta-analysis comparison and combination);

2=Two-step approach (within-trial comparison and combination);

3=Logistic regression;

4=Meta-regression;

5=Multilevel model;

6=Bayesian multilevel model;

7=Other______

8=NA

1. how to describe? ______
2. Whether the author accounted for clustering of trials within meta-analysis: ______

0=No; 1=Yes; 2=NA

1. if Yes, how: ______
2. Used random effect models to account for variability across meta-analyses/trials: ______

0=No; 1=Yes; 2=Not reported; 3=NA

1. Whether the author adjusted meta-confounders: ______

0=No; 1=Yes; 2=NA

1. If Yes, as main analysis

0=No; 1=Yes

1. If Yes, how: ______

1=Based on subgroup analysis solely;

2=Based on multiple variable analysis solely;

3=Both

1. Whether the author assessed the heterogeneity during analysis: ______

0=No; 1=Yes; 2=NA

1. If Yes, how: ______

1=Based on qualitative domain solely (chi-square test);

2=Based on quantitative domain solely (I^2^, τ^2^, ϕ^2^, F test);

3=Both;

1. Whether the author clearly reported the direction of interpretation of results: _____

0=No; 1=Yes; 2=NA

1. Whether the author performed subgroup analyses: ______

0=No; 1=Yes; 2=NA

1. If Yes, based on: ______

1=trial-level characteristics;

2=meta-analysis-level characteristics;

3=both;

4=Other______

1. If Yes, effect size of each subgroup analyses: ______
2. The effect size and 95% confidence intervals (CIs) for each characteristic: ______
