# Supplementary material for: Trial-level characteristics associate with treatment effect estimates: a systematic review of meta-epidemiological studies
Source: BMC Med Res Methodol. 2022 Jun 15;22:171. doi: 10.1186/s12874-022-01650-5 (PMC9202161; doi:10.1186/s12874-022-01650-5)
Supplement: Supplementary file 3 — Additional file 3: Appendix 3. Methodological quality of meta-epidemiological (ME) studies. [file 12874_2022_1650_MOESM3_ESM.docx]

**Appendix 3 Methodological quality of meta-epidemiological (ME) studies**

1. Did the author state that they had published a protocol prior to the conduct of the ME study? (AMSTAR 2, item 2)

0=No; 1=Yes

2. Did the author use a comprehensive literature search strategy? (Dechartres and colleagues’ criterion)

0=No; 1=Yes

1. If Yes, databases searched: ______

3. Did the author give a clear description of inclusion criteria and reasons for exclusion? (Dechartres and colleagues’ criterion)

0=No; 1=Yes

4. Whether selection process was reported? (Dechartres and colleagues’ criterion)

0=No; 1=Yes

5. Did the author perform selection process in duplicate? (Dechartres and colleagues’ criterion)

0=No; 1=Yes

6. Did the author perform data extraction in duplicate? (Dechartres and colleagues’ criterion)

0=No;

1=Fully of partly in duplicate;

2=Checking by a second reviewer;

3=Not reported;

4=Mentioned contact to authors

7. Did the author provide a list of excluded studies and justify the exclusions? (AMSTAR 2, item 7)

0=No; 1=Partial yes; 2=Yes

8. Did the author evaluate the heterogeneity between meta-analyses or trials or ME studies? (Dechartres and colleagues’ criterion)

0=No; 1=Yes; 2=Not applicable

i. Did the author perform an investigation of sources of any heterogeneity? (AMSTAR 2, Item 14)

0=No; 1=Yes; 2=Not applicable (There was no heterogeneity or the study did not evaluate the heterogeneity)

9. Whether analysis was adjusted on meta-confounders for ME studies estimating a combined difference of treatment effect? (Dechartres and colleagues’ criterion)

0=No; 1=Yes; 2=Not applicable

10. Whether clustering of trials within meta-analyses was taken into account for ME studies based on a collection of meta-analyses or previous published ME studies? (Dechartres and colleagues’ criterion)

0=No; 1=Yes; 2=Not applicable

11. Did the author report any potential sources of conflict of interest, including any funding they received for conducting the ME study? (AMSTAR 2, Item 16)

0=No; 1=Yes

12. Whether checking experimental and control arms were reported? (Dechartres and colleagues’ criterion)

0=No; 1=Yes

13. Whether the author reclassified of outcomes reported to have the same sense of interpretation? (Dechartres and colleagues’ criterion)

0=No; 1=Yes

14. Did the author give a clear definition of trial characteristics evaluated in ME studies? (Dechartres and colleagues’ criterion)

0=No; 1=Yes

15. Did the author assess the trial characteristics evaluated in duplicate? (Dechartres and colleagues’ criterion)

0=No; 1=Yes

16. Did the author assess the methodological quality of the included ME studies or meta-analyses or trials? (AMSTAR 2, Item 9)

0=No; 1=Yes
