# Supplementary material for: Trial-level characteristics associate with treatment effect estimates: a systematic review of meta-epidemiological studies
Source: BMC Med Res Methodol. 2022 Jun 15;22:171. doi: 10.1186/s12874-022-01650-5 (PMC9202161; doi:10.1186/s12874-022-01650-5)
Supplement: Supplementary file 4 — Additional file 4: Appendix 4. List of included 80 meta-epidemiological studies. [file 12874_2022_1650_MOESM4_ESM.docx]

**Appendix 4 List of included 80 meta-epidemiological studies**

1. Schulz KF, Chalmers I, HaYes RJ, Altman DG. Empirical evidence of bias. Dimensions of methodological quality associated with estimates of treatment effects in controlled trials. Jama. 1995;273(5):408-12.

2. Khan KS, Daya S, Collins JA, Walter SD. Empirical evidence of bias in infertility research: overestimation of treatment effect in crossover trials using pregnancy as the outcome measure. Fertility and sterility. 1996;65(5):939-45.

3. Moher D, Cook DJ, Jadad AR, Tugwell P, Moher M, Jones A, et al. Assessing the quality of reports of randomised trials: implications for the conduct of meta-analyses. Health techNology assessment (Winchester, England). 1999;3(12):i-iv, 1-98.

4. McAuley L, Pham B, Tugwell P, Moher D. Does the inclusion of grey literature influence estimates of intervention effectiveness reported in meta-analyses? Lancet. 2000;356(9237):1228-31.

5. Moher D, Pham B, Klassen TP, Schulz KF, Berlin JA, Jadad AR, et al. What contributions do languages other than English make on the results of meta-analyses? Journal of clinical epidemiology. 2000;53(9):964-72.

6. Kjaergard LL, Villumsen J, Gluud C. Reported methodologic quality and discrepancies between large and small randomized trials in meta-analyses. Annals of internal medicine. 2001;135(11):982-9.

7. Balk EM, Bonis PA, Moskowitz H, Schmid CH, Ioannidis JP, Wang C, et al. Correlation of quality measures with estimates of treatment effect in meta-analyses of randomized controlled trials. Jama. 2002;287(22):2973-82.

8. Jüni P, Holenstein F, Sterne J, Bartlett C, Egger M. Direction and impact of language bias in meta-analyses of controlled trials: empirical study. International journal of epidemiology. 2002;31(1):115-23.

9. Egger M, Juni P, Bartlett C, Holenstein F, Sterne J. How important are comprehensive literature searches and the assessment of trial quality in systematic reviews? Empirical study. Health techNology assessment (Winchester, England). 2003;7(1):1-76.

10. Hopewell S, McDonald S, Clarke M, Egger M. Grey literature in meta-analyses of randomized trials of health care interventions. The Cochrane database of systematic reviews. 2007(2):Mr000010.

11. Sampson M, Barrowman NJ, Moher D, Klassen TP, Pham B, Platt R, et al. Should meta-analysts search Embase in addition to Medline? Journal of clinical epidemiology. 2003;56(10):943-55.

12. Contopoulos-Ioannidis DG, Gilbody SM, TrikaliNos TA, Churchill R, Wahlbeck K, Ioannidis JP. Comparison of large vs smaller randomized trials for mental health-related interventions. The American journal of psychiatry. 2005;162(3):578-84.

13. Pham B, Klassen TP, Lawson ML, Moher D. Language of publication restrictions in systematic reviews gave different results depending on whether the intervention was conventional or complementary. Journal of clinical epidemiology. 2005;58(8):769-76.

14. Tierney JF, Stewart LA. Investigating patient exclusion bias in meta-analysis. International journal of epidemiology. 2005;34(1):79-87.

15. Lathyris DN, TrikaliNos TA, Ioannidis JP. Evidence from crossover trials: empirical evaluation and comparison against parallel arm trials. International journal of epidemiology. 2007;36(2):422-30.

16. Pildal J, Hróbjartsson A, Jørgensen KJ, Hilden J, Altman DG, Gøtzsche PC. Impact of allocation concealment on conclusions drawn from meta-analyses of randomized trials. International journal of epidemiology. 2007;36(4):847-57.

17. Siersma V, Als-Nielsen B, Chen W, Hilden J, Gluud LL, Gluud C. Multivariable modelling for meta-epidemiological assessment of the association between trial quality and treatment effects estimated in randomized clinical trials. Statistics in medicine. 2007;26(14):2745-58.

18. Fenwick J, Needleman IG, Moles DR. The effect of bias on the magnitude of clinical outcomes in periodontology: a pilot study. Journal of clinical periodontology. 2008;35(9):775-82.

19. Wood L, Egger M, Gluud LL, Schulz KF, Jüni P, Altman DG, et al. Empirical evidence of bias in treatment effect estimates in controlled trials with different interventions and outcomes: meta-epidemiological study. BMJ (Clinical research ed). 2008;336(7644):601-5.

20. Nüesch E, Reichenbach S, Trelle S, Rutjes AW, Liewald K, Sterchi R, et al. The importance of allocation concealment and patient blinding in osteoarthritis trials: a meta-epidemiologic study. Arthritis and rheumatism. 2009;61(12):1633-41.

21. Nüesch E, Trelle S, Reichenbach S, Rutjes AW, Bürgi E, Scherer M, et al. The effects of excluding patients from the analysis in randomised controlled trials: meta-epidemiological study. BMJ (Clinical research ed). 2009;339:b3244.

22. Patsopoulos NA, Ioannidis JP. The use of older studies in meta-analyses of medical interventions: a survey. Open medicine : a peer-reviewed, independent, open-access journal. 2009;3(2):e62-8.

23. van Tulder MW, Suttorp M, Morton S, Bouter LM, Shekelle P. Empirical evidence of an association between internal validity and effect size in randomized controlled trials of low-back pain. Spine. 2009;34(16):1685-92.

24. Bassler D, Briel M, Montori VM, Lane M, Glasziou P, Zhou Q, et al. Stopping randomized trials early for benefit and estimation of treatment effects: systematic review and meta-regression analysis. Jama. 2010;303(12):1180-7.

25. Contopoulos-Ioannidis DG, Baltogianni MS, Ioannidis JP. Comparative effectiveness of medical interventions in adults vs children. The Journal of pediatrics. 2010;157(2):322-30.e17.

26. Nüesch E, Trelle S, Reichenbach S, Rutjes AW, Tschannen B, Altman DG, et al. Small study effects in meta-analyses of osteoarthritis trials: meta-epidemiological study. BMJ (Clinical research ed). 2010;341:c3515.

27. Dechartres A, Boutron I, Trinquart L, Charles P, Ravaud P. Single-center trials show larger treatment effects than multicenter trials: evidence from a meta-epidemiologic study. Annals of internal medicine. 2011;155(1):39-51.

28. Herbison P, Hay-Smith J, Gillespie WJ. Different methods of allocation to groups in randomized trials are associated with different levels of bias. A meta-epidemiological study. Journal of clinical epidemiology. 2011;64(10):1070-5.

29. Siontis KC, Evangelou E, Ioannidis JP. Magnitude of effects in clinical trials published in high-impact general medical journals. International journal of epidemiology. 2011;40(5):1280-91.

30. Bafeta A, Dechartres A, Trinquart L, Yavchitz A, Boutron I, Ravaud P. Impact of single centre status on estimates of intervention effects in trials with continuous outcomes: meta-epidemiological study. BMJ (Clinical research ed). 2012;344:e813.

31. Hempel S, Miles J, Suttorp MJ, Wang Z, Johnsen B, Morton S, et al. AHRQ Methods for Effective Health Care. Detection of Associations Between Trial Quality and Effect Sizes. Rockville (MD): Agency for Healthcare Research and Quality (US); 2012.

32. Hróbjartsson A, Thomsen AS, Emanuelsson F, Tendal B, Hilden J, Boutron I, et al. Observer bias in randomised clinical trials with binary outcomes: systematic review of trials with both blinded and Non-blinded outcome assessors. BMJ (Clinical research ed). 2012;344:e1119.

33. Mhaskar R, Djulbegovic B, Magazin A, Soares HP, Kumar A. Published methodological quality of randomized controlled trials does Not reflect the actual quality assessed in protocols. Journal of clinical epidemiology. 2012;65(6):602-9.

34. Savović J, Jones H, Altman D, Harris R, Jűni P, Pildal J, et al. Influence of reported study design characteristics on intervention effect estimates from randomised controlled trials: combined analysis of meta-epidemiological studies. Health techNology assessment (Winchester, England). 2012;16(35):1-82.

35. Chaimani A, Vasiliadis HS, Pandis N, Schmid CH, Welton NJ, Salanti G. Effects of study precision and risk of bias in networks of interventions: a network meta-epidemiological study. International journal of epidemiology. 2013;42(4):1120-31.

36. Dechartres A, Trinquart L, Boutron I, Ravaud P. Influence of trial sample size on treatment effect estimates: meta-epidemiological study. BMJ (Clinical research ed). 2013;346:f2304.

37. Hróbjartsson A, Thomsen AS, Emanuelsson F, Tendal B, Hilden J, Boutron I, et al. Observer bias in randomized clinical trials with measurement scale outcomes: a systematic review of trials with both blinded and Nonblinded assessors. CMAJ : Canadian Medical Association journal = journal de l'Association medicale canadienne. 2013;185(4):E201-11.

38. Panagiotou OA, Contopoulos-Ioannidis DG, Ioannidis JP. Comparative effect sizes in randomised trials from less developed and more developed countries: meta-epidemiological assessment. BMJ (Clinical research ed). 2013;346:f707.

39. Seegers V, Trinquart L, Boutron I, Ravaud P. Comparison of treatment effect estimates for pharmacological randomized controlled trials enrolling older adults only and those including adults: a meta-epidemiological study. PloS one. 2013;8(5):e63677.

40. Turner RM, Bird SM, Higgins JPT. The Impact of Study Size on Meta-analyses: Examination of Underpowered Studies in Cochrane Reviews. PloS one. 2013;8(3):e59202.

41. Unverzagt S, Prondzinsky R, Peinemann F. Single-center trials tend to provide larger treatment effects than multicenter trials: a systematic review. Journal of clinical epidemiology. 2013;66(11):1271-80.

42. Zhang Z, Xu X, Ni H. Small studies may overestimate the effect sizes in critical care meta-analyses: a meta-epidemiological study. Critical care (London, England). 2013;17(1):R2.

43. Bialy L, Vandermeer B, Lacaze-Masmonteil T, Dryden DM, Hartling L. A meta-epidemiological study to examine the association between bias and treatment effects in neonatal trials. Evidence-based child health : a Cochrane review journal. 2014;9(4):1052-9.

44. Dechartres A, Altman DG, Trinquart L, Boutron I, Ravaud P. Association between analytic strategy and estimates of treatment outcomes in meta-analyses. Jama. 2014;312(6):623-30.

45. Hartling L, Hamm MP, Fernandes RM, Dryden DM, Vandermeer B. Quantifying bias in randomized controlled trials in child health: a meta-epidemiological study. PloS one. 2014;9(2):e88008.

46. Hróbjartsson A, Emanuelsson F, Skou Thomsen AS, Hilden J, Brorson S. Bias due to lack of patient blinding in clinical trials. A systematic review of trials randomizing patients to blind and Nonblind sub-studies. International journal of epidemiology. 2014;43(4):1272-83.

47. Hróbjartsson A, Thomsen AS, Emanuelsson F, Tendal B, Rasmussen JV, Hilden J, et al. Observer bias in randomized clinical trials with time-to-event outcomes: systematic review of trials with both blinded and Non-blinded outcome assessors. International journal of epidemiology. 2014;43(3):937-48.

48. Lathyris D, Panagiotou OA, Baltogianni M, Ioannidis JP, Contopoulos-Ioannidis DG. Safety of medical interventions in children vs adults. Pediatrics. 2014;133(3):e666-73.

49. Papageorgiou SN, AntoNoglou GN, Tsiranidou E, Jepsen S, Jäger A. Bias and small-study effects influence treatment effect estimates: a meta-epidemiological study in oral medicine. Journal of clinical epidemiology. 2014;67(9):984-92.

50. Smaïl-Faugeron V, Fron-Chabouis H, Courson F, Durieux P. Comparison of intervention effects in split-mouth and parallel-arm randomized controlled trials: a meta-epidemiological study. BMC medical research methodology. 2014;14(1):64.

51. Abraha I, Cherubini A, CozzoliNo F, De Florio R, Luchetta ML, Rimland JM, et al. Deviation from intention to treat analysis in randomised trials and treatment effect estimates: Meta-epidemiological study. BMJ (Online). 2015;350.

52. Armijo-Olivo S, Saltaji H, Da Costa BR, Fuentes J, Ha C, Cummings GG. What is the influence of randomization sequence generation and allocation concealment on treatment effects of physical therapy trials? A meta-epidemiological study. BMJ open. 2015;5(9).

53. Halladay CW, TrikaliNos TA, Schmid IT, Schmid CH, Dahabreh IJ. Using data sources beyond PubMed has a modest impact on the results of systematic reviews of therapeutic interventions. Journal of clinical epidemiology. 2015;68(9):1076-84.

54. Papageorgiou SN, Xavier GM, Cobourne MT. Basic study design influences the results of orthodontic clinical investigations. Journal of clinical epidemiology. 2015;68(12):1512-22.

55. Dechartres A, Ravaud P, Atal I, Riveros C, Boutron I. Association between trial registration and treatment effect estimates: A meta-epidemiological study. BMC medicine. 2016;14(1).

56. Gartlehner G, Dobrescu A, Evans TS, Thaler K, Nussbaumer B, Sommer I, et al. Average effect estimates remain similar as evidence evolves from single trials to high-quality bodies of evidence: a meta-epidemiologic study. Journal of clinical epidemiology. 2016;69:16-22.

57. Koletsi D, Spineli LM, Lempesi E, Pandis N. Risk of bias and magnitude of effect in orthodontic randomized controlled trials: a meta-epidemiological review. European journal of orthodontics. 2016;38(3):308-12.

58. Ndounga Diakou LA, Trinquart L, Hróbjartsson A, Barnes C, Yavchitz A, Ravaud P, et al. Comparison of central adjudication of outcomes and onsite outcome assessment on treatment effect estimates. The Cochrane database of systematic reviews. 2016;3(3):Mr000043.

59. Page MJ, Higgins JPT, Clayton G, Sterne JAC, Hróbjartsson A, Savović J. Empirical evidence of study design biases in randomized trials: Systematic review of meta-epidemiological studies. PloS one. 2016;11(7).

60. Armijo-Olivo S, Fuentes J, da Costa BR, Saltaji H, Ha C, Cummings GG. Blinding in Physical Therapy Trials and Its Association with Treatment Effects: A Meta-epidemiological Study. American journal of physical medicine & rehabilitation. 2017;96(1):34-44.

61. Alahdab F, Farah W, Almasri J, Barrionuevo P, Zaiem F, Benkhadra R, et al. Treatment Effect in Earlier Trials of Patients With Chronic Medical Conditions: A Meta-Epidemiologic Study. Mayo Clinic proceedings. 2018;93(3):278-83.

62. Bolvig J, Juhl CB, Boutron I, Tugwell P, Ghogomu EAT, Pardo JP, et al. Some Cochrane risk-of-bias items are Not important in osteoarthritis trials: a meta-epidemiological study based on Cochrane reviews. Journal of clinical epidemiology. 2018;95:128-36.

63. Dechartres A, Atal I, Riveros C, Meerpohl J, Philippe R. Association between publication characteristics and treatment effect estimates a meta-epidemiologic study. Annals of internal medicine. 2018;169(6):385-93.

64. Janiaud P, Cristea I-A, Ioannidis JPA. Industry-funded vs Non-profit-funded critical care research: a meta-epidemiological overview (vol 44, pg 1613, 2018). Intensive care medicine. 2018;44(12):2323-.

65. Papageorgiou SN, Xavier GM, Cobourne MT, Eliades T. Registered trials report less beneficial treatment effects than unregistered ones: a meta-epidemiological study in orthodontics. Journal of clinical epidemiology. 2018;100:44-52.

66. Saltaji H, Armijo-Olivo S, Cummings GG, Amin M, da Costa BR, Flores-Mir C. Influence of blinding on treatment effect size estimate in randomized controlled trials of oral health interventions. BMC medical research methodology. 2018;18(1):42.

67. Saltaji H, Armijo-Olivo S, Cummings GG, Amin M, da Costa BR, Flores-Mir C. Impact of Selection Bias on Treatment Effect Size Estimates in Randomized Trials of Oral Health Interventions: A Meta-epidemiological Study. Journal of dental research. 2018;97(1):5-13.

68. Savovic J, Turner RM, Mawdsley D, Jones HE, BeyNon R, Higgins JPT, et al. Association Between Risk-of-Bias Assessments and Results of Randomized Trials in Cochrane Reviews: The ROBES Meta-Epidemiologic Study. American journal of epidemiology. 2018;187(5):1113-22.

69. de Almeida MO, Saragiotto BT, Maher C, Costa LOP. Allocation Concealment and Intention-To-Treat Analysis Do Not Influence the Treatment Effects of Physical Therapy Interventions in Low Back Pain Trials: a Meta-epidemiologic Study. Archives of physical medicine and rehabilitation. 2019;100(7):1359-66.

70. Hulshof TA, Zuidema SU, van Meer PJK, Gispen-de Wied CC, Luijendijk HJ. Baseline imbalances and clinical outcomes of atypical antipsychotics in dementia: A meta-epidemiological study of randomized trials. International journal of methods in psychiatric research. 2019;28(1):e1757.

71. Leyrat C, Caille A, Eldridge S, Kerry S, Dechartres A, Giraudeau B. Intervention effect estimates in cluster randomized vs individually randomized trials: A meta-epidemiological study. International journal of epidemiology. 2019;48(2):609-19.

72. Mathes T, Klassen P, Pieper D. No differences were found between effect estimates from conventional and registry-based randomized controlled trials. Journal of clinical epidemiology. 2019;105:80-91.

73. Armijo-Olivo S, Dennett L, Arienti C, Dahchi M, Arokoski J, Heinemann AW, et al. Blinding in Rehabilitation Research: Empirical Evidence on the Association Between Blinding and Treatment Effect Estimates. American journal of physical medicine & rehabilitation. 2020;99(3):198-209.

74. Berthelsen DB, Ginnerup-Nielsen E, Juhl C, Lund H, Henriksen M, Hróbjartsson A, et al. Controversy and Debate on Meta-epidemiology. Paper 1: Treatment effect sizes vary in randomized trials depending on the type of outcome measure. Journal of clinical epidemiology. 2020;123:27-38.

75. Haring R, Ghannad M, Bertizzolo L, Page MJ. No evidence found for an association between trial characteristics and treatment effects in randomized trials of testosterone therapy in men: a meta-epidemiological study. Journal of clinical epidemiology. 2020;122:12-9.

76. Moustgaard H, Clayton GL, Jones HE, Boutron I, Jørgensen L, Laursen DRT, et al. Impact of blinding on estimated treatment effects in randomised clinical trials: meta-epidemiological study. BMJ (Clinical research ed). 2020;368:l6802.

77. Nussbaumer-Streit B, Klerings I, Dobrescu AI, Persad E, Stevens A, Garritty C, et al. Excluding Non-English publications from evidence-syntheses did Not change conclusions: a meta-epidemiological study. Journal of clinical epidemiology. 2020;118:42-54.

78. Wang Z, Nayfeh T, Sofiyeva N, Ponte OJ, Rajjoub R, Malandris K, et al. Including Non-inferiority Trials in Contemporary Meta-analyses of Chronic Medical Conditions: a Meta-epidemiological Study. Journal of general internal medicine. 2020;35(7):2162-6.

79. Yi J, Li H, Li Y, Li X, Zheng W, Zhao Z. Risk of Bias and Its Impact on Intervention Effect Estimates of Randomized Controlled Trials in Endodontics. Journal of endodontics. 2020;46(1):12-8.

80. Saquib N, Saquib J, Ioannidis JP. Practices and impact of primary outcome adjustment in randomized controlled trials: meta-epidemiologic study. BMJ (Clinical research ed) 2013;347:f4313. doi: 10.1136/bmj.f4313 [published Online First: 2013/07/16]
