# Supplementary material for: Trial-level characteristics associate with treatment effect estimates: a systematic review of meta-epidemiological studies
Source: BMC Med Res Methodol. 2022 Jun 15;22:171. doi: 10.1186/s12874-022-01650-5 (PMC9202161; doi:10.1186/s12874-022-01650-5)
Supplement: Supplementary file 5 — Additional file 5: Appendix 5. List of excluded meta-epidemiological (ME) studies based on full text with reasons. [file 12874_2022_1650_MOESM5_ESM.docx]

**Appendix 5 List of excluded meta-epidemiological (ME) studies based on full text with reasons**

| **References** | **Reasons of exclusion** |
| --- | --- |
| 1. Cecilia-Joseph E, Auvert B, Broet P, Moreau T. Influence of trial duration on the bias of the estimated treatment effect in clinical trials when individual heterogeneity is igNored. Biometrical Journal. 2015;57(3):371-83. | Not a ME study |
| 2. Dong-il K, Soohyun L, eun lh. A Meta-analysis on the Characteristics and Effects of Meta-Cognitive Strategic Interventions in Korea: Comparison Between Group-Designed Studies and Single Case Studies. Asian Journal of Education. 2016;17(3):21-48. | Not a ME study |
| 3. Howick J, Hoffmann T. How placebo characteristics can influence estimates of intervention effects in trials. CMAJ : Canadian Medical Association journal = journal de l'Association medicale canadienne. 2018;190(30):E908-E11. | Not a ME study |
| 4. Luan JJ, Mani R, Hung HMJ. Comparison of Treatment Effects Between US and Non-US Study Sites in Multiregional Alzheimer Disease Clinical Trials. Therapeutic InNovation and Regulatory Science. 2016;50(1):66-73. | Not a ME study |
| 5. Page MJ, Forbes A, Chau M, Green SE, McKenzie JE. Investigation of bias in meta-analyses due to selective inclusion of trial effect estimates: Empirical study. BMJ open. 2016;6(4). | Not a ME study |
| 6. Papageorgiou SN, Tsiranidou E, AntoNoglou GN, Deschner J, Jäger A. Choice of effect measure for meta-analyses of dichotomous outcomes influenced the identified heterogeneity and direction of small-study effects. Journal of clinical epidemiology. 2015;68(5):534-41. | Not a ME study |
| 7. Ndounga Diakou LA, Trinquart L, Hróbjartsson A, Barnes C, Yavchitz A, Ravaud P, et al. Comparison of central adjudication of outcomes and onsite outcome assessment on treatment effect estimates. Cochrane Database of Systematic Reviews. 2016;2016(3). | Not a ME study |
| 8. Harrison EM, Bhangu A, Swann O, Wigmore SJ. Equivalence approach is more appropriate for comparison of treatment effect estimates. Annals of surgery. 2015;262(2):e67. | Not a ME study |
| 9. Ratib S, Wilkes SR, Grainge MJ, Thomas KS, Tobinska C, Williams HC. Is there an association between study size and reporting of study quality in dermatological clinical trials? A meta-epidemiological review. British Journal of Dermatology. 2017;176(6):1657-8. | Not a ME study |
| 10. Dechartres A, Trinquart L, Faber T, Ravaud P. Empirical evaluation of which trial characteristics are associated with treatment effect estimates. Journal of clinical epidemiology. 2016;77:24-37. | Not a ME study |
| 11. Ewald H, Speich B, Ladanie A, Bucher HC, Ioannidis JPA, Hemkens LG. Marginal structural models and other analyses allow multiple estimates of treatment effects in randomized clinical trials: Meta-epidemiological analysis. Journal of clinical epidemiology. 2019;107:12-26. | Not a ME study |
| 12. Kim M, Merrill JT, Wang C, Viswanathan S, Kalunian K, Hanrahan L, et al. SLE clinical trials: Impact of missing data on estimating treatment effects. Lupus Science and Medicine. 2019;6(1). | Not a ME study |
| 13. Anthon CT, Granholm A, Perner A, Laake JH, Møller MH. Overall bias and sample sizes were unchanged in ICU trials over time: a meta-epidemiological study. Journal of clinical epidemiology. 2019;113:189-99. | ME study not in the field of therapeutics |
| 14. Ballegaard C, Jørgensen TS, Skougaard M, Strand V, Mease PJ, Kristensen LE, et al. Trial Characteristics as Contextual Factors When Evaluating Targeted Therapies in Patients With Psoriatic Disease: A Meta-Epidemiologic Study. Arthritis Care and Research. 2018;70(8):1206-17. | ME study not in the field of therapeutics |
| 15. Ban J-W, Ignacio Emparanza J, Urreta I, Burls A. Design Characteristics Influence Performance of Clinical Prediction Rules in Validation: A Meta-Epidemiological Study. PloS one. 2016;11(1). | ME study not in the field of therapeutics |
| 16. Gartlehner G, Dobrescu A, Evans TS, Bann C, Robinson KA, Reston J, et al. The predictive validity of quality of evidence grades for the stability of effect estimates was low: A meta-epidemiological study. Journal of clinical epidemiology. 2016;70:52-60. | ME study not in the field of therapeutics |
| 17. Liu P, Ioannidis JPA, Ross JS, Dhruva SS, Luxkaranayagam AT, Vasiliou V, et al. Age-treatment subgroup analyses in Cochrane intervention reviews: A meta-epidemiological study. BMC medicine. 2019;17(1). | ME study not in the field of therapeutics |
| 18. Cottin J, Atzenhoffer M, Gouraud A, Bérard A, Cucherat M. #41 Pregnancy and drugs: observational studies risk of bias assessment (PANDORA). A meta-epidemiological pilot study. Reproductive Toxicology. 2019;88:147. | ME study not in the field of therapeutics |
| 19. Kurian SJ, Wahood W, Alvi MA, Yolcu YU, Zreik J, Bydon M. Assessing the Effects of Publication Bias on Reported Outcomes of Cervical Disc Replacement and Anterior Cervical Discectomy and Fusion: A Meta-Epidemiologic Study. World neurosurgery. 2020;137:443-50.e13. | ME study not in the field of therapeutics |
| 20. Luo S, Long Y, Xiao W, Wang X, Chen R, Guo Q, et al. Risk of bias assessments and reporting quality of systematic reviews and randomized controlled trials examining acupuncture for depression: An overview and meta-epidemiology study. Journal of evidence-based medicine. 2020;13(1):25-33. | ME study not in the field of therapeutics |
| 21. Damen JAAG, Debray TPA, Pajouheshnia R, Reitsma JB, Scholten RJPM, Moons KGM, et al. Empirical evidence of the impact of study characteristics on the performance of prediction models: A meta-epidemiological study. BMJ open. 2019;9(4). | ME study not in the field of therapeutics |
| 22. Devos F, Foissac F, Bouazza N, Ancel P-Y, Treluyer J-M, Chappuy H. Study characteristics impacted the pragmatism of randomized controlled trial published in nursing: a meta-epidemiological study. Journal of clinical epidemiology. 2019;116:18-25. | ME study not in the field of therapeutics |
| 23. Arienti C, StefaNo N, Da costa B, Armijo-Olivo S. The influence of sponsorship on the treatment effects of trials. International Journal of TechNology Assessment in Health Care. 2019;35:76. | Conference abstract |
| 24. Armijo-Olivo S, Da costa B, Arienti C, StefaNo N. Use of intention to treat and magnitude of treatment effects. International Journal of TechNology Assessment in Health Care. 2019;35:12-3. | Conference abstract |
| 25. Chen T, Qin R, Wang D, Cornelius V. Influence of primary outcome change on treatment effect estimates in clinical trials: Meta-epidemiological study. Trials. 2017;18. | Conference abstract |
| 26. Gartlehner G. Assessing the predictive validity of quality of evidence grades in systematic reviews. A meta-epidemiological study. Value in Health. 2016;19(3):A74. | Conference abstract |
| 27. Joseph R, Sim J, Ogollah R, Lewis M. An empirical evaluation of the impact of missing data on treatment effect. Trials. 2015;16. | Conference abstract |
| 28. Kim M, Merrill JT, Kalunian KC, Hanrahan L, Izmirly PM. Missing outcomes in sle clinical trials: Impact on estimating treatment effects. Lupus Science and Medicine. 2018;5:A48. | Conference abstract |
| 29. Oh SJ, Rezaie A, Pimentel M. EVALUATION OF THE ROLE OF SAMPLE SIZE IN DETERMINING THE TRUE EFFICACY OF DRUGS IN IBS: A META-EPIDEMIOLOGICAL ANALYSIS OF RANDOMIZED CONTROLLED TRIALS (RCTS). Gastroenterology. 2018;154(6):S-982. | Conference abstract |
| 30. Sanchez-Ramos L, Roeckner J, Knupp R, Kaunitz AM. Small studies in meta-analyses overestimate treatment effects of aspirin for the prevention of pre-clampsia: A systematic review and meta-epidemiological study. American Journal of Obstetrics and Gynecology. 2018;218(1):S189-S90. | Conference abstract |
| 31. Agreement of treatment effects for mortality from routinely collected data and subsequent randomized trials: meta-epidemiological survey (vol 352, i493, 2016). Bmj-British Medical Journal. 2018;362. | Duplication |
| 32. Diakou LAN, Trinquart L, Hrobjartsson A, Barnes C, Yavchitz A, Ravaud P, et al. Comparison of central adjudication of outcomes and onsite outcome assessment on treatment effect estimates. Cochrane Database of Systematic Reviews. 2016(3). | Duplication |
| 33. Janiaud P, Cristea IA, Ioannidis JPA. Correction to: Industry-funded vs non-profit-funded critical care research: a meta-epidemiological overview. Intensive care medicine. 2018;44(12):2323. | Duplication |
| 34. Moher D, Pham B, Jones A, Cook DJ, Jadad AR, Moher M, et al. Does quality of reports of randomised trials affect estimates of intervention efficacy reported in meta-analyses? Lancet. 1998;352(9128):609-13. | Duplication |
| 35. Savović J, Jones HE, Altman DG, Harris RJ, Jüni P, Pildal J, et al. Influence of reported study design characteristics on intervention effect estimates from randomized, controlled trials. Annals of internal medicine. 2012;157(6):429-38. | Duplication |
| 36. Hempel S, Suttorp MJ, Miles JNV, Wang Z, Maglione M, Morton S, et al. AHRQ Methods for Effective Health Care. Empirical Evidence of Associations Between Trial Quality and Effect Size. Rockville (MD): Agency for Healthcare Research and Quality (US); 2011. | Duplication |
| 37. Moher D, Pham B, Lawson ML, Klassen TP. The inclusion of reports of randomised trials published in languages other than English in systematic reviews. Health technology assessment (Winchester, England). 2003;7(41):1-90. | Duplication |
| 38. Gartlehner G, Dobrescu A, Evans TS, Thaler K, Nussbaumer B, Sommer I, et al. Average effect estimates remain similar as evidence evolves from single trials to high-quality bodies of evidence: A meta-epidemiologic study. Journal of clinical epidemiology. 2016;69:16-22. | Duplication |
| 39. Smail-Faugeron V, Fron-Chabouis H, Courson F, Durieux P. Comparison of intervention effects in split-mouth and parallel-arm randomized controlled trials: a meta-epidemiological study (vol 14, 64, 2014). BMC medical research methodology. 2015;15. | Duplication |
| 40. Christensen AW, Tarp S, Furst DE, Dossing A, Amris K, Bliddal H, et al. Most Trial Eligibility Criteria and Patient Baseline Characteristics Do not Modify Treatment Effect in Trials Using Targeted Therapies for Rheumatoid Arthritis: A Meta-Epidemiological Study. PloS one. 2015;10(9). | ME study not based on trial-level characteristics |
| 41. Lanza A, Ravaud P, Riveros C, Dechartres A. Comparison of estimates between cohort and case-control studies in meta-analyses of therapeutic interventions: A meta-epidemiological study. PloS one. 2016;11(5). | ME study not based on trial-level characteristics |
| 42. Bandak E, Christensen R, Boesen M, Bliddal H, Altman RD, Hunter D, et al. Exercise vs intra-articular saline for knee osteoarthritis pain: a meta-epidemiological study with focus on comparative effectiveness. Osteoarthritis and Cartilage. 2019;27:S212-S3. | ME study not based on trial-level characteristics |
| 43. Nielsen SM, Storgaard H, Ellingsen T, Shea BJ, Wells GA, Welch VA, et al. Population characteristics as important contextual factors in rheumatological trials: an exploratory meta-epidemiological study from an OMERACT Working Group. Annals of the rheumatic diseases. 2020. | ME study not based on trial-level characteristics |
| 44. Kim M, Merrill J, Kalunian KC, Hanrahan L, Izmirly PM. Missing Outcomes in SLE Clinical Trials: Impact on Estimating Treatment Effects. Arthritis & Rheumatology. 2018;70. | ME study not based on trial-level characteristics |
| 45. Koster TM, Wetterslev J, Gluud C, Jakobsen JC, Kaufmann T, Eck RJ, et al. Apparently conclusive meta-analyses on interventions in critical care may be inconclusive-a meta-epidemiological study. Journal of clinical epidemiology. 2019;114:1-10. | ME study Not based on trial-level characteristics |
| 46. Wallach JD, Ciani O, Pease AM, Gonsalves GS, Krumholz HM, Taylor RS, et al. Comparison of treatment effect sizes from pivotal and postapproval trials of novel therapeutics approved by the FDA based on surrogate markers of disease: A meta-epidemiological study. BMC medicine. 2018;16(1). | ME study not based on trial-level characteristics |
| 47. Rejon-Parrilla JC, Salcher-Konrad M, Nguyen M, Davis K, Jonsson P, Naci H. Can we rely on non-randomised studies? Findings from a meta-epidemiological review. European Journal of Public Health. 2019;29. | ME study compared RCT with observational study |
| 48. Coscia C, Jaureguizar A, Quezada CA, Muriel A, Monreal M, Villén T, et al. Comparison of All-Cause Mortality Following VTE Treatment Between Propensity Score-Adjusted Observational Studies and Matched Randomized Controlled Trials: Meta-Epidemiologic Study. Chest. 2019;155(4):689-98. | ME study compared RCT with observational study |
| 49. Hemkens LG, Contopoulos-Ioannidis DG, Ioannidis JP. Agreement of treatment effects for mortality from routinely collected data and subsequent randomized trials: meta-epidemiological survey. BMJ (Clinical research ed). 2016;352:i493. | ME study compared RCT with observational study |
| 50. Jacobs WC, Kruyt MC, Verbout AJ, Oner FC. Effect of methodological quality measures in spinal surgery research: a metaepidemiological study. The spine journal : official journal of the north American Spine Society. 2012;12(4):339-48. | ME study included observational study |
| 51. Bolvig J, Juhl CB, Boutron I, Tugwell P, Ghogomu EA, Pardo Pardo J, et al. Assessing bias in osteoarthritis trials included in cochrane reviews: A meta-epidemiological study. Osteoarthritis and Cartilage. 2016;24:S42-S3. | Protocol |
