# Supplementary material for: Trial-level characteristics associate with treatment effect estimates: a systematic review of meta-epidemiological studies
Source: BMC Med Res Methodol. 2022 Jun 15;22:171. doi: 10.1186/s12874-022-01650-5 (PMC9202161; doi:10.1186/s12874-022-01650-5)
Supplement: Supplementary file 6 — Additional file 6: Appendix 6. Number of meta-epidemiological studies on trial-level characteristics related to treatment effect estimates published by year. [file 12874_2022_1650_MOESM6_ESM.docx]

**Appendix 6 Number of meta-epidemiological studies on trial-level characteristics related to treatment effect estimates published by year**
