# Supplementary material for: Trial-level characteristics associate with treatment effect estimates: a systematic review of meta-epidemiological studies
Source: BMC Med Res Methodol. 2022 Jun 15;22:171. doi: 10.1186/s12874-022-01650-5 (PMC9202161; doi:10.1186/s12874-022-01650-5)
Supplement: Supplementary file 7 — Additional file 7: Appendix 7. Main characteristics of 80 meta-epidemiological (ME) studies. [file 12874_2022_1650_MOESM7_ESM.docx]

**Appendix 7 Main characteristics of 80 meta-epidemiological (ME) studies**

| **First author** | **Year of publication** | **Journal** | **Trial-level characteristics evaluated** | **Detailed medical conditions** | **No. of MA** | **No. of RCTs** | **Type of outcome measure** |
| --- | --- | --- | --- | --- | --- | --- | --- |
| Schulz, K. F. | 1995 | JAMA | - Allocation concealment - Sequence generation - Double blinding - Exclusion of participants | Pregnancy and Childbirth | 33 | 250 | Binary |
| Khan, K. S. | 1996 | Fertility Sterility | - Allocation concealment - Sequence generation - Double blinding - Drop out - Parallel group vs cross-over | Infertile patients | NR | 34 | Binary |
| Moher, D. | 1999 | Health Technology Assessment / THE LANCET | - Allocation concealment - Sequence generation - Double blinding | Various areas | 11 | 127 | Binary |
| McAuley, L. | 2000 | THE LANCET | - Publication status (published trial vs gray literature) | Various areas | 41 | 467 | Binary |
| Moher, D. | 2000 | Journal of Clinical Epidemiology | - Language (English language vs language other than English) | Various areas | 19 | NR | Binary |
| Kjaergard, L. L. | 2001 | Annal of Internal Medicine | - Allocation concealment - Sequence generation - Double blinding - Drop out | Various areas | 14 | 190 | Binary |
| Balk, E. M. | 2002 | JAMA | - Allocation concealment - Sequence generation - Double blinding - Study question well defined in introduction or methods - Placebo-control - Multicenter vs single-center - US vs other country - Adequate selection criteria reported - Randomization methodology criteria reported - Blinding of participants - Blinding of personnel - Blinding of outcome assessors - Valid statistical methods - Statistician involvement - Intention to treat analysis - Power calculation reported - Baseline characteristics reported - Baseline imbalance - Confounders accounted for - Dropouts reported - Reason for dropouts given - Percentage of dropouts - Findings support conclusion | Various areas | 26 | 276 | Binary |
| Jüni, P. | 2002 | International Journal of Epidemiology | - Language (English language vs language other than English) | Various areas | 50 | 600 | Binary |
| Egger, M. | 2003 | Health Technology Assessment | - Allocation concealment - Double blinding - Publication status (Published trial vs Grey literature) - Language (English language vs language other than English) - Medline-indexed | NR | 159 | 1635 | Binary |
| Hopewell, S. | 2007 | Cochrane Database of Systematic Reviews | - Publication status (Published trial vs Grey literature) | Various areas | 139 | 1748 | Binary |
| Sampson, M. | 2003 | Journal of Clinical Epidemiology | - Medline-indexed | Various areas | 61 | NR | Binary |
| Contopoulos-Ioannidis, D. G. | 2005 | The American Journal of Psychiatry | - Sample size | Mental health-related | 16 | 133 | Both binary and continuous |
| Pham, B. | 2005 | Journal of Clinical Epidemiology | - Language (English language vs language other than English) | NR | 42 | 662 | Binary |
| Tierney, J. F. | 2005 | International Journal of Epidemiology | - Exclusion of participants | Cancer | 14 | 133 | Time-to-event |
| Lathyris, D. N. | 2007 | International Journal of Epidemiology | - Parallel group vs cross-over | Various areas | 28 | 269 | Both binary and continuous |
| Pildal, J. | 2007 | International Journal of Epidemiology | - Allocation concealment - Double blinding | NR | 70 | 499 | Binary |
| Siersma, V. | 2007 | Statistics in Medicine | - Allocation concealment - Sequence generation - Double blinding - Intention to treat analysis - Power calculation adequate | NR | 48 | 495 | Binary |
| Fenwick, J. | 2008 | Journal of Clinical Periodontology | - Allocation concealment - Blinding of outcome assessors | Periodontology | 5 | 34 | Continuous |
| Wood, L. | 2008 | BMJ | - Allocation concealment - Double blinding | Pregnancy and Childbirth | 146 | 1346 | Binary |
| Nüesch, E. | 2009 | Arthritis and Rheumatism | - Allocation concealment - Blinding of participants | Osteoarthritis | 21 | NR | Continuous |
| Nüesch, E. | 2009 | BMJ | - Exclusion of participants | Osteoarthritis of the hip or knee | 14 | 167 | Continuous |
| Patsopoulos, N. A. | 2009 | Open Medicine | - Trial publication date | NR | 157 | 1149 | Both binary and continuous |
| van Tulder, M. W. | 2009 | Spine | - Allocation concealment - Sequence generation - Drop out - Blinding of participants - Blinding of personnel - Blinding of outcome assessors - Intention to treat analysis - Baseline imbalance - Co-intervention - Compliance - Similar timing of the outcome assessment | Backpain | NR | 216 | Continuous |
| Bassler, D. | 2010 | JAMA | - Early stopping | Various areas | NR | 515 | Binary |
| Contopoulos-Ioannidis, D. G. | 2010 | The Journal of Pediatrics | - Adult RCT vs children RCT | Various areas | 128 | 1394 | Binary |
| Nüesch, E. | 2010 | BMJ | - Sample size | Osteoarthritis | 13 | 153 | Continuous |
| Dechartres, A. | 2011 | Annals of Internal Medicine | - Multicenter vs single-center | Various areas | 48 | 421 | Binary |
| Herbison, P. | 2011 | Journal of Clinical Epidemiology | - Allocation concealment | NR | 67 | NR | Binary |
| Siontis, K. C. | 2011 | International Journal of Epidemiology | - Publication in high-impact general medical journal vs other journals | Various areas | 79 | 1043 | Binary |
| Bafeta, A. | 2012 | BMJ | - Multicenter vs single-center | Various areas | 26 | 292 | Continuous |
| Hempel, S. | 2012 | Agency for Healthcare Research and Quality (US) | - Allocation concealment - Sequence generation - Drop out - Blinding of participants - Blinding of personnel - Blinding of outcome assessors - Intention to treat analysis - Baseline imbalance - Co-intervention - Compliance - Similar timing of the outcome assessment | Various areas | 48 | 630 | Both binary and continuous |
| Hróbjartsson, A. | 2012 | BMJ | - Blinding of outcome assessors | Various areas | NR | 21 | Binary |
| Mhaskar, R. | 2012 | Journal of Clinical Epidemiology | - Allocation concealment - Sequence generation - Intention to treat analysis - Description of blinding procedure - Description of dropouts - Prespecification of alpha error - Prespecification of beta error | Cancer | NR | 429 | Time-to-event |
| Savović, J. | 2012 | Health Technology Assessment | - Allocation concealment - Sequence generation - Double blinding - Drop out - Multicenter vs single-center | Various areas | 234 | 1973 | Both binary and continuous |
| Chaimani, A. | 2013 | International Journal of Epidemiology | - Allocation concealment - Sequence generation - Blinding of participants - Blinding of outcome assessors - Sample size | Various areas | 32 | 613 | Both binary, continuous and time-to-event |
| Dechartres, A. | 2013 | BMJ | - Sample size | Various areas | 93 | 735 | Binary |
| Hróbjartsson, A. | 2013 | Canadian Medical Association Journal | - Blinding of outcome assessors | Various areas | NR | 24 | Continuous |
| Panagiotou, O. A. | 2013 | BMJ | - Less developed vs more developed countries | Various areas | 139 | 1297 | Binary |
| Seegers, V. | 2013 | PLOS ONE | - Elderly RCT vs adult RCT | Various areas | 55 | 524 | Binary |
| Turner, R. M. | 2013 | PLOS ONE | - Underpowered: power <50% vs adequately powered | Various areas | 14886 | NR | Binary |
| Unverzagt, S. | 2013 | Journal of Clinical Epidemiology | - Allocation concealment - Sequence generation - Double blinding - Multicenter vs single-center - Baseline imbalance - Early stopping - Incomplete outcome data - Selective outcome reporting - Preintervention - Crossover - Sufficient follow-up - Competing interest | Various areas | 12 | 82 | Binary |
| Zhang, Z. | 2013 | Critical Care | - Sample size | Various areas | 27 | 317 | Binary |
| Saquib. | 2013 | BMJ | - Primary outcome adjustment vs primary outcome unadjustment | Various areas | NA | 199 | Both binary and continuous |
| Bialy, L. | 2014 | Evidence-based Child Health | - Allocation concealment - Sequence generation - Blinding of participants - Blinding of personnel - Blinding of outcome assessors - Incomplete outcome data - Selective outcome reporting - Industry funding - Other sources of bias - Overall risk of bias | Neonate | NR | 208 | Binary |
| Dechartres, A. | 2014 | JAMA | - Allocation concealment - Sequence generation - Double blinding - Incomplete outcome data - Overall risk of bias | Various areas | 163 | 1240 | Binary |
| Hartling, L. | 2014 | PLOS ONE | - Allocation concealment - Sequence generation - Double blinding - Blinding of outcome assessors - Baseline imbalance - Early stopping - Incomplete outcome data - Selective outcome reporting - Industry funding - Other sources of bias | Child-relevant | 17 | 287 | Both binary and continuous |
| Hróbjartsson, A. | 2014 | International Journal of Epidemiology | - Blinding of participants | Various areas | NR | 12 | Continuous |
| Hróbjartsson, A. | 2014 | International Journal of Epidemiology | - Blinding of outcome assessors | Various areas | NR | 18 | Time-to-event |
| Lathyris, D. | 2014 | Pediatrics | - Adult RCT vs children RCT | Various areas | 176 | 853 | Binary |
| Papageorgiou, S. N. | 2014 | Journal of Clinical Epidemiology | - Language (English language vs language other than English) - Medline-indexed - Sample size - Equal randomization | Dental and maxillofacial medicine | 281 | 3010 | Binary |
| Smaïl-Faugeron, V. | 2014 | BMC Medical Research Methodology | - Parallel RCT vs split-mouth RCT | Oral health | 34 | 56 | Both binary and continuous |
| Abraha, I. | 2015 | BMJ | - Intention to treat analysis | Various areas | 50 | 322 | Binary |
| Armijo-Olivo, S. | 2015 | BMJ Open | - Allocation concealment - Sequence generation | Various areas | 43 | 393 | Continuous |
| Halladay, C. W. | 2015 | Journal of Clinical Epidemiology | - PubMed indexed vs non-PubMed indexed | NR | NR | NR | Both binary, continuous and time-to-event |
| Papageorgiou, S. N. | 2015 | Journal of Clinical Epidemiology | - Sequence generation | Orthodontic | 25 | 75 | Continuous |
| Dechartres, A. | 2016 | BMC Medicine | - Trial registration | Various areas | 67 | 322 | Binary |
| Gartlehner, G. | 2016 | Journal of Clinical Epidemiology | - First trial vs subsequent trial | NR | 100 | NR | Both binary and continuous |
| Koletsi, D. | 2016 | European Journal of Orthodontics | - Allocation concealment - Sequence generation - Double blinding - Blinding of outcome assessors - Incomplete outcome data - Selective outcome reporting - Other sources of bias | Orthodontic | NR | 101 | Both binary and continuous |
| Ndounga Diakou, L. A. | 2016 | Cochrane Database of Systematic Reviews | - Adjudication committee vs on-site assessment | Various areas | NR | 47 | Binary |
| Page, M. J. | 2016 | PLOS ONE | - Allocation concealment - Sequence generation - Double blinding - Blinding of participants - Blinding of personnel - Blinding of outcome assessors - Baseline imbalance - Selective outcome reporting | Various areas | 887 | 8042 | Both binary and continuous |
| Armijo-Olivo, S. | 2017 | American Journal of Physical Medicine and Rehabilitation | - Blinding of participants - Blinding of personnel - Blinding of outcome assessors - Blinding of data analyst - Overall Adequacy of Blinding | Various areas | 43 | 393 | Continuous |
| Alahdab, F. | 2018 | Mayo Clinic Proceedings | - First trial vs subsequent trial | Various areas | 70 | 930 | Binary |
| Bolvig, J. | 2018 | Journal of Clinical Epidemiology | - Allocation concealment - Sequence generation - Multicenter vs single-center - Blinding of participants - Blinding of personnel - Sample size - Incomplete outcome data - Industry funding | Osteoarthritis | 20 | 126 | Continuous |
| Dechartres, A. | 2018 | Annals of Internal Medicine | - Language (English language vs language other than English) - Medline-indexed | Various areas | 698 | 5659 | Binary |
| Janiaud, P. | 2018 | Intensive Care Medicine | - Industry funding | Various areas | NR | 568 | Binary |
| Papageorgiou, S. N. | 2018 | Journal of Clinical Epidemiology | - Trial registration | Orthodontic | 16 | 48 | Continuous |
| Saltaji, H. | 2018 | BMC Medical Research Methodology | - Double blinding - Blinding of participants - Blinding of personnel - Blinding of outcome assessors - Method of blinding (appropriate vs inappropriate or unclear) | Oral health | 64 | 540 | Continuous |
| Saltaji, H. | 2018 | Journal of Dental Research | - Allocation concealment - Sequence generation - Baseline imbalance | Oral health | 64 | 540 | Continuous |
| Savovic, J. | 2018 | American Journal of Epidemiology | - Allocation concealment - Sequence generation - Double blinding - Incomplete outcome data | Various areas | 228 | 2428 | Binary |
| de Almeida, M. O. | 2019 | Archives of Physical Medicine and Rehabilitation | - Allocation concealment - Intention to treat analysis | Low back pain | NR | 128 | Continuous |
| Hulshof, T. A. | 2019 | International Journal of Methods in Psychiatric Research | - Baseline imbalance | Dementia | NR | 23 | Both binary and continuous |
| Leyrat, C. | 2019 | International Journal of Epidemiology | - Individual RCT vs cluster RCT | Various areas | 121 | 1458 | Both binary and continuous |
| Mathes, T. | 2019 | Journal of Clinical Epidemiology | - Registry-based RCT (RRCTs) vs conventional RCT (CRCTs) | Various areas | 29 | 451 | Binary |
| Armijo-Olivo, S. | 2020 | American Journal of Physical Medicine and Rehabilitation | - Blinding of participants - Blinding of outcome assessors | Various areas | 79 | 857 | Continuous |
| Berthelsen, D. B. | 2020 | Journal of Clinical Epidemiology | - Outcomes measured in other ways vs patient-reported outcome measures (PROMs) | Various areas | NR | 205 | Both binary and continuous |
| Haring, R. | 2020 | Journal of Clinical Epidemiology | - Allocation concealment - Sequence generation - Double blinding - Multicenter vs single-center - US vs other country - Blinding of outcome assessors - Sample size - Trial publication date - Incomplete outcome data - Industry funding - Overall risk of bias - Competing interest - Trial registration | Various areas | 19 | 132 | Both binary and continuous |
| Moustgaard, H. | 2020 | BMJ | - Blinding of participants - Blinding of personnel - Blinding of outcome assessors | Various areas | 142 | 1153 | Both binary and continuous |
| Nussbaumer-Streit, B. | 2020 | Journal of Clinical Epidemiology | - All language publication vs English language publication | Various areas | 1281 | 2026 | Both binary and continuous |
| Wang, Z. | 2020 | Journal of General Internal Medicine | - Non inferiority trial vs superiority trial | Various areas | 88 | 1114 | Binary |
| Yi, J. | 2020 | Journal of Endodontics | - Allocation concealment - Sequence generation - Double blinding - Blinding of outcome assessors - Incomplete outcome data - Selective outcome reporting - Other sources of bias | Endodontics | NR | 121 | Both binary and continuous |

MA, meta-analysis; RCT, randomized controlled trial; NR, not reported
