# Supplementary material for: Trial-level characteristics associate with treatment effect estimates: a systematic review of meta-epidemiological studies
Source: BMC Med Res Methodol. 2022 Jun 15;22:171. doi: 10.1186/s12874-022-01650-5 (PMC9202161; doi:10.1186/s12874-022-01650-5)
Supplement: Supplementary file 9 — Additional file 9: Appendix 9. Details on the subgroup analyses in 48 meta-epidemiological (ME) studies. [file 12874_2022_1650_MOESM9_ESM.docx]

**Appendix 9 Details on the subgroup analyses in 48 meta-epidemiological (ME) studies**

| **Subgroup analyses** | **Results (%)**^*^ |
| --- | --- |
| **Based on trial-level characteristics** | |
| Outcome | 30 (62.5) |
| Intervention and/or Comparison | 18 (37.5) |
| Risk of bias | 10 (20.8) |
| Patient/Participant | 8 (16.7) |
| Study design | 4 (8.3) |
| Timeframe | 2 (4.2) |
| Publication related characteristics | 2 (4.2) |
| **Based on meta-analysis-level characteristics** | |
| Effect estimation | 10 (20.8) |
| Heterogeneity between trials in overall meta-analyses | 9 (18.8) |
| Type of review | 5 (10.4) |
| Type of funding | 3 (6.3) |
| Different data analysis methods | 2 (4.2) |
| Number of trials | 1 (2.1) |
| Year of review publication | 1 (2.1) |

*There can be more than one subgroup analysis in each ME study.
