# Supplementary material for: Trial-level characteristics associate with treatment effect estimates: a systematic review of meta-epidemiological studies
Source: BMC Med Res Methodol. 2022 Jun 15;22:171. doi: 10.1186/s12874-022-01650-5 (PMC9202161; doi:10.1186/s12874-022-01650-5)
Supplement: Supplementary file 10 — Additional file 10: Appendix 10. Associations between treatment effect estimates and other trial-level characteristics for binary outcome. [file 12874_2022_1650_MOESM10_ESM.pdf]

Appendix 10 Associations between treatment effect estimates and other trial-level characteristics for binary outcome

Trial−level characteristics	Study	No. of MA (RCTs)*	Ratio of effect size (95%CI)
**Sample size** (number of participants) (larger sample/smaller sample)	Dechartres,2013 (Q4 vs Q3)	93 (735)	0.88 (0.82 to 0.95)
	Dechartres,2013 (Q4 vs Q2)	93 (735)	0.83 (0.75 to 0.91)
	Dechartres,2013 (Q4 vs Q1)	93 (735)	0.68 (0.57 to 0.82)
	Dechartres,2013 (≥ 1000 vs 500−999)	93 (735)	0.90 (0.82 to 1.00)
	Dechartres,2013 (≥ 1000 vs 200−499)	93 (735)	0.81 (0.73 to 0.88)
	Dechartres,2013 (≥ 1000 vs 100−199)	93 (735)	0.70 (0.61 to 0.80)
	Dechartres,2013 (Q2−4 vs Q1)	88 (711)	0.77 (0.65 to 0.91)
	Dechartres,2013 (Q3 and Q4 vs Q1 and Q2)	93 (735)	0.81 (0.74 to 0.88)
	Dechartres,2013 (Q4 vs Q1−3)	92 (722)	0.85 (0.79 to 0.90)
	Dechartres,2013 (≥50 vs <50)	52 (417)	0.56 (0.45 to 0.70)
	Dechartres,2013 (≥200 vs <200)	67 (598)	0.76 (0.68 to 0.85)
	Dechartres,2013 (≥500 vs <500)	43 (434)	0.81 (0.74 to 0.89)
	Dechartres,2013 (≥1000 vs <1000)	28 (301)	0.82 (0.76 to 0.90)
**First trial vs subsequent trials**	Gartlehner,2016	100 (NR)	1.03 (0.98 to 1.08)
**First trial vs overall trials**	Alahdab,2018	70 (930)	2.67 (2.12 to 3.37)
**Overall risk of bias** (Cochrane riks of bias + source of funding) (low vs high or unclear)	Bialy,2014	NR (207)	0.91 (0.71 to 1.15)
**Overall risk of bias** (Cochrane risk of bias) (low vs high or unclear)	Haring,2020	19 (132)	0.79 (0.60 to 1.03)
**Sufficient follow−up** (adequate vs inadequate period of follow up)	Unverzagt,2013	12 (82)	0.90 (0.72 to 1.11)
**Placebo control** (yes vs no)	Balk,2002	26 (276)	0.85 (0.61 to 1.09)
**Statistician involvement** (yes vs no)	Balk,2002	26 (276)	1.04 (0.92 to 1.17)
**Trial location**	US vs country other than US		
	Balk,2002	26 (276)	1.05 (0.93 to 1.19)
	Haring,2020	19 (132)	1.61 (0.92 to 2.82)
	"Research country" vs country other than "Research country"		
	Balk,2002	26 (276)	0.95 (0.70 to 1.29)
	Less developed vs more developed countries		
	Panagiotou,2013	139 (1297)	1.12 (1.06 to 1.18)
**Age of participants**	Children RCT vs adult RCT		
	Contopoulos−Ioannidis,2010	128 (1394)	0.96 (0.86 to 1.08)
	Lathyris,2014	18 (220)	1.03 (0.82 to 1.30)
	Adult RCT vs elderly RCT		
	Seegers,2013	55 (524)	0.91 (0.77 to 1.08)
**Patient−reported outcome measures** (no vs yes)	Berthelsen,2020	NR (205)	1.14 (0.89 to 1.43)
**Parallel RCT vs split−mouth RCT**	Smail−Faugeron,2014	19 (56)	0.96 (0.52 to 1.80)
**Crossover data** (low vs high)	Unverzagt,2013	12 (82)	1.12 (0.76 to 1.64)
**Preintervention** (no vs yes)	Unverzagt,2013	12 (82)	1.16 (1.00 to 1.37)
**Whether a preset sample size was estimated and reached** (adequate vs inadequate)	Siersma,2007	48 (495)	0.95 (0.83 to 1.09)
**Equal vs unequal randomization** (unequal is one of the trial arms was at least twice as large as the other)	Papageorgiou,2014	36 (443)	0.99 (0.91 to 1.08)
**Outcome assessors** (adjudication committee vs on−site assessment)	Diakou LA,2016	NR (47)	1.00 (0.97 to 1.04)
**Trial publication date** (published 2000 or later vs published before 2000)	Haring,2020	19 (132)	1.45 (0.91 to 2.33)
**Prespecification of alpha error** (yes vs no)	Mhaskar,2012	NR (429)	1.00 (0.95 to 1.05)
**Prespecification of beta error** (yes vs no)	Mhaskar,2012	NR (429)	1.00 (0.96 to 1.05)
**Power calculation reported** (yes vs no)	Balk,2002	26 (276)	1.08 (0.95 to 1.23)
**Study question well prespecified** (yes vs no)	Balk,2002	26 (276)	0.85 (0.64 to 1.06)
**Selection criteria reported** (yes vs no)	Balk,2002	26 (276)	0.94 (0.73 to 1.28)
**Whether described method of randomization** (yes vs no)	Balk,2002	26 (276)	1.03 (0.89 to 1.20)
**Whether the statistical methods is valid** (yes vs no)	Balk,2002	26 (276)	1.11 (0.95 to 1.31)
**Confounders accounted for** (yes vs no)	Balk,2002	26 (276)	0.96 (0.79 to 1.23)
**Whether the conclusions is valid** (yes vs no)	Balk,2002	26 (276)	0.83 (0.66 to 1.10)
**Number of dropouts reported** (yes vs no)	Balk,2002	26 (276)	1.26 (0.87 to 2.05)
**Reason for dropouts reported** (yes vs no)	Balk,2002	26 (276)	0.93 (0.77 to 1.13)
**Description of dropouts** (yes vs no)	Mhaskar,2012	NR (429)	1.10 (0.87 to 1.39)
**Percentage of dropouts** (low vs high)	Balk,2002	26 (276)	1.02 (0.94 to 1.12)
**Other sources of bias** (stop early, design−specific features and baseline imbalance) (low vs high or unclear)	Bialy,2014	NR (207)	1.09 (0.88 to 1.35)

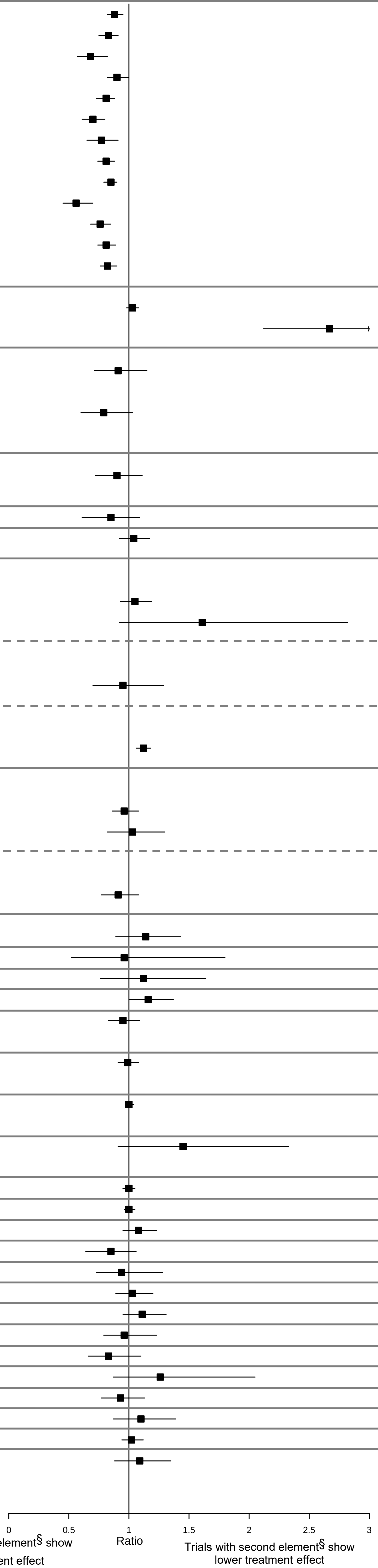

MA, meta-analyses; RCT, randomized controlled trial; ME, meta-epidemiological; NR, not reported; CI, confidence interval

*Values are numbers of MA (RCTs) unless stated otherwise.

#"Research country" includes Australia, Canada, Israel, Japan, New Zealand, United States, and Western Europe.

§For example, sample size (larger sample vs smaller sample), smaller sample is regarded as second element.
