# Supplementary material for: Trial-level characteristics associate with treatment effect estimates: a systematic review of meta-epidemiological studies
Source: BMC Med Res Methodol. 2022 Jun 15;22:171. doi: 10.1186/s12874-022-01650-5 (PMC9202161; doi:10.1186/s12874-022-01650-5)
Supplement: Supplementary file 11 — Additional file 11: Appendix 11. Associations between treatment effect estimates and trial-level characteristics according to different subgroup analyses. [file 12874_2022_1650_MOESM11_ESM.zip › Appendix 11-B-1.pdf]

Appendix 11-B-1

Trial−level characteristics	Subgroup (type of outcome)	No. of MA (RCTs)*	Ratio of effect size (95%CI)
**Sequence generation** (low vs high or unclear)	**Objective outcome**		
	Savovic,2012 (mortality)	16 (129)	0.89 (0.75 to 1.05)
	Savovic,2012 (objective other than mortality)	47 (328)	0.99 (0.84 to 1.16)
	Chaimani,2013 (mortality)	NR (123)	0.98 (0.48 to 1.79)
	Chaimani,2013 (objective other than mortality)	NR (254)	1.16 (0.91 to 1.49)
	Dechartres,2014	59 (NR)	0.83 (0.74 to 0.94)
	Page,2016 (mortality)	3 ME studies (NR)	0.91 (0.78 to 1.06)
	Page,2016 (objective other than mortality)	4 ME studies (NR)	0.98 (0.84 to 1.15)
	Savovic,2018 (mortality)	34 (363)	0.84 (0.71 to 1.01)
	Savovic,2018 (objective other than mortality)	47 (523)	0.99 (0.87 to 1.16)
	Haring,2020	10 (67)	1.13 (0.72 to 1.76)
	**Subjective outcome**		
	Savovic,2012	49 (487)	0.83 (0.74 to 0.94)
	Dechartres,2014	69 (NR)	0.88 (0.78 to 1.01)
	Page,2016	4 ME studies (NR)	0.90 (0.80 to 1.01)
	Savovic,2018	108 (1272)	0.90 (0.83 to 0.98)
	Haring,2020	7 (57)	0.89 (0.55 to 1.43)
**Incomplete outcome data** (low vs high or unclear)	**Objective outcome**		
	Dechartres,2014	46 (NR)	0.96 (0.87 to 1.05)
	Savovic,2018 (mortality)	29 (303)	0.92 (0.79 to 1.08)
	Savovic,2018 (objective other than mortality)	43 (471)	1.03 (0.90 to 1.19)
	**Subjective outcome**		
	Dechartres,2014	58 (NR)	1.06 (0.93 to 1.19)
	Savovic,2018	95 (1182)	0.97 (0.88 to 1.07)
**Trial size** (≥50 vs <50)	**Objective outcome**		
	Haring,2020	7 (52)	0.87 (0.52 to 1.45)
	**Subjective outcome**		
	Haring,2020	6 (52)	0.87 (0.61 to 1.23)
**Multicenter vs single center**	**Objective outcome**		
	Savovic,2012 (mortality)	11 (115)	0.90 (0.69 to 1.17)
	Savovic,2012 (objective other than mortality)	13 (87)	1.05 (0.72 to 1.48)
	Haring,2020	8 (56)	0.94 (0.51 to 1.74)
	**Subjective outcome**		
	Savovic,2012	30 (297)	0.86 (0.68 to 1.05)
	Haring,2020	5 (47)	0.89 (0.59 to 1.33)
**Children RCT vs adult RCT**	**Mortality**		
	Contopoulos−Ioannidis,2010	19 (NR)	0.73 (0.55 to 0.98)
	Lathyris,2014	23 (406)	0.88 (0.70 to 1.11)
	**Outcome other than mortality**		
	Contopoulos−Ioannidis,2010	109 (NR)	1.01 (0.90 to 1.14)
**Adut RCT vs elderly RCT**	**Mortality**		
	Seegers,2013	19 (NR)	1.11 (0.90 to 1.36)
	**Outcome other than mortality**		
	Seegers,2013	36 (NR)	0.86 (0.66 to 1.13)
**Drop out** (no vs yes)	**Mortality**		
	Savovic,2012	9 (80)	1.07 (0.80 to 1.42)
	**Objective outcome other than mortality**		
	Savovic,2012	5 (77)	1.35 (0.63 to 2.94)
	**Subjective outcome**		
	Savovic,2012	13 (207)	1.03 (0.79 to 1.36)
**Individual RCT vs cluster RCT**	**Objective outcome**		
	Leyrat,2019	25 (NR)	1.00 (0.93 to 1.08)
	**Subjective outcome**		
	Leyrat,2019	51 (NR)	0.99 (0.89 to 1.10)

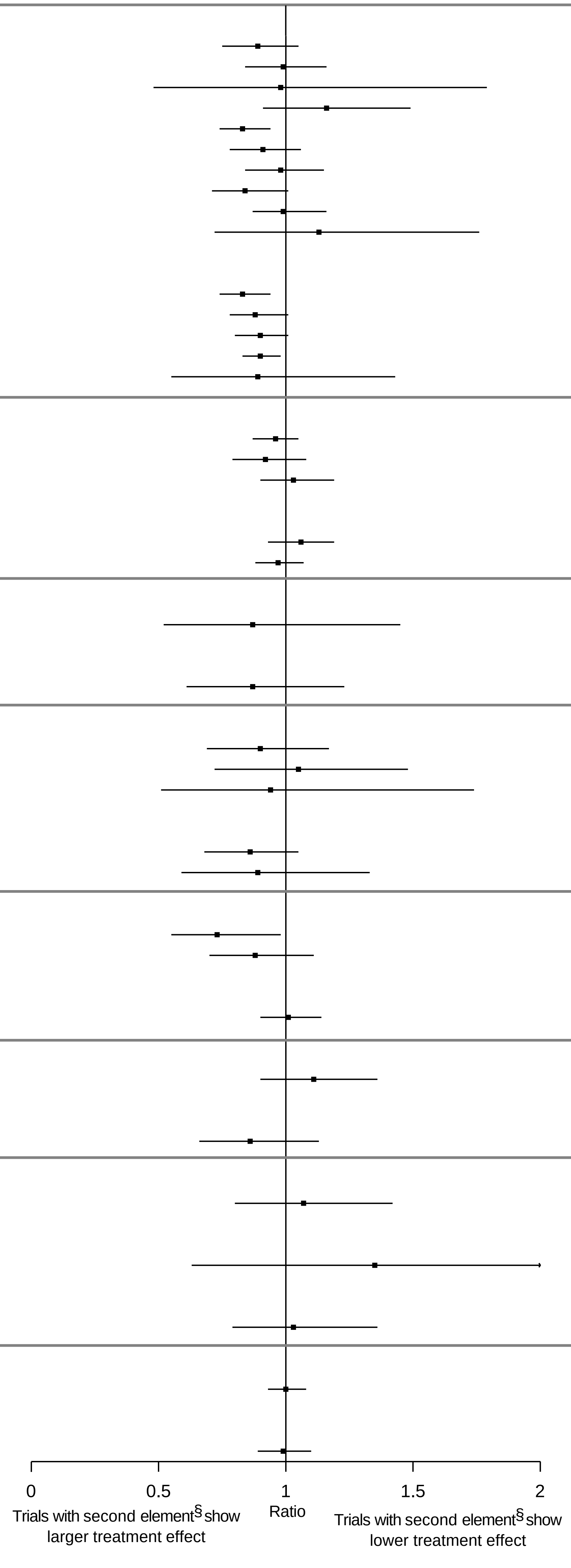

MA, meta-analyses; RCT, randomized controlled trial; ME, meta-epidemiological; NR, not reported; CI, confidence interval
*Values are numbers of MA (RCTs) unless stated otherwise.
§For example, trial size (≥50 vs <50), <50 is regarded as second element.
