# Supplementary material for: Trial-level characteristics associate with treatment effect estimates: a systematic review of meta-epidemiological studies
Source: BMC Med Res Methodol. 2022 Jun 15;22:171. doi: 10.1186/s12874-022-01650-5 (PMC9202161; doi:10.1186/s12874-022-01650-5)
Supplement: Supplementary file 11 — Additional file 11: Appendix 11. Associations between treatment effect estimates and trial-level characteristics according to different subgroup analyses. [file 12874_2022_1650_MOESM11_ESM.zip › Appendix 11-B-2.pdf]

Appendix 11-B-2

Trial−level characteristics	Subgroup (type of intervention)	No. of MA (RCTs)*	Ratio of effect size (95%CI)
**Allocation concealment** (low vs high or unclear)	**Pharmacology intervention**		
	Egger,2003	32 (255)	0.79 (0.69 to 0.91)
	Wood,2008	65 (616)	0.87 (0.76 to 1.00)
	**Non−pharmacology intervention**		
	Egger,2003	7 (49)	0.79 (0.62 to 1.00)
	Wood,2008	37 (188)	0.77 (0.64 to 0.93)
**Double blinding**# (low vs high or unclear)	**Pharmacology intervention**		
	Egger,2003	42 (379)	0.87 (0.74 to 1.03)
	Wood,2008	57 (622)	0.92 (0.81 to 1.05)
	**Non−pharmacology intervention**		
	Egger,2003	3 (20)	1.37 (0.53 to 3.51)
	Wood,2008	19 (124)	1.00 (0.71 to 1.39)
**Language** (English language vs Language other than English)	**Pharmacology intervention**		
	Juni,2002	40 (514)	0.82 (0.72 to 0.93)
	**Non-pharmacology intervention**		
	Juni,2002	10 (86)	0.89 (0.63 to 1.27)
**Multicenter vs single center**	**Pharmacology intervention**		
	Dechartres,2011	28 (240)	0.80 (0.69 to 0.93)
	**Non−pharmacology intervention**		
	Dechartres,2011	20 (181)	0.66 (0.53 to 0.82)
**Published trials vs grey literature**	**Pharmacology intervention**		
	Egger,2003	49 (710)	1.08 (0.97 to 1.20)
	**Non−pharmacology intervention**		
	Egger,2003	11 (73)	1.04 (0.87 to 1.23)
**Individual RCT vs cluster RCT**	**Pharmacology intervention**		
	Leyrat,2019	25 (NR)	1.02 (0.94 to 1.10)
	**Non−pharmacology intervention**		
	Leyrat,2019	51 (NR)	0.98 (0.89 to 1.08)

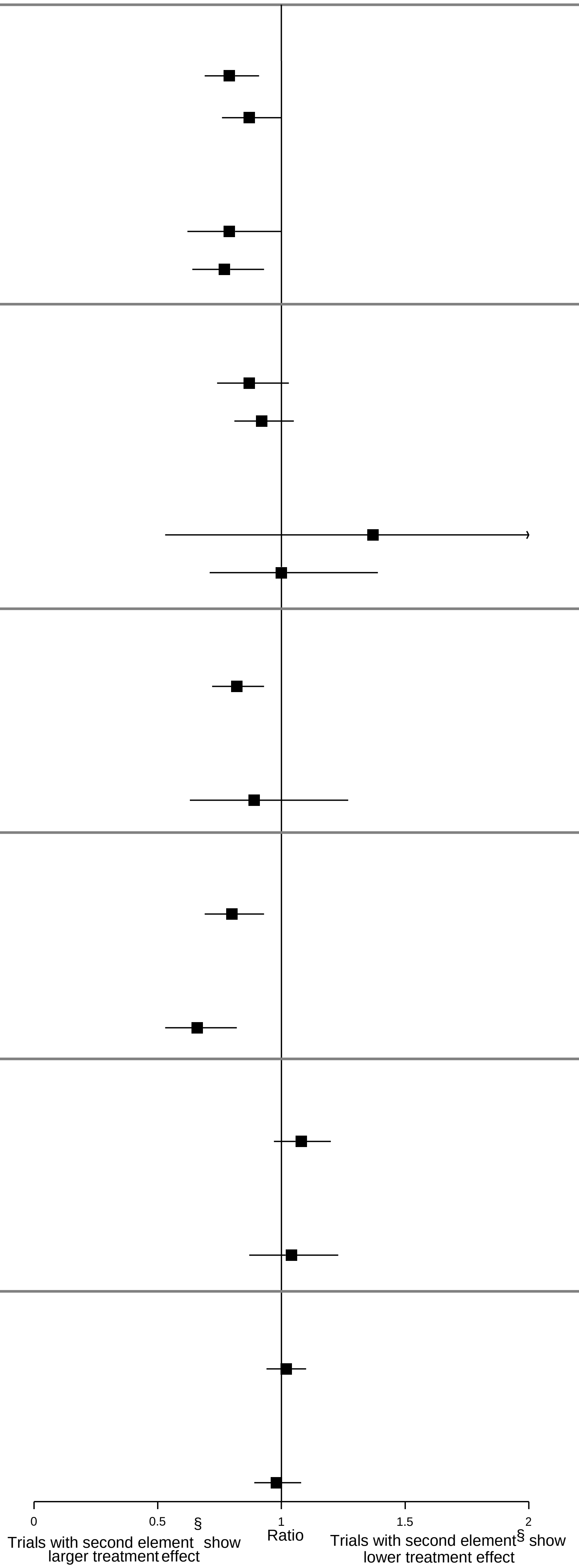

MA, meta-analyses; RCT, randomizedcontrolled trial; NR, not reported; CI, confidence interval

*Values are numbers of MA (RCTs) unless stated otherwise.

#Described as double-blinding or ≥2 key groups (participants, personnel, outcome assessors) were blinded.

§For example, Medline vs non-Medline, non-Medline is regarded as second element.
