# Supplementary material for: Trial-level characteristics associate with treatment effect estimates: a systematic review of meta-epidemiological studies
Source: BMC Med Res Methodol. 2022 Jun 15;22:171. doi: 10.1186/s12874-022-01650-5 (PMC9202161; doi:10.1186/s12874-022-01650-5)
Supplement: Supplementary file 11 — Additional file 11: Appendix 11. Associations between treatment effect estimates and trial-level characteristics according to different subgroup analyses. [file 12874_2022_1650_MOESM11_ESM.zip › Appendix 11-B-3.pdf]

Appendix 11-B-3

Trial−level characteristics	Subgroup (type of control)	No. of MA (RCTs)*	Ratio of effect size (95%CI)
**Allocation concealment** (low vs high or unclear)	**Active control** Egger,2003	13 (100)	0.71 (0.56 to 0.90)
	**Inactive control** Egger,2003	26 (204)	0.84 (0.74 to 0.97)
**Double blinding#** (low vs high or unclear)	**Active control** Egger,2003	10 (69)	0.81 (0.43 to 1.52)
	**Inactive control** Egger,2003	35 (330)	0.91 (0.79 to 1.06)
**Language** (English language vs Language other than English)	**Active control** Juni,2002	12 (148)	0.94 (0.72 to 1.23)
	**Inactive control** Juni,2002	38 (452)	0.83 (0.70 to 0.97)
**Medline vs non−Medline**	**Active control** Egger,2003	16 (178)	1.09 (0.81 to 1.46)
	**Inactive control** Egger,2003	50 (563)	0.90 (0.78 to 1.05)
**Published trials vs grey literature**	**Active control** Egger,2003	17 (193)	0.95 (0.80 to 1.14)
	**Inactive control** Egger,2003	43 (590)	1.10 (0.99 to 1.23)
**Adult RCT vs elderly RCT**	**Active control** Seegers,2013	20 (NR)	0.95 (0.82 to 1.10)
	**Inactive control** Seegers,2013	35 (NR)	0.87 (0.68 to 1.11)
**Individual RCT vs cluster RCT**	**Active control** Leyrat,2019	24 (NR)	1.02 (0.89 to 1.15)
	**Inactive control** Leyrat,2019	52 (NR)	1.01 (0.91 to 1.11)

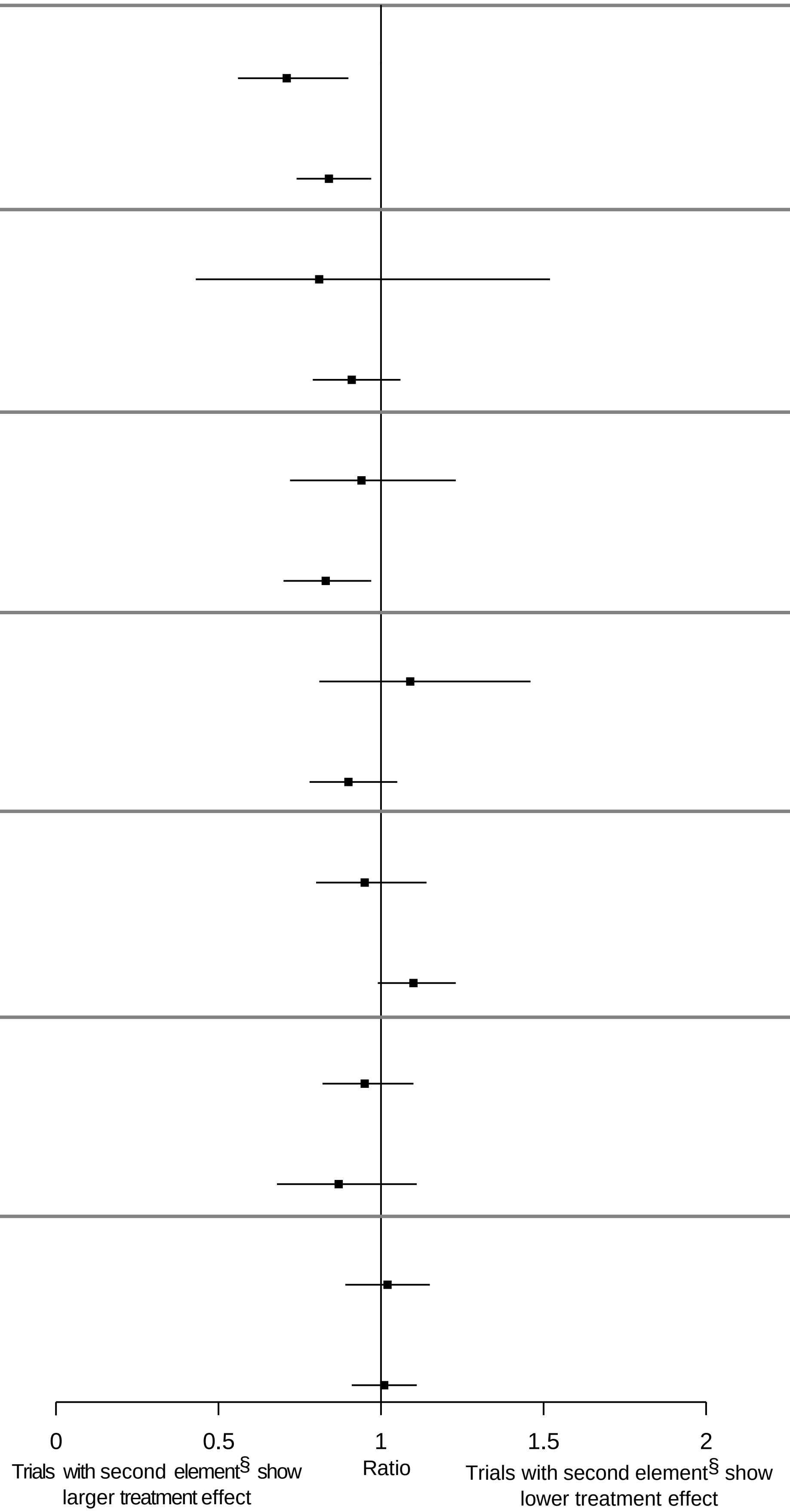

MA, meta-analyses; RCT, randomized controlled trial; NR, not reported; CI, confidence interval
*Values are numbers of MA (RCTs) unless stated otherwise.
#Described as double-blinding or ≥2 key groups (participants, personnel, outcome assessors) were blinded.
§For example, Medline vs non-Medline, non-Medline is regarded as second element.
