# Supplementary material for: Trial-level characteristics associate with treatment effect estimates: a systematic review of meta-epidemiological studies
Source: BMC Med Res Methodol. 2022 Jun 15;22:171. doi: 10.1186/s12874-022-01650-5 (PMC9202161; doi:10.1186/s12874-022-01650-5)
Supplement: Supplementary file 11 — Additional file 11: Appendix 11. Associations between treatment effect estimates and trial-level characteristics according to different subgroup analyses. [file 12874_2022_1650_MOESM11_ESM.zip › Appendix 11-C-1.pdf]

Appendix 11-C-1

Trial−level characteristics	Subgroup (type of outcome)	No. of MA (RCTs)*	Difference of effect size (95%CI)
**Sequence generation** (low vs high or unclear)	**Objective outcome**		
	Hartling,2014	11 (178)	−0.08 (−0.71 to 0.43)
	Armijo−Olivo,2015	NR	0.06 (−0.20 to 0.31)
	Papageorgiou,2015	24 (NR)	−0.01 (−0.28 to 0.26)
	Saltaji,2018 (a)	NR	−0.03 (−0.34 to 0.28)
	**Subjective outcome**		
	Hartling,2014	6 (109)	−0.07 (−0.32 to 0.17)
	Armijo−Olivo,2015	NR	0.00 (−0.16 to 0.17)
	Papageorgiou,2015	1 (NR)	0.02 (−0.48 to 0.52)
	Saltaji,2018 (a)	NR	−0.15 (−0.28 to −0.01)
**Incomplete outcome data** (low vs high or unclear)	**Objective outcome**		
	Hartling,2014	11 (178)	−0.07 (−0.30 to 0.17)
	**Subjective outcome**		
	Hartling,2014	6 (109)	−0.15 (−0.43 to 0.13)
**Selective outcome reporting** (low vs high or unclear)	**Objective outcome**		
	Hartling,2014	11 (178)	−0.04 (−0.21 to 0.12)
	**Subjective outcome**		
	Hartling,2014	6 (109)	−0.08 (−0.20 to 0.05)
**Individual RCT vs cluster RCT**	**Objective outcome**		
	Leyrat,2019	11 (NR)	0.05 (−0.08 to 0.17)
	**Subjective outcome**		
	Leyrat,2019	34 (NR)	0.15 (0.08 to 0.22)
**Statistical power of studies** (adequately powered vs power <50%)	**All−cause mortality**		
	Turner,2013	NR	−0.08 (−0.16 to −0.00)
	**Semi−objective outcome**		
	Turner,2013	NR	−0.11 (−0.15 to −0.06)
	**Subjective outcome**		
	Turner,2013	NR	−0.11 (−0.14 to −0.08)
**Trial registration** (yes vs no)	**Objective outcome**		
	Papageorgiou,2018	13 (NR)	−0.46 (−0.75 to −0.17)
	**Subjective outcome**		
	Papageorgiou,2018	3 (NR)	−0.14 (−0.56 to 0.29)
**Baseline imbalance** (no vs yes)	**Objective outcome**		
	Hartling,2014	11 (178)	−0.02 (−0.39 to 0.35)
	Saltaji,2018(a)	NR	0.03 (−0.55 to 0.61)
	**Subjective outcome**		
	Hartling,2014	6 (109)	−0.07 (−0.31 to 0.17)
	Saltaji,2018(a)	NR	−0.01 (−0.12 to 0.10)
**Industry funding** (no vs yes)	**Objective outcome**		
	Hartling,2014	11 (178)	0.02 (−0.20 to 0.24)
	**Subjective outcome**		
	Hartling,2014	6 (109)	0.04 (−0.20 to 0.29)
**Other sources of bias** (low vs high or unclear)	**Objective outcome**		
	Hartling,2014	11 (178)	0.11 (−0.10 to 0.32)
	**Subjective outcome**		
	Hartling,2014	6 (109)	−0.08 (−0.32 to 0.16)
**Blinding of principal−investigator** (low vs high or unclear)	**Objective outcome**		
	Saltaji,2018 (b)	NR	−0.07 (−0.36 to 0.22)
	**Subjective outcome**		
	Saltaji,2018 (b)	NR	0.03 (−0.07 to 0.13)

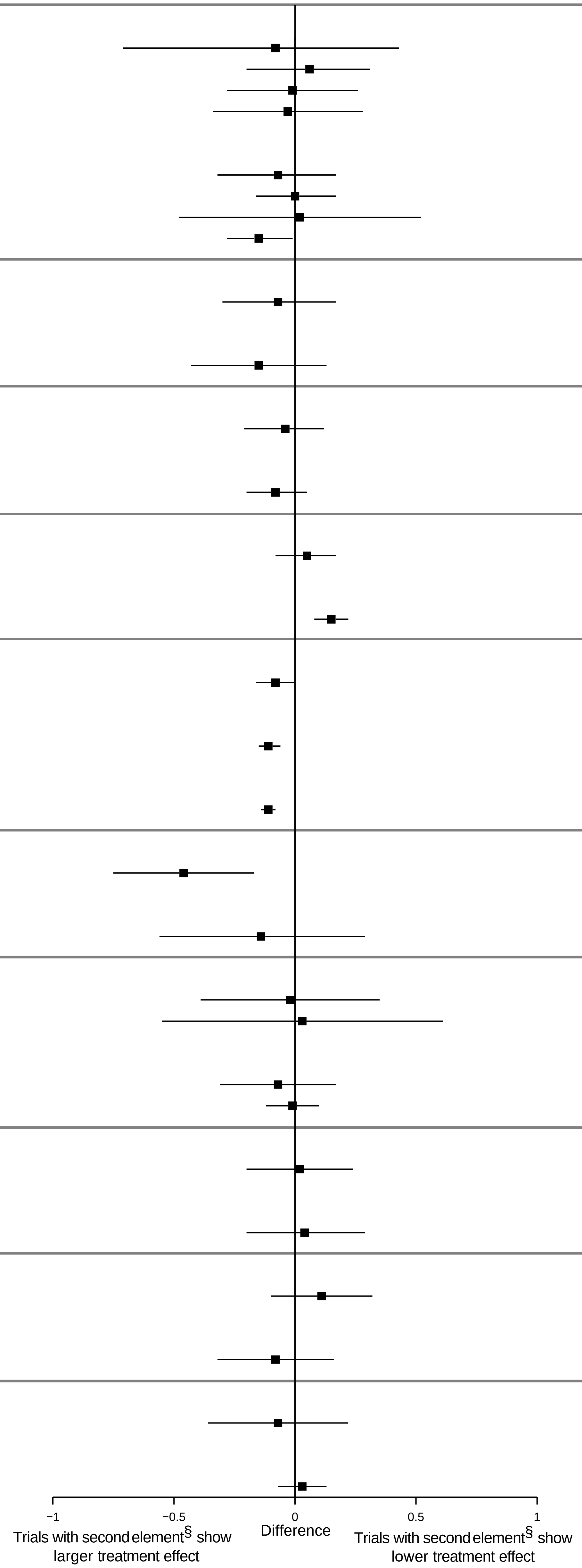

MA, meta-analyses; RCT, randomized controlled trial; NR, not reported; CI, confidence interval

*Values are numbers of MA (RCTs) unless stated otherwise.

§For example, individual RCT vs cluster RCT, cluster RCT is regarded as second element.
