# Supplementary material for: Trial-level characteristics associate with treatment effect estimates: a systematic review of meta-epidemiological studies
Source: BMC Med Res Methodol. 2022 Jun 15;22:171. doi: 10.1186/s12874-022-01650-5 (PMC9202161; doi:10.1186/s12874-022-01650-5)
Supplement: Supplementary file 11 — Additional file 11: Appendix 11. Associations between treatment effect estimates and trial-level characteristics according to different subgroup analyses. [file 12874_2022_1650_MOESM11_ESM.zip › Appendix 11-C-2.pdf]

Appendix 11-C-2

Trial−level characteristics	Subgroup (type of intervention)	No. of MA (RCTs)[*]	Difference of effect size (95%CI)
**Sequence generation** (low vs high or unclear)	**Pharmacology intervention**		
	Hartling,2014	7 (97)	0.07 (−0.14 to 0.29)
	**Non−pharmacology intervention**		
	Hartling,2014	10 (190)	−0.16 (−0.38 to 0.05)
**Allocation concealment** (low vs high or unclear)	**Pharmacology intervention**		
	Nuesch,2009(a)	7 (109)	−0.24 (−0.53 to 0.04)
	Hartling,2014	7 (97)	0.03 (−0.31 to 0.37)
	**Non−pharmacology intervention**		
	Nuesch,2009(a)	7 (49)	−0.05 (−0.22 to 0.12)
	Hartling,2014	10 (190)	0.17 (−0.20 to 0.53)
**Blinding of participants** (low vs high or unclear)	**Pharmacology intervention**		
	Nuesch,2009(a)	6 (96)	0.04 (−0.12 to 0.19)
	Hrobjartsson,2014(a)	NR (1)	−0.12 (−0.44 to 0.19)
	**Non−pharmacology intervention**		
	Nuesch,2009(a)	4 (26)	−0.67 (−1.04 to −0.29)
	Hrobjartsson,2014(a)	NR (11)	−0.60 (−0.75 to −0.46)
**Blinding of outcome assessors** (low vs high or unclear)	**Pharmacology intervention**		
	Hartling,2014	7 (97)	0.15 (−0.10 to 0.41)
	**Non−pharmacology intervention**		
	Hartling,2014	10 (190)	−0.06 (−0.14 to 0.03)
**Double blinding[#]** (low vs high or unclear)	**Pharmacology intervention**		
	Hartling,2014	7 (97)	0.08 (−0.12 to 0.29)
	**Non−pharmacology intervention**		
	Hartling,2014	10 (190)	−0.02 (−0.12 to 0.08)
**Incomplete outcome data** (low vs high or unclear)	**Pharmacology intervention**		
	Hartling,2014	7 (97)	0.02 (−0.20 to 0.23)
	**Non−pharmacology intervention**		
	Hartling,2014	10 (190)	−0.17 (−0.42 to 0.07)
**Selective outcome reporting** (low vs high or unclear)	**Pharmacology intervention**		
	Hartling,2014	7 (97)	−0.13 (−0.35 to 0.10)
	**Non−pharmacology intervention**		
	Hartling,2014	10 (190)	−0.04 (−0.15 to 0.07)
**Sample size** (≥100 vs <100 per arm)	**Pharmacology intervention**		
	Nuesch,2009(b)	8 (113)	−0.23 (−0.39 to −0.08)
	**Non−pharmacology intervention**		
	Nuesch,2009(b)	5 (40)	−0.17 (−0.40 to 0.06)
**Exclusion of participants** (no vs yes)	**Pharmacology intervention**		
	Nuesch,2009(b)	8 (114)	−0.16 (−0.41 to 0.09)
	**Non−pharmacology intervention**		
	Nuesch,2009(b)	6 (53)	−0.05 (−0.17 to 0.07)
**Individual RCT vs cluster RCT**	**Pharmacology intervention**		
	Leyrat,2019	6 (NR)	−0.03 (−0.12 to 0.07)
	**Non−pharmacology intervention**		
	Leyrat,2019	39 (NR)	0.15 (0.08 to 0.21)
**Baseline imbalance** (no vs yes)	**Pharmacology intervention**		
	Hartling,2014	7 (97)	0.23 (−0.37 to 0.83)
	**Non−pharmacology intervention**		
	Hartling,2014	10 (190)	−0.12 (−0.28 to 0.03)
**Industry funding** (no vs yes)	**Pharmacology intervention**		
	Hartling,2014	7 (97)	0.05 (−0.34 to 0.44)
	**Non−pharmacology intervention**		
	Hartling,2014	10 (190)	0.03 (−0.10 to 0.16)
**Multicenter vs single center**	**Pharmacology intervention**		
	Bafeta,2012	16 (119)	−0.11 (−0.29 to 0.08)
	**Non−pharmacology intervention**		
	Bafeta,2012	10 (173)	−0.08 (−0.18 to 0.01)
**Other sources of bias** (low vs high or unclear)	**Pharmacology intervention**		
	Hartling,2014	7 (97)	0.17 (−0.21 to 0.56)
	**Non−pharmacology intervention**		
	Hartling,2014	10 (190)	−0.04 (−0.14 to 0.05)

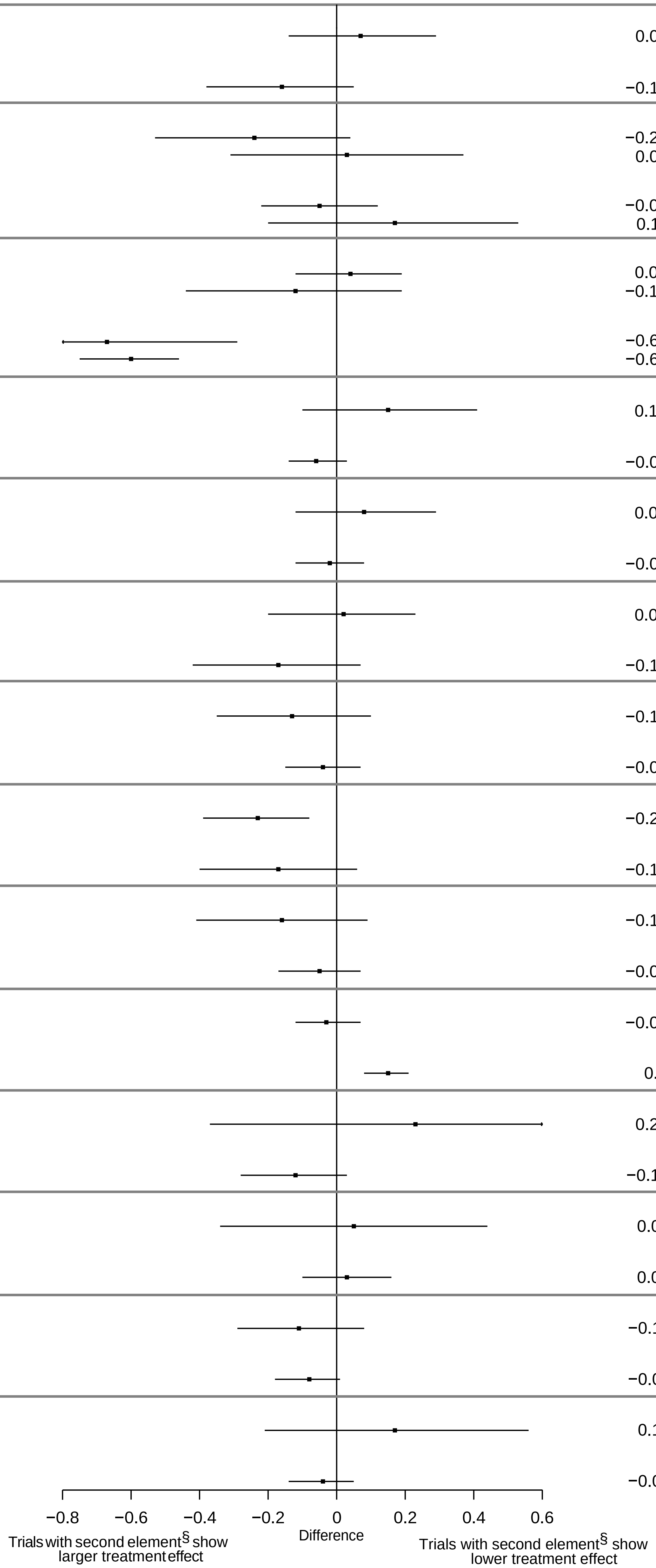

MA, meta-analyses; RCT, randomized controlled trial; NR, not reported; CI, confidence interval
[*]Values are numbers of MA (RCTs) unless stated otherwise.
[#]Described as double-blinding or ≥2 key groups (participants, personnel, outcome assessors) were blinded.
[§]For example, individual RCT vs cluster RCT, cluster RCT is regarded as second element.
