# Supplementary material for: Trial-level characteristics associate with treatment effect estimates: a systematic review of meta-epidemiological studies
Source: BMC Med Res Methodol. 2022 Jun 15;22:171. doi: 10.1186/s12874-022-01650-5 (PMC9202161; doi:10.1186/s12874-022-01650-5)
Supplement: Supplementary file 11 — Additional file 11: Appendix 11. Associations between treatment effect estimates and trial-level characteristics according to different subgroup analyses. [file 12874_2022_1650_MOESM11_ESM.zip › Appendix 11-C-3.pdf]

Appendix 11-C-3

Trial−level characteristics	Subgroup (type of control)	No. of MA (RCTs)*	Difference of effect size (95%CI)
**Sequence generation** (low vs high or unclear)	**Active control**		
	Hartling,2014	8 (159)	−0.37 (−0.82 to −0.05)
	**Inactive control**		
	Hartling,2014	9 (128)	0.03 (−0.09 to 0.15)
**Allocation concealment** (low vs high or unclear)	**Active control**		
	Hartling,2014	8 (159)	1.23 (−1.23 to 3.69)
	**Inactive control**		
	Hartling,2014	9 (128)	0.02 (−0.19 to 0.23)
**Blinding of outcome assessors** (low vs high or unclear)	**Active control**		
	Hartling,2014	8 (159)	0.15 (−0.34 to 0.65)
	**Inactive control**		
	Hartling,2014	9 (128)	−0.03 (−0.12 to 0.06)
**Double blinding#** (low vs high or unclear)	**Active control**		
	Hartling,2014	8 (159)	0.04 (−0.20 to 0.28)
	**Inactive control**		
	Hartling,2014	9 (128)	−0.01 (−0.10 to 0.09)
**Incomplete outcome data** (low vs high or unclear)	**Active control**		
	Hartling,2014	8 (159)	−0.78 (−2.39 to 0.83)
	**Inactive control**		
	Hartling,2014	9 (128)	−0.02 (−0.13 to 0.10)
**Selective outcome reporting** (low vs high or unclear)	**Active control**		
	Hartling,2014	8 (159)	0.10 (−0.20 to 0.39)
	**Inactive control**		
	Hartling,2014	9 (128)	−0.07 (−0.17 to 0.03)
**Individual RCT vs cluster RCT**	**Active control**		
	Leyrat,2019	11 (NR)	0.25 (0.15 to 0.36)
	**Inactive control**		
	Leyrat,2019	34 (NR)	0.08 (0.01 to 0.15)
**Baseline imbalance** (no vs yes)	**Active control**		
	Hartling,2014	8 (159)	−0.27 (−0.82 to 0.28)
	**Inactive control**		
	Hartling,2014	9 (128)	−0.02 (−0.27 to 0.23)
**Industry funding** (no vs yes)	**Active control**		
	Hartling,2014	8 (159)	−0.04 (−0.23 to 0.16)
	**Inactive control**		
	Hartling,2014	9 (128)	0.05 (−0.14 to 0.23)
**Other sources of bias** (low vs high or unclear)	**Active control**		
	Hartling,2014	8 (159)	0.03 (−0.16 to 0.21)
	**Inactive control**		
	Hartling,2014	9 (128)	0.02 (−0.27 to 0.30)

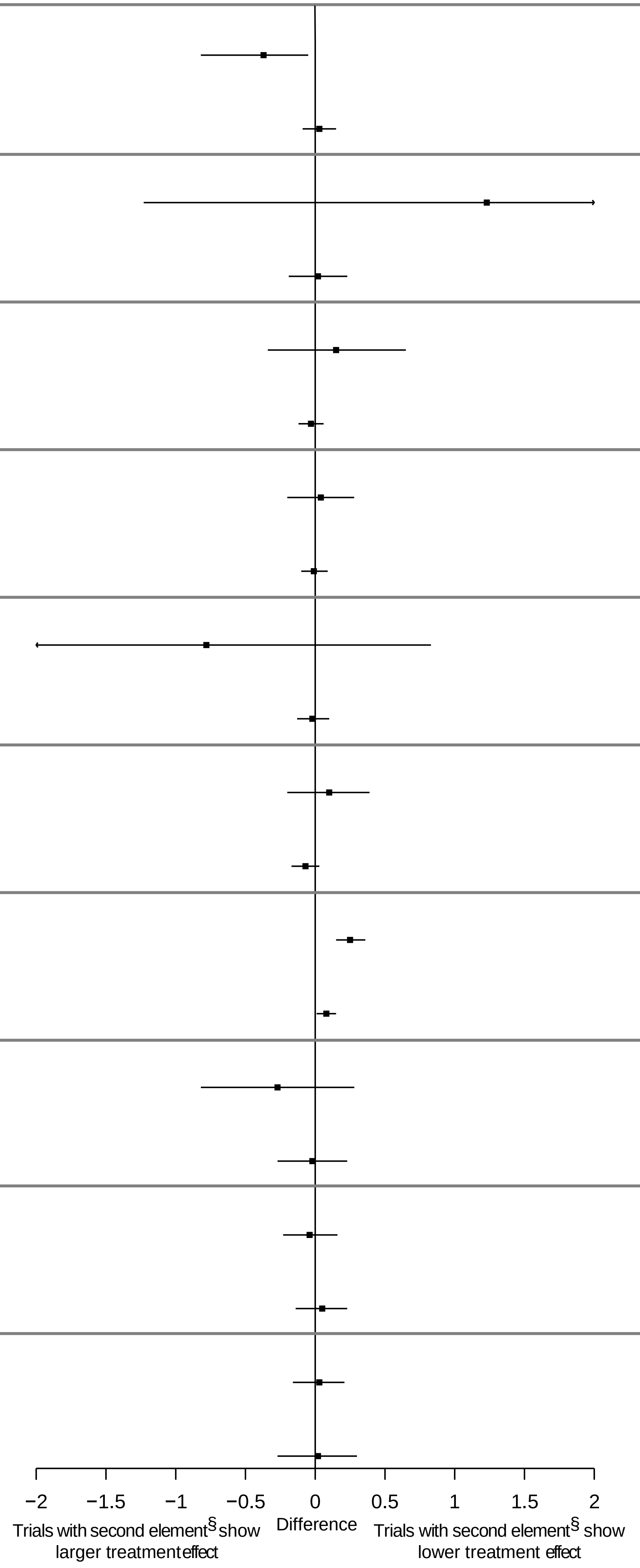

MA, meta-analyses; RCT, randomized controlled trial; NR, not reported; CI, confidence interval
*Values are numbers of MA (RCTs) unless stated otherwise.
#Described as double-blinding or ≥2 key groups (participants, personnel, outcome assessors) were blinded.
§For example, individual RCT vs cluster RCT, cluster RCT is regarded as second element.
