# Supplementary material for: Trial-level characteristics associate with treatment effect estimates: a systematic review of meta-epidemiological studies
Source: BMC Med Res Methodol. 2022 Jun 15;22:171. doi: 10.1186/s12874-022-01650-5 (PMC9202161; doi:10.1186/s12874-022-01650-5)
Supplement: Supplementary file 11 — Additional file 11: Appendix 11. Associations between treatment effect estimates and trial-level characteristics according to different subgroup analyses. [file 12874_2022_1650_MOESM11_ESM.zip › Appendix 11-C-4.pdf]

Appendix 11-C-4

Trial−level characteristics	Subgroup	No. of MA (RCTs)[*]	Difference of effect size (95%CI)
**Allocation concealment** (low vs high or unclear)	**Complementary medicine**		
	Nuesch,2009(a)	5 (50)	−0.52 (−0.93 to −0.10)
	**Non−complementary medicine**		
	Nuesch,2009(a)	9 (108)	−0.01 (−0.10 to 0.07)
**Blinding of participants** (low vs high or unclear)	**Complementary medicine**		
	Nuesch,2009(a)	6 (61)	−0.44 (−0.94 to 0.07)
	Hrobjartsson,2014(a)	NR (12)	−0.56 (−0.71 to −0.41)
	**Non−complementary medicine**		
	Nuesch,2009(a)	4 (61)	0.04 (−0.10 to 0.18)
	**Acupuncture**		
	Hrobjartsson,2014(a)	NR (10)	−0.63 (−0.77 to −0.49)
	**Non−acupuncture**		
	Hrobjartsson,2014(a)	NR (2)	−0.17 (−0.41 to 0.07)
**Exclusion of participants** (no vs yes)	**Complementary medicine**		
	Nuesch,2009(b)	5 (58)	−0.59 (−0.87 to −0.31)
	**Non−complementary medicine**		
	Nuesch,2009(b)	9 (109)	−0.01 (−0.14 to 0.12)

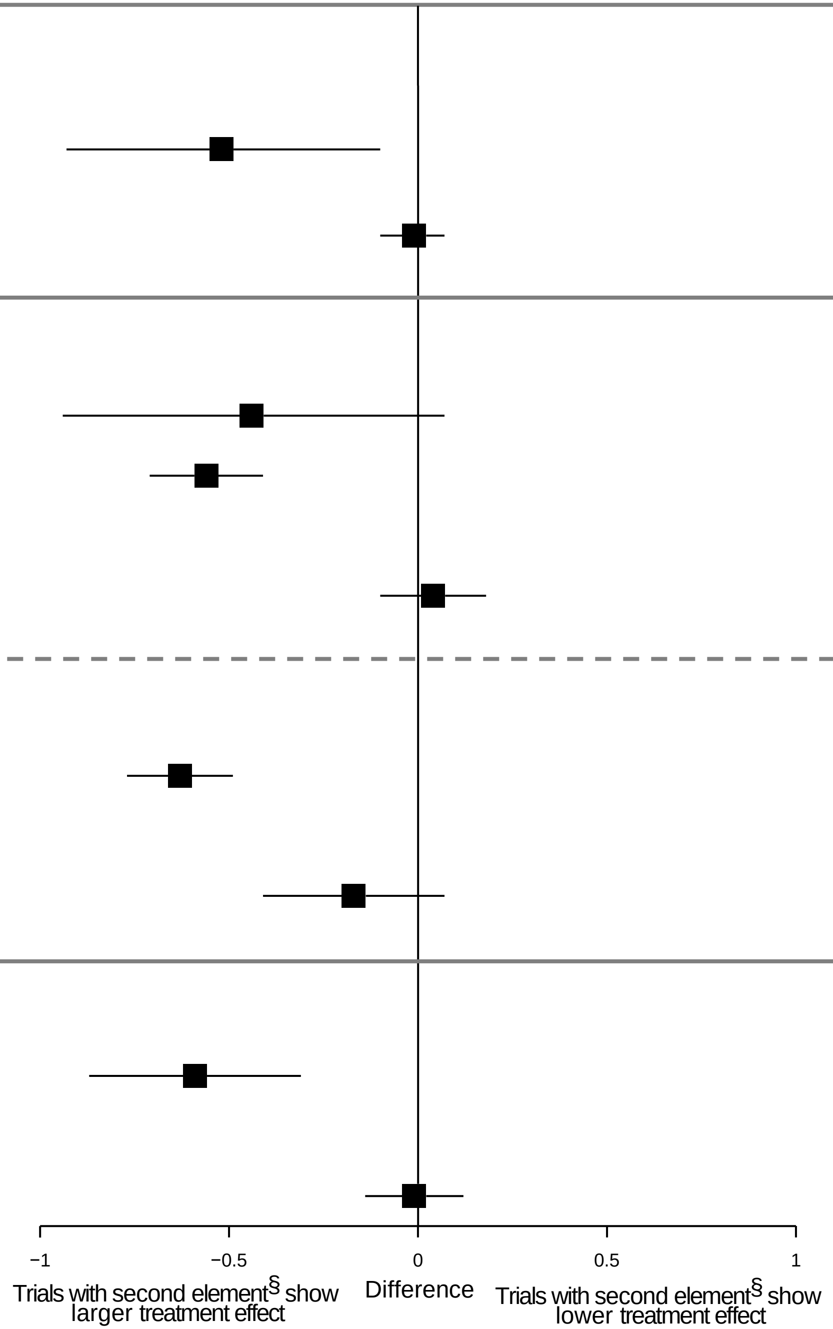

MA, meta-analyses; RCT, randomized controlled trial; NR, not reported; CI, confidence interval
[*]Values are numbers of MA (RCTs) unless stated otherwise.
[§]For example, exclusion of participants(no vs yes), exclusion of participants is regarded as second element.
