# Supplementary material for: Trial-level characteristics associate with treatment effect estimates: a systematic review of meta-epidemiological studies
Source: BMC Med Res Methodol. 2022 Jun 15;22:171. doi: 10.1186/s12874-022-01650-5 (PMC9202161; doi:10.1186/s12874-022-01650-5)
Supplement: Supplementary file 12 — Additional file 12: Appendix 12. Results of additional subgroup analyses. [file 12874_2022_1650_MOESM12_ESM.zip › Appendix 12-B-1.pdf]

**Appendix 12-B-1**

| Trial−level characteristics | Subgroup | No. of MA (RCTs)* | Ratio of effect size (95%CI) |
|---|---|---|---|
| | **Complementary medicine** | | |
| | Juni,2002 | 4 (34) | 0.64 (0.44 to 0.94) |
| | Pham,2005 | 8 (129) | 0.61 (0.38 to 0.97) |
| | **Non−complementary medicine** | | |
| | Juni,2002 | 46 (566) | 0.87 (0.76 to 1.00) |
| | Pham,2005 | 34 (533) | 0.98 (0.79 to 1.20) |
| | **Cochrane review** | | |
| | Juni,2002 | 29 (284) | 0.89 (0.71 to 1.11) |
| **Language** | **Non−Cochrane review** | | |
| (English language vs Language other than English) | Juni,2002 | 21 (316) | 0.81 (0.69 to 0.95) |
| | **English vs Chinese** | | |
| | Dechartres,2018 | 43 (NR) | 0.84 (0.72 to 0.99) |
| | **English vs German** | | |
| | Dechartres,2018 | 32 (NR) | 0.87 (0.68 to 1.10) |
| | **English vs Spanish** | | |
| | Dechartres,2018 | 30 (NR) | 1.00 (0.73 to 1.39) |
| | **English vs French** | | |
| | Dechartres,2018 | 22 (NR) | 0.85 (0.53 to 1.36) |

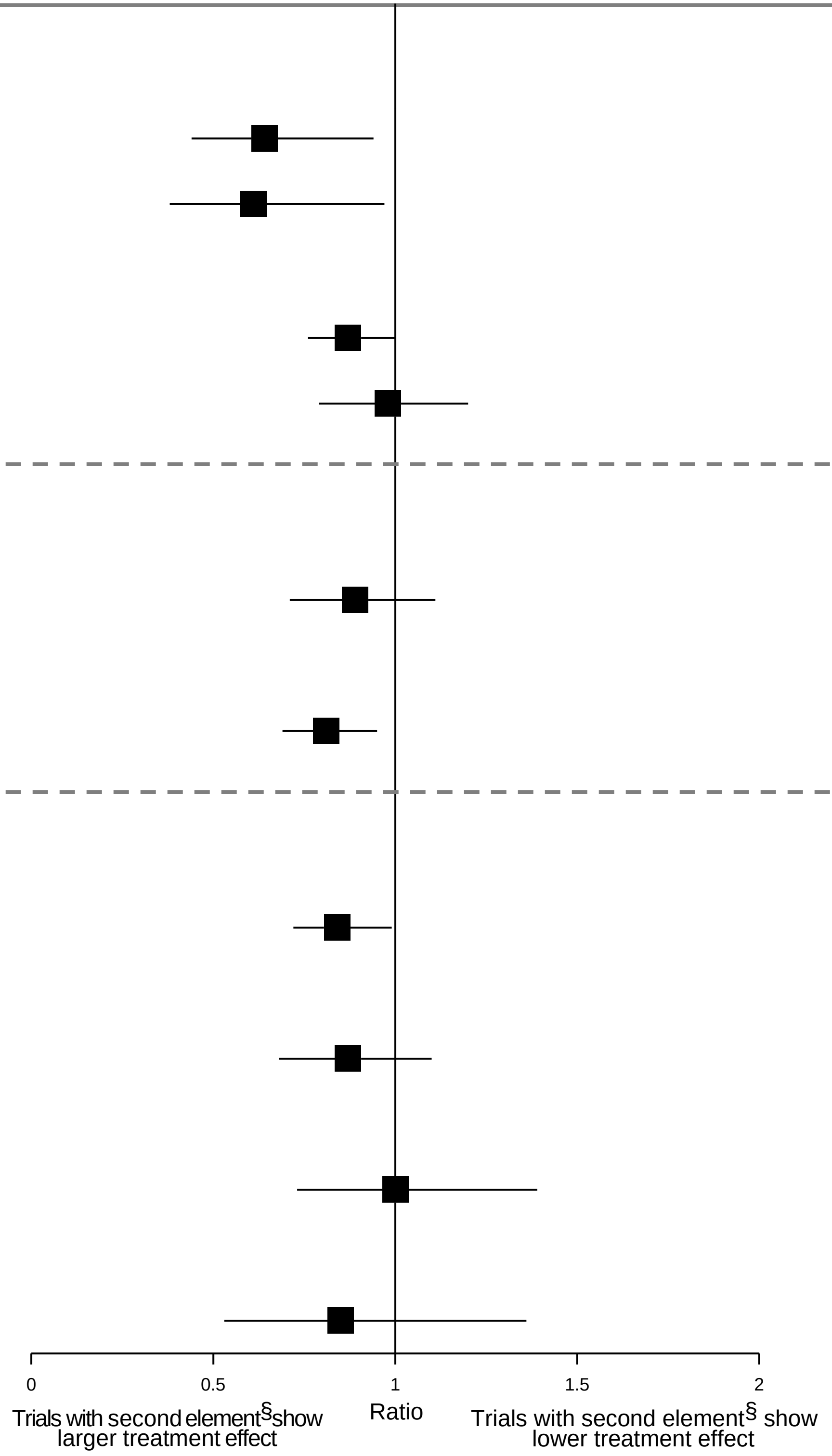


MA, meta-analyses; RCT, randomized controlled trial; NR, not reported; CI, confidence interval
*Values are numbers of MA (RCTs) unless stated otherwise.
§For example, Language (English language vs language other than English), language other than English is regarded as second element.
