# Supplementary material for: Trial-level characteristics associate with treatment effect estimates: a systematic review of meta-epidemiological studies
Source: BMC Med Res Methodol. 2022 Jun 15;22:171. doi: 10.1186/s12874-022-01650-5 (PMC9202161; doi:10.1186/s12874-022-01650-5)
Supplement: Supplementary file 12 — Additional file 12: Appendix 12. Results of additional subgroup analyses. [file 12874_2022_1650_MOESM12_ESM.zip › Appendix 12-B-2.pdf]

Appendix 12-B-2

Trial−level characteristics	Subgroup	No. of MA (RCTs)*	Ratio of effect size (95%CI)
	**Cardiovascular disease**		
	Balk,2002	8 (93)	1.11 (0.87 to 1.39)
	**Infectious disease**		
	Balk,2002	6 (56)	0.84 (0.55 to 1.27)
	**Pediatrics**		
	Balk,2002	5 (60)	1.02 (0.57 to 1.61)
	**Surgery**		
	Balk,2002	7 (67)	0.87 (0.56 to 1.36)
	**Wound/ulcer**		
	Hrobjartsson,2012	NR (5)	0.84 (0.55 to 1.29)
	**Fractured bone**		
	Hrobjartsson,2012	NR (3)	0.63 (0.33 to 1.20)
	Hrobjartsson,2014(b)	NR (3)	0.85 (0.66 to 1.09)
	**Angina pectoris**		
	Hrobjartsson,2012	NR (2)	0.31 (0.13 to 0.71)
	**Facial folds**		
	Hrobjartsson,2012	NR (2)	0.22 (0.00 to 19.8)
	**Multiple sclerosis**		
	Hrobjartsson,2014(b)	NR (1)	0.81 (0.47 to 1.38)
	**CMV retinitis**		
	Hrobjartsson,2014(b)	NR (7)	0.92 (0.59 to 1.42)
	**Paired patient level data (blinded, non−blinded), IPD**		
	Hrobjartsson,2012	NR (8)	0.77 (0.55 to 1.09)
	Hrobjartsson,2014(b)	NR (3)	0.86 (0.75 to 0.98)
	**Paired patient level data (blinded, non−blinded), no IPD**		
	Hrobjartsson,2012	NR (4)	0.38 (0.17 to 0.81)
	Hrobjartsson,2014(b)	NR (7)	0.66 (0.51 to 0.85)
	**Summary outcome (no paired patient level data)**		
	Hrobjartsson,2012	NR (9)	0.57 (0.19 to 1.69)
**Blinding of outcome assessors**	Hrobjartsson,2014(b)	NR (11)	0.85 (0.67 to 1.08)
	**Observer bias main objective**		
	Hrobjartsson,2012	NR (4)	0.84 (0.41 to 1.71)
	Hrobjartsson,2014(b)	NR (1)	0.80 (0.47 to 1.38)
	**Observer bias not main objective**		
	Hrobjartsson,2012	NR (17)	0.59 (0.36 to 0.97)
	Hrobjartsson,2014(b)	NR (6)	0.70 (0.52 to 0.94)
	**Parallel group design**		
	Hrobjartsson,2012	NR (19)	0.74 (0.54 to 1.02)
	**Split−body/cross−over design**		
	Hrobjartsson,2012	NR (2)	0.12 (0.04 to 3.74)
	**Same type of outcome assessors (such as neurologist vs neurologist)**		
	Hrobjartsson,2012	NR (10)	0.50 (0.22 to 1.10)
	Hrobjartsson,2014(b)	NR (2)	0.75 (0.46 to 1.22)
	**Not same type of outcome assessors (such as radiologist vs surgeon)**		
	Hrobjartsson,2012	NR (11)	0.72 (0.48 to 1.07)
	Hrobjartsson,2014(b)	NR (5)	0.70 (0.50 to 0.97)
	**Same type of blinding procedure (such as radiographs vs radiographs)**		
	Hrobjartsson,2012	NR (5)	0.72 (0.27 to 1.92)
	Hrobjartsson,2014(b)	NR (2)	0.72 (0.51 to 1.00)
	**Not same type of blinding procedure (such as photo vs live observation)**		
	Hrobjartsson,2012	NR (16)	0.61 (0.39 to 0.95)
	Hrobjartsson,2014(b)	NR (5)	0.67 (0.45 to 1.02)
	**All patients seen by both assessors**		
	Hrobjartsson,2012	NR (13)	0.70 (0.52 to 0.96)
	**Minority patients seen by only one type of assessor**		
	Hrobjartsson,2012	NR (7)	0.50 (0.15 to 1.76)

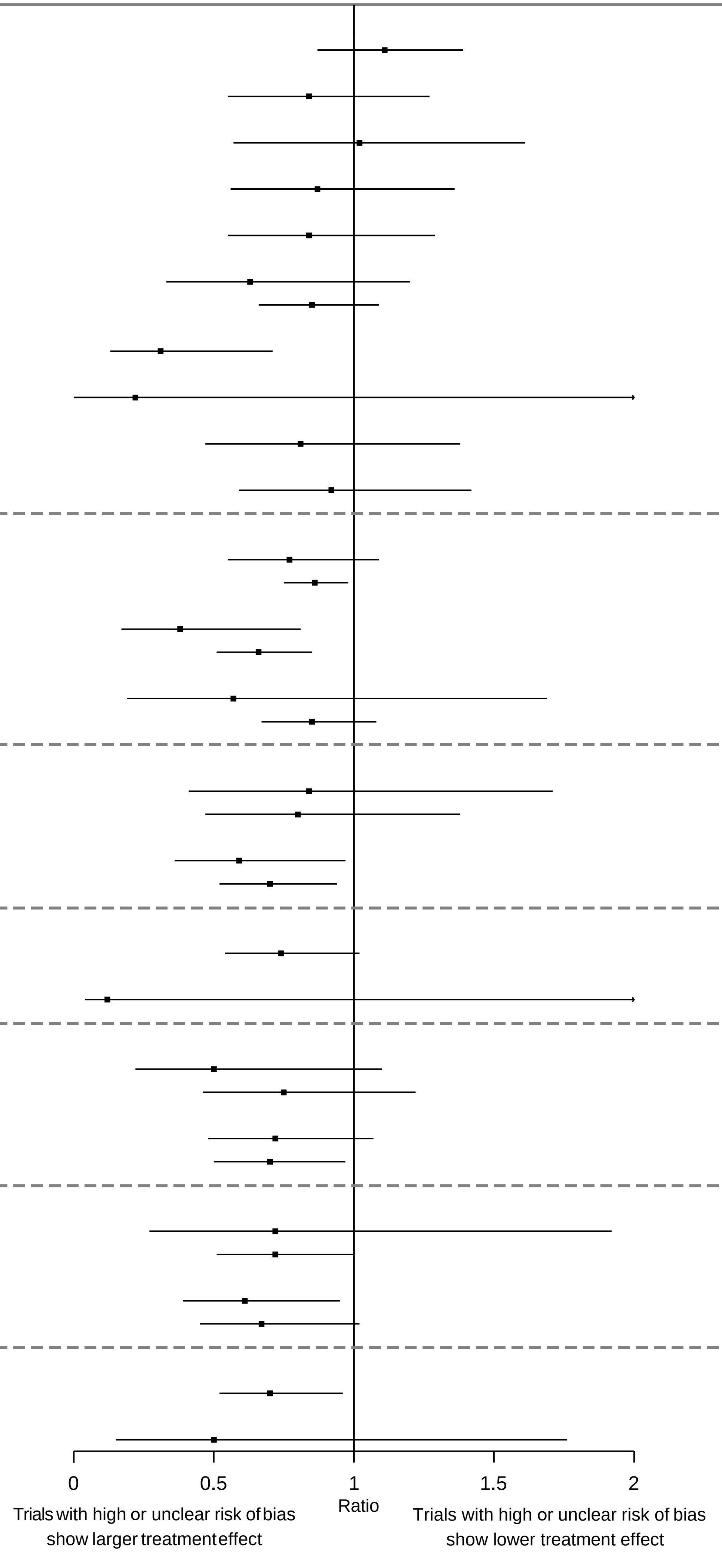

MA, meta-analyses; RCT, randomized controlled trial; CI, confidence interval; NR, not reported
*Values are numbers of MA(RCTs) unless stated otherwise.
