# Supplementary material for: Trial-level characteristics associate with treatment effect estimates: a systematic review of meta-epidemiological studies
Source: BMC Med Res Methodol. 2022 Jun 15;22:171. doi: 10.1186/s12874-022-01650-5 (PMC9202161; doi:10.1186/s12874-022-01650-5)
Supplement: Supplementary file 12 — Additional file 12: Appendix 12. Results of additional subgroup analyses. [file 12874_2022_1650_MOESM12_ESM.zip › Appendix 12-B-3.pdf]

Appendix 12-B-3

Trial−level characteristics	Subgroup	No. of MA (RCTs)*	Ratio of effect size(95%CI)
Sequence generation	**Cardiovascular**		
	Balk,2002	8 (93)	1.14 (0.91 to 1.49)
	**Infectious disease**		
	Balk,2002	6 (56)	0.93 (0.58 to 1.64)
	**Pediatrics**		
	Balk,2002	5 (60)	0.88 (0.49 to 1.51)
Allocation concealment	**Cardiovascular**		
	Balk,2002	8 (93)	1.14 (0.96 to 1.42)
	**Infectious disease**		
	Balk,2002	6 (56)	0.97 (0.68 to 1.42)
	Egger,2003	7 (55)	0.94 (0.76 to 1.16)
	**Pediatrics**		
	Balk,2002	5 (60)	0.90 (0.58 to 1.28)
	**Surgery**		
	Balk,2002	7 (67)	0.73 (0.36 to 1.24)
	**Neurology**		
	Egger,2003	5 (34)	0.44 (0.21 to 0.90)
	**Obstetrics & gynaecology**		
	Egger,2003	16 (122)	0.79 (0.67 to 0.94)
	**Miscellaneous**		
	Egger,2003	11 (93)	0.68 (0.52 to 0.89)
Blinding of participants	**Cardiovascular**		
	Balk,2002	8 (93)	1.08 (0.86 to 1.38)
	**Infectious disease**		
	Balk,2002	6 (56)	0.70 (0.46 to 1.11)
	**Pediatrics**		
	Balk,2002	5 (60)	0.79 (0.39 to 1.19)
Blinding of personnel	**Cardiovascular**		
	Balk,2002	8 (93)	1.09 (0.91 to 1.29)
	**Infectious disease**		
	Balk,2002	6 (56)	0.62 (0.43 to 0.91)
	**Pediatrics**		
	Balk,2002	5 (60)	1.13 (0.73 to 1.84)
Double blinding#	**Cardiovascular**		
	Balk,2002	8 (93)	1.10 (0.90 to 1.33)
	**Infectious disease**		
	Balk,2002	6 (56)	0.71 (0.47 to 1.12)
	Egger,2003	8 (62)	0.91 (0.39 to 2.17)
	**Pediatrics**		
	Balk,2002	5 (60)	1.05 (0.56 to 1.61)
	Neurology		
	Egger,2003	6 (57)	0.90 (0.61 to 1.33)
	**Neonatology**		
	Egger,2003	5 (34)	0.88 (0.63 to 1.25)
	**Obstetrics & gynaecology**		
	Egger,2003	11 (65)	0.96 (0.66 to 1.39)
	**Psychiatry**		
	Egger,2003	4 (52)	0.47 (0.26 to 0.84)
	**Miscellaneous**		
	Egger,2003	11 (129)	0.97 (0.79 to 1.20)
	**Complementary medicine**		
	Egger,2003	1 (6)	2.39 (0.63 to 9.02)
	**Non−complementary medicine**		
	Egger,2003	44 (393)	0.87 (0.75 to 1.03)
	**Benefit outcome**		
	Moustgaard,2020	74 (583)	1.02 (0.90 to 1.13)
	**Harm outcome**		
	Moustgaard,2020	20 (139)	0.64 (0.38 to 1.04)

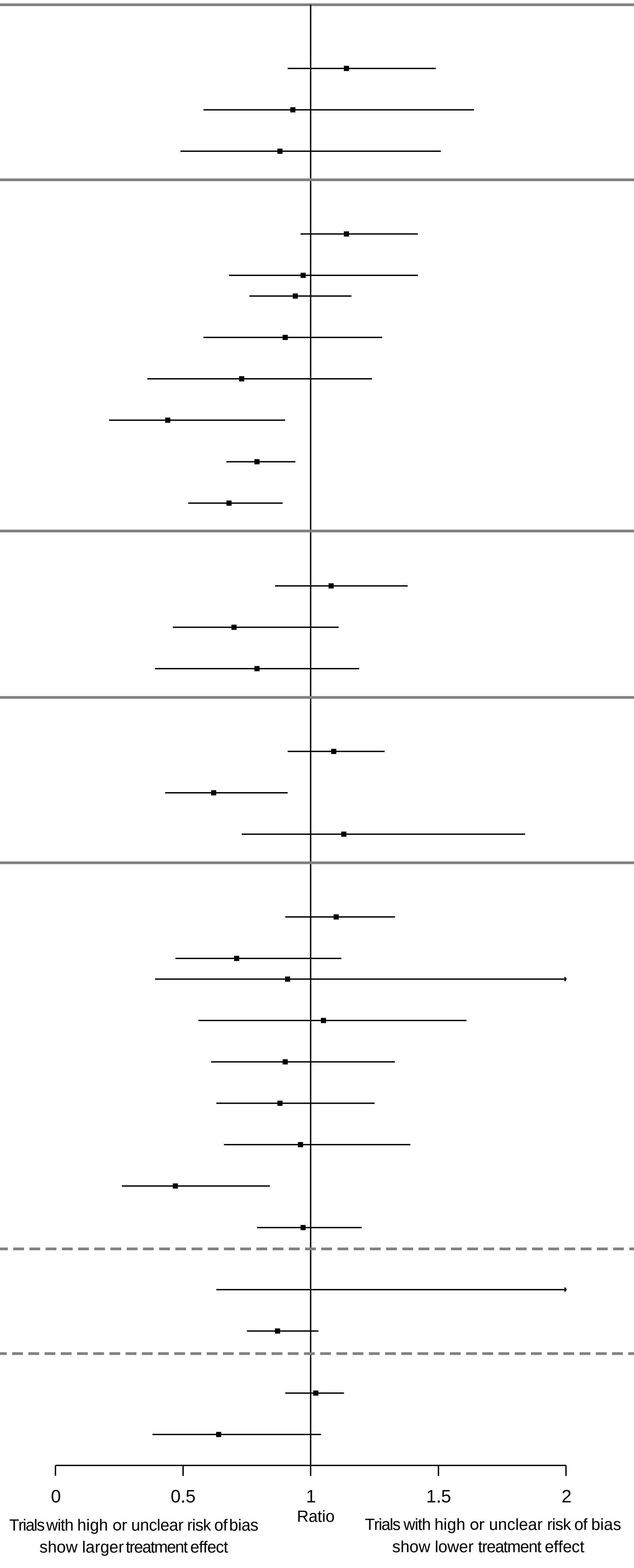

MA, meta-analyses; RCT, randomized controlled trials; CI, confidence interval
*Values are numbers of MA(RCTs) unless stated otherwise.
#Described as double-blinding or ≥2 key groups (participants, personnel, outcome assessors) were blinded.
