# Supplementary material for: Trial-level characteristics associate with treatment effect estimates: a systematic review of meta-epidemiological studies
Source: BMC Med Res Methodol. 2022 Jun 15;22:171. doi: 10.1186/s12874-022-01650-5 (PMC9202161; doi:10.1186/s12874-022-01650-5)
Supplement: Supplementary file 12 — Additional file 12: Appendix 12. Results of additional subgroup analyses. [file 12874_2022_1650_MOESM12_ESM.zip › Appendix 12-B-5.pdf]

Appendix 12-B-5

Trial−level characteristics	Subgroup	No. of MA (RCTs)*	Ratio of effect size (95%CI)
Sample size (larger sample vs smaller sample)	**Trial size (≥1000 vs ＜1000)**		
	**Adequate sequence generation** Kjaergard,2001	NR (81)	0.90 (0.47 to 1.76)
	**Inadequate sequence generation** Kjaergard,2001	NR (109)	0.46 (0.25 to 0.83)
	**Adequate allocation concealment** Kjaergard,2001	NR (68)	1.01 (0.48 to 2.11)
	**Inadequate allocation concealment** Kjaergard,2001	NR (122)	0.49 (0.27 to 0.86)
	**Adequate double blinding** Kjaergard,2001	NR (103)	0.84 (0.43 to 1.66)
	**Inadequate or no double blinding** Kjaergard,2001	NR (87)	0.52 (0.28 to 0.96)
	**Adequate follow−up** Kjaergard,2001	NR (133)	0.58 (0.32 to 1.02)
	**Inadequate follow−up** Kjaergard,2001	NR (57)	0.72 (0.30 to 1.71)
	**Trial size (≥100 vs ＜100 per arm)**		
	**Significant effect size of meta−analysis** Zhang,2013	8 (99)	0.49 (0.38 to 0.60)
	**Non−significant effect size of meta−analysis** Zhang,2013	19 (218)	0.69 (0.59 to 0.79)
	**Lowheterogeneity reported in meta−analysis** Zhang,2013	19 (200)	0.79 (0.68 to 0.90)
	**High heterogeneity reported in meta−analysis** Zhang,2013	8 (97)	0.46 (0.36 to 0.55)
Multicenter vs single center	**Cardiovascular disease** Balk,2002	8 (93)	1.30 (0.87 to 1.94)
	**Infectious disease** Balk,2002	6 (56)	0.96 (0.68 to 1.40)
	**Pediatrics** Balk,2002	5 (60)	1.74 (1.09 to 2.80)
	**Surgery** Balk,2002	7 (67)	0.71 (0.46 to 0.94)
Published trials vs grey literature	**Cardiology & angiology** Egger,2003	5 (144)	0.95 (0.76 to 1.19)
	**Gastroenterology** Egger,2003	4 (39)	1.19 (0.61 to 2.33)
	**Infectious diseases** Egger,2003	6 (78)	0.87 (0.72 to 1.05)
	**Neonatology** Egger,2003	5 (39)	0.99 (0.71 to 1.38)
	**Neurology** Egger,2003	6 (52)	0.94 (0.67 to 1.32)
	**Obstetrics & gynaecology** Egger,2003	12 (125)	1.34 (1.09 to 1.66)
	**Oncology** Egger,2003	4 (54)	0.96 (0.86 to 1.09)
	**Psychiatry** Egger,2003	5 (79)	1.61 (0.90 to 2.90)
	**Rheumatology & orthopaedics** Egger,2003	5 (63)	0.89 (0.52 to 1.51)
	**Miscellaneous** Egger,2003	8 (110)	1.24 (0.98 to 1.58)
	**Cochrane review** Egger,2003	36 (376)	1.14 (0.99 to 1.32)
	**Non−Cochrane review** Egger,2003	24 (407)	1.02 (0.91 to 1.15)
	**Complementary medicine** Egger,2003	1 (13)	3.09 (1.87 to 5.09)
	**Non−complementary medicine** Egger,2003	59 (770)	1.03 (0.96 to 1.11)
Parallel group vs cross−over	**Binary outcome** Lathyris,2007	10 (62)	0.94 (0.57 to 1.55)
	**Continuous outcome** Lathyris,2007	18 (207)	0.86 (0.67 to 1.10)
	**Only first period used in data analysis** Lathyris,2007	9 (NR)	0.87 (0.69 to 1.10)
	**Other or unclear period used in data analysis** Lathyris,2007	19 (NR)	0.87 (0.57 to 1.34)
	**Larger sample size in crossover trials** Lathyris,2007	12 (NR)	0.91 (0.73 to 1.13)
	**Larger sample size in parallel trials** Lathyris,2007	16 (NR)	0.91 (0.66 to 1.30)

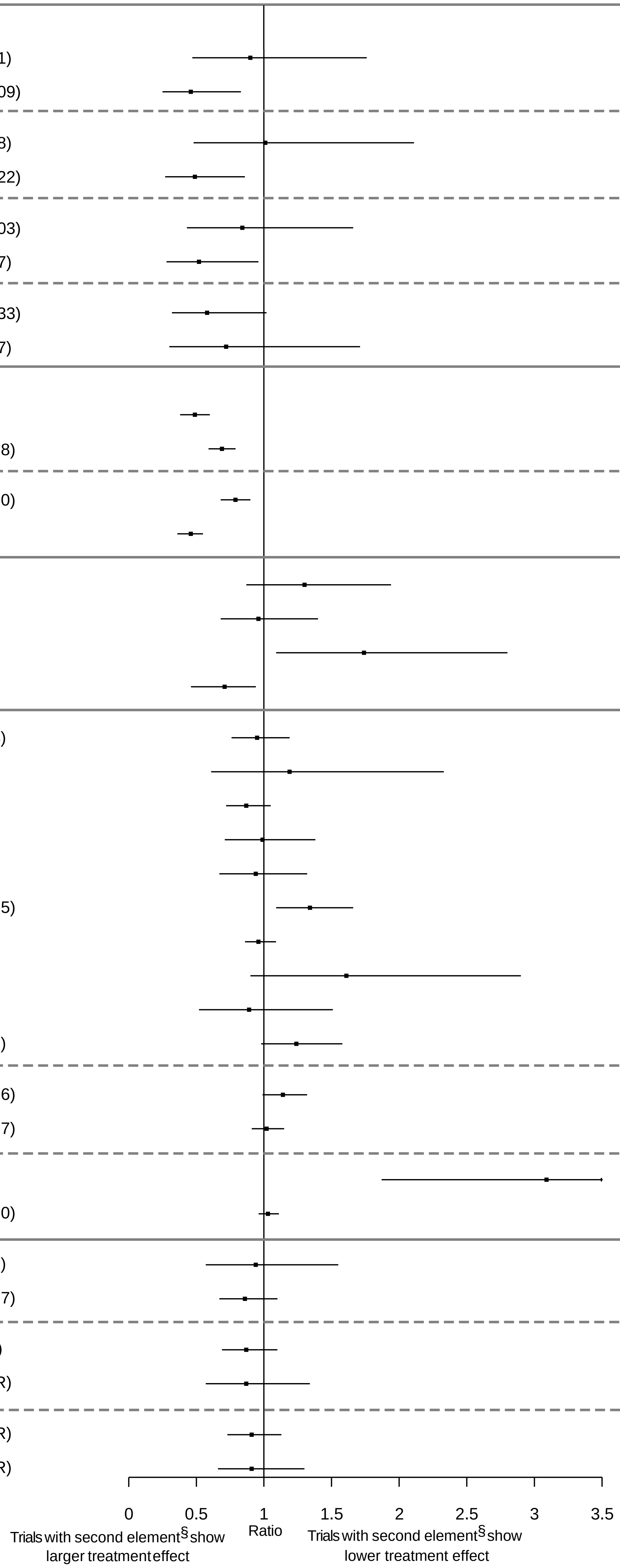

MA, meta-analyses; RCT, randomized controlled trial; NR, not reported; CI, confidence interval

*Values are numbers of MA (RCTs) unless stated otherwise.

§For example, sample size (larger sample vs smaller sample), smaller sample is regarded as second element.
