# Supplementary material for: Trial-level characteristics associate with treatment effect estimates: a systematic review of meta-epidemiological studies
Source: BMC Med Res Methodol. 2022 Jun 15;22:171. doi: 10.1186/s12874-022-01650-5 (PMC9202161; doi:10.1186/s12874-022-01650-5)
Supplement: Supplementary file 12 — Additional file 12: Appendix 12. Results of additional subgroup analyses. [file 12874_2022_1650_MOESM12_ESM.zip › Appendix 12-B-6.pdf]

Appendix 12-B-6

Trial−level characteristics	Subgroup	No. of MA (RCTs)*	Ratio of effect size (95%CI)
Outcomes measured in other ways vs PROMs	**Random−effects meta−analysis**		
	Berthelsen,2020	NR (205)	1.12 (0.92 to 1.39)
	**Fixed−effects meta−analysis**		
	Berthelsen,2020	NR (205)	1.08 (1.01 to 1.15)
	**Musculoskeletal system**		
	Berthelsen,2020	NR (40)	1.02 (0.62 to 1.69)
	**Respiratory system**		
	Berthelsen,2020	NR (36)	0.90 (0.53 to 1.54)
	**Genitourinary system/pregnancy**		
	Berthelsen,2020	NR (33)	0.73 (0.43 to 1.25)
	**Neoplasms**		
	Berthelsen,2020	NR (24)	1.45 (0.82 to 2.78)
	**Nervous system**		
	Berthelsen,2020	NR (22)	1.19 (0.60 to 2.33)
	**Circulatory system**		
	Berthelsen,2020	NR (20)	1.61 (0.80 to 3.23)
	**Conditions related to ext. causes/injury**		
	Berthelsen,2020	NR (7)	1.49 (0.41 to 5.56)
	**Other diseases**		
	Berthelsen,2020	NR (17)	0.79 (0.35 to 1.79)
	**Exercise intervention**		
	Berthelsen,2020	NR (111)	1.28 (0.94 to 1.75)
	**Manual therapy intervention**		
	Berthelsen,2020	NR (24)	1.69 (0.85 to 3.33)
	**Physical agents/mechanical modalities**		
	Berthelsen,2020	NR (22)	0.74 (0.36 to 1.54)
	**Electrotherapeutic modalities**		
	Berthelsen,2020	NR (13)	0.73 (0.29 to 1.81)
	**Education intervention**		
	Berthelsen,2020	NR (7)	0.69 (0.21 to 2.33)
	**Devices and equipment**		
	Berthelsen,2020	NR (5)	1.12 (0.20 to 4.76)
	**Functional training in self−care**		
	Berthelsen,2020	NR (3)	0.49 (0.08 to 3.23)
	**Airway clearance intervention**		
	Berthelsen,2020	NR (2)	0.12 (0.01 to 1.43)
	**Functional training in work**		
	Berthelsen,2020	NR (1)	2.27 (0.08 to 50.00)
	**Combination intervention**		
	Berthelsen,2020	NR (17)	1.15 (0.52 to 2.56)
	**Active intervention**		
	Berthelsen,2020	NR (71)	1.23 (0.93 to 1.64)
	**Passive intervention**		
	Berthelsen,2020	NR (134)	0.95 (0.64 to 1.41)
	**Usual care or no blank control**		
	Berthelsen,2020	NR (167)	1.16 (0.90 to 1.49)
	**Placebo or sham control**		
	Berthelsen,2020	NR (36)	0.99 (0.56 to 1.72)
	**Category of PROM**		
	**Quality of life**		
	Berthelsen,2020	NR (70)	0.89 (0.60 to 1.33)
	**Pain**		
	Berthelsen,2020	NR (56)	1.01 (0.65 to 1.56)
	**Symptom score**		
	Berthelsen,2020	NR (28)	2.00 (1.08 to 3.70)
	**Fatigue**		
	Berthelsen,2020	NR (20)	1.41 (0.68 to 2.94)
	**Physical function**		
	Berthelsen,2020	NR (13)	1.85 (0.74 to 4.76)
	**Depression and anxiety**		
	Berthelsen,2020	NR (10)	0.78 (0.28 to 2.17)
	**Dyspnea**		
	Berthelsen,2020	NR (7)	0.88 (0.23 to 3.45)
	**Other**		
	Berthelsen,2020	NR (1)	1.52 (0.06 to 33.3)
	**Category of non−PROM**		
	**Aerobic capacity/physical fitness**		
	Berthelsen,2020	NR (66)	1.82 (1.22 to 2.70)
	**Strength**		
	Berthelsen,2020	NR (21)	1.32 (0.65 to 2.70)
	**Mortality**		
	Berthelsen,2020	NR (18)	0.57 (0.27 to 1.19)
	**Clinician assessed scores/symptoms**		
	Berthelsen,2020	NR (17)	1.09 (0.49 to 2.44)
	**Range of motion**		
	Berthelsen,2020	NR (16)	1.00 (0.44 to 2.27)
	**Lung function**		
	Berthelsen,2020	NR (15)	0.47 (0.20 to 1.11)
	**Micturition/leakage/incontinence**		
	Berthelsen,2020	NR (12)	0.98 (0.40 to 2.38)
	**Cesarean section**		
	Berthelsen,2020	NR (7)	0.48 (0.15 to 1.56)
	**Biomarkers**		
	Berthelsen,2020	NR (6)	0.37 (0.09 to 1.54)
	**Use of analgesics**		
	Berthelsen,2020	NR (6)	1.27 (0.34 to 4.76)
	**Length of hospital stay**		
	Berthelsen,2020	NR (4)	0.45 (0.10 to 2.13)
	**Days of work/sick leave**		
	Berthelsen,2020	NR (3)	0.86 (0.15 to 5.00)
	**Other**		
	Berthelsen,2020	NR (14)	2.63 (1.14 to 6.25)
	**Clearly objective non−PROM**		
	Berthelsen,2020	NR (24)	0.52 (0.27 to 1.01)
	**Less objective non−PROM**		
	Berthelsen,2020	NR (181)	1.25 (0.98 to 1.61)
	**Clearly measure the same construct**		
	Berthelsen,2020	NR (24)	3.45 (1.82 to 6.67)
	**Not clearly measure the same construct**		
	Berthelsen,2020	NR (181)	0.97 (0.76 to 1.23)
	**Trial publication year＜2009**		
	Berthelsen,2020	NR (18)	0.72 (0.33 to 1.56)
	**Trial publication year≥2009**		
	Berthelsen,2020	NR (187)	1.18 (0.93 to 1.52)
	**Binary outcome**		
	Berthelsen,2020	NR (17)	0.95 (0.43 to 2.08)
	**Continuous outcome**		
	Berthelsen,2020	NR (138)	1.18 (0.88 to 1.56)
	**Combination outcome**		
	Berthelsen,2020	NR (50)	1.09 (0.68 to 1.72)

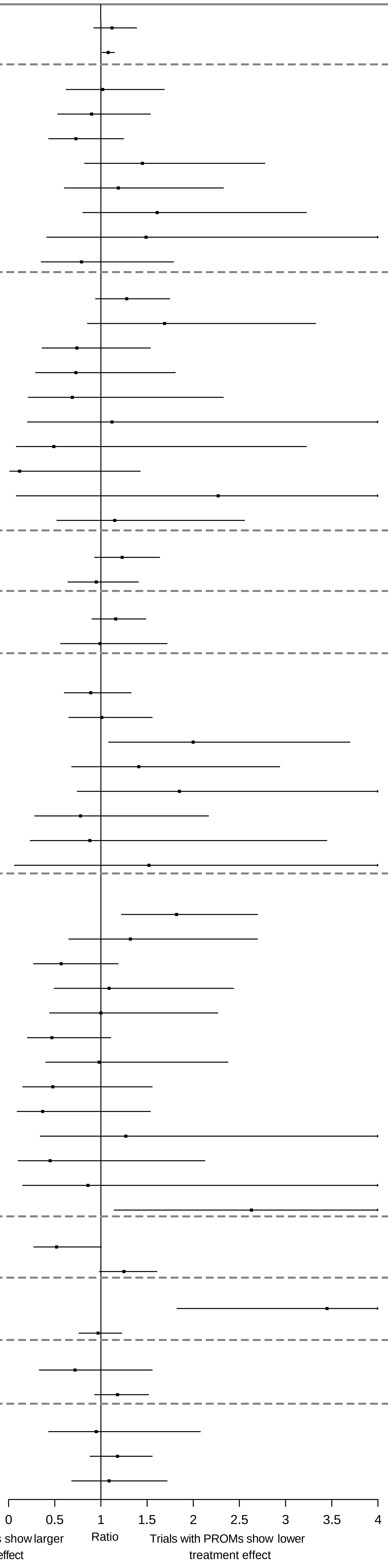

MA, meta-analyses; RCT, randomized controlled trial; CI, confidence interval; NR, not reported; PROMs, patient-reported outcome measures

*Values are numbers of MA(RCTs) unless stated otherwise.
