# Supplementary material for: Trial-level characteristics associate with treatment effect estimates: a systematic review of meta-epidemiological studies
Source: BMC Med Res Methodol. 2022 Jun 15;22:171. doi: 10.1186/s12874-022-01650-5 (PMC9202161; doi:10.1186/s12874-022-01650-5)
Supplement: Supplementary file 12 — Additional file 12: Appendix 12. Results of additional subgroup analyses. [file 12874_2022_1650_MOESM12_ESM.zip › Appendix 12-B-8.pdf]

Appendix 12-B-8

Trial−level characteristics	Subgroup (type of clinical areas)	No. of MA (RCTs)*	Ratio of effect size (95%CI)
**Study question well prespecified** (yes vs no)	**Cardiovascular disease**		
	Balk,2002	8 (93)	0.98 (0.68 to 1.30)
	**Infectious disease**		
	Balk,2002	6 (56)	0.58 (0.32 to 1.80)
	**Surgery**		
	Balk,2002	7 (67)	0.81 (0.42 to 1.40)
**Placebo control** (yes vs no)	**Cardiovascular disease**		
	Balk,2002	8 (93)	1.03 (0.84 to 1.27)
	**Infectious disease**		
	Balk,2002	6 (56)	0.62 (0.40 to 0.91)
	**Pediatrics**		
	Balk,2002	5 (60)	0.40 (0.22 to 0.86)
**US vs country other than US**	**Cardiovascular disease**		
	Balk,2002	8 (93)	1.12 (0.94 to 1.38)
	**Infectious disease**		
	Balk,2002	6 (56)	0.87 (0.55 to 1.29)
	**Pediatrics**		
	Balk,2002	5 (60)	1.02 (0.55 to 1.58)
	**Surgery**		
	Balk,2002	7 (67)	0.84 (0.49 to 1.38)
**Selection criteria reported** (yes vs no)	**Infectious disease**		
	Balk,2002	6 (56)	1.73 (0.81 to 5.49)
	**Surgery**		
	Balk,2002	7 (67)	0.65 (0.45 to 1.11)
**Whether described method of randomization** (yes vs no)	**Cardiovascular disease**		
	Balk,2002	8 (93)	1.14 (0.95 to 1.40)
	**Infectious disease**		
	Balk,2002	6 (56)	1.13 (0.73 to 1.67)
	**Pediatrics**		
	Balk,2002	5 (60)	1.00 (0.63 to 1.46)
	**Surgery**		
	Balk,2002	7 (67)	0.76 (0.53 to 1.08)
**Whether the statistical methods is valid** (yes vs no)	**Cardiovascular disease**		
	Balk,2002	8 (93)	1.03 (0.81 to 1.33)
	**Infectious disease**		
	Balk,2002	6 (56)	1.17 (0.78 to 1.77)
	**Pediatrics**		
	Balk,2002	5 (60)	0.97 (0.48 to 1.73)
	**Surgery**		
	Balk,2002	7 (67)	1.63 (1.03 to 2.83)
**Statistician involvement** (yes vs no)	**Cardiovascular disease**		
	Balk,2002	8 (93)	1.05 (0.86 to 1.29)
	**Infectious disease**		
	Balk,2002	6 (56)	0.85 (0.59 to 1.28)
	**Pediatrics**		
	Balk,2002	5 (60)	1.12 (0.81 to 1.60)
	**Surgery**		
	Balk,2002	7 (67)	1.13 (0.73 to 1.67)
**Intention to treat analyses** (yes vs no)	**Cardiovascular disease**		
	Balk,2002	6 (56)	0.89 (0.60 to 1.25)
	**Infectious disease**		
	Balk,2002	5 (60)	0.80 (0.42 to 1.37)
	**Surgery**		
	Balk,2002	7 (67)	1.14 (0.73 to 2.05)
**Power calculation reported** (yes vs no)	**Cardiovascular disease**		
	Balk,2002	8 (93)	1.04 (0.89 to 1.22)
	**Infectious disease**		
	Balk,2002	6 (56)	1.32 (0.88 to 1.95)
	**Pediatrics**		
	Balk,2002	5 (60)	1.13 (0.76 to 1.62)
	**Surgery**		
	Balk,2002	7 (67)	0.91 (0.55 to 1.43)
**Whether baseline characteristics reported** (yes vs no)	**Cardiovascular disease**		
	Balk,2002	8 (93)	1.19 (0.69 to 1.96)
	**Infectious disease**		
	Balk,2002	6 (56)	1.18 (0.66 to 2.21)
	**Pediatrics**		
	Balk,2002	5 (60)	0.66 (0.35 to 1.29)
**Baseline imbalance** (no vs yes)	**Cardiovascular disease**		
	Balk,2002	8 (93)	1.03 (0.73 to 1.33)
	**Infectious disease**		
	Balk,2002	6 (56)	1.15 (0.82 to 1.71)
	**Pediatrics**		
	Balk,2002	5 (60)	1.13 (0.73 to 1.66)
	**Surgery**		
	Balk,2002	7 (67)	0.77 (0.51 to 1.30)
**Confounders accounted for** (yes vs no)	**Cardiovascular disease**		
	Balk,2002	8 (93)	0.94 (0.60 to 1.49)
	**Pediatrics**		
	Balk,2002	5 (60)	0.96 (0.50 to 1.65)
	**Surgery**		
	Balk,2002	7 (67)	1.20 (0.76 to 1.72)
**Dropouts reported** (yes vs no)	**Infectious disease**		
	Balk,2002	6 (56)	1.11 (0.60 to 1.91)
	**Surgery**		
	Balk,2002	7 (67)	1.12 (0.54 to 2.33)
**Reason for dropouts reported** (yes vs no)	**Infectious disease**		
	Balk,2002	6 (56)	0.94 (0.75 to 1.17)
	**Pediatrics**		
	Balk,2002	5 (60)	0.94 (0.55 to 1.52)
	**Surgery**		
	Balk,2002	7 (67)	0.70 (0.43 to 1.16)
**Percentage of dropouts** (low vs high)	**Cardiovascular disease**		
	Balk,2002	8 (93)	1.00 (0.89 to 1.14)
	**Infectious disease**		
	Balk,2002	6 (56)	1.07 (0.84 to 1.34)
	**Pediatrics**		
	Balk,2002	5 (60)	1.17 (0.92 to 1.57)
	**Surgery**		
	Balk,2002	7 (67)	1.03 (0.86 to 1.27)
**Whether the conclusions is valid** (yes vs no)	**Infectious disease**		
	Balk,2002	6 (56)	0.79 (0.57 to 1.11)
	**Surgery**		
	Balk,2002	7 (67)	0.71 (0.30 to 1.46)

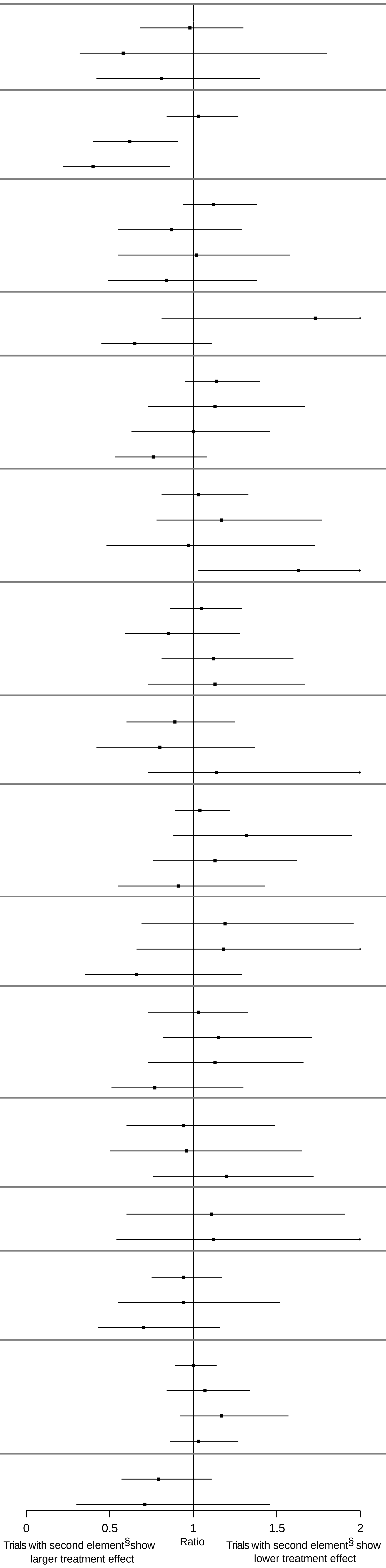

MA, meta-analyses; RCT, randomized controlled trial; CI, confidence interval

*Values are numbers of MA (RCTs) unless stated otherwise.

§For example, dropouts reported (yes vs no), not reported dropouts is regarded as second element.
