# Supplementary material for: Trial-level characteristics associate with treatment effect estimates: a systematic review of meta-epidemiological studies
Source: BMC Med Res Methodol. 2022 Jun 15;22:171. doi: 10.1186/s12874-022-01650-5 (PMC9202161; doi:10.1186/s12874-022-01650-5)
Supplement: Supplementary file 12 — Additional file 12: Appendix 12. Results of additional subgroup analyses. [file 12874_2022_1650_MOESM12_ESM.zip › Appendix 12-C-1.pdf]

Appendix 12-C-1

Trial−level characteristics	Subgroup	No. of MA (RCTs)*	Difference of effect size (95%CI)
**Statistical power of studies** (adequately powered vs power <50%)	**Cancer**		
	Turner,2013	NR	−0.01 (−0.08 to 0.07)
	**Cardiovascular**		
	Turner,2013	NR	−0.12 (−0.18 to −0.06)
	**Central nervous system/musculoskeletal**		
	Turner,2013	NR	0.04 (−0.08 to 0.16)
	**Digestive**		
	Turner,2013	NR	−0.01 (−0.10 to 0.08)
	**Gynaecology, pregnancy and birth**		
	Turner,2013	NR	−0.14 (−0.19 to −0.09)
	**Infectious diseases**		
	Turner,2013	NR	−0.20 (−0.30 to −0.09)
	**Mental health and behavioural conditions**		
	Turner,2013	NR	−0.15 (−0.22 to −0.08)
	**Pathological conditions, symptoms and signs**		
	Turner,2013	NR	−0.03 (−0.18 to 0.11)
	**Respiratory diseases**		
	Turner,2013	NR	−0.08 (−0.17 to −0.00)
	**Urogenital**		
	Turner,2013	NR	−0.06 (−0.19 to 0.06)
	**Other medical specialties**		
	Turner,2013	NR	−0.21 (−0.30 to −0.12)
	**Pharmacological vs Control/Placebo comparison**		
	Turner,2013	NR	−0.15 (−0.18 to −0.11)
	**Non−pharmacological vs Control/Placebo comparison**		
	Turner,2013	NR	−0.12 (−0.17 to −0.07)
	**Activevs Activecomparison**		
	Turner,2013	NR	−0.03 (−0.07 to 0.01)
**First trial vs subsequent trial**	**First trials < 300 participants**		
	Gartlehner,2016	NR	0.25 (0.18 to 0.33)
	**First trials > 300 participants**		
	Gartlehner,2016	NR	0.14 (0.08 to 0.20)
	**First trials with low risk ofbias ratings**		
	Gartlehner,2016	NR	0.13 (0.07 to 0.20)
	**First trials with unclear risk of bias ratings**		
	Gartlehner,2016	NR	0.30 (0.20 to 0.39)
	**First trialswith high risk ofbias ratings**		
	Gartlehner,2016	NR	0.14 (0.07 to 0.21)
	**First trials with treatment effects ≤ 0.5 SMDs**		
	Gartlehner,2016	NR	0.04 (0.02 to 0.05)
	**First trials with treatment effects > 0.5 SMDs**		
	Gartlehner,2016	NR	0.68 (0.50 to 0.86)
**Trial registration** (yes vs no)	**Activevs.passiveself−ligatingbracketsforcomprehensivetreatment**		
	Papageorgiou,2018	1 (NR)	−0.38 (−1.81 to 1.04)
	**Maxillaryprotraction vs. untreatedcontrol formaxillarydeficiency**		
	Papageorgiou,2018	3 (NR)	−0.96 (−1.66 to −0.27)
	**Yes vs. no chewing gum for comprehensive treatment**		
	Papageorgiou,2018	1 (NR)	−2.06 (−5.55 to 1.42)
	**Light vs. no light therapy for comprehensive treatment**		
	Papageorgiou,2018	1 (NR)	−0.37 (−5.71 to 4.98)
	**Temporaryvs.conventionalanchoragereinforcementduringextraction**		
	Papageorgiou,2018	3 (NR)	−0.56 (−1.40 to 0.29)
	**Self−ligatingvs. conventional bracketsfor comprehensive treatment**		
	Papageorgiou,2018	6 (NR)	−0.22 (−0.52 to 0.08)
	**Vibrationvs. no vibration adjuncts for comprehensive treatment**		
	Papageorgiou,2018	1 (NR)	−0.23 (−1.27 to 0.82)
	**Treatment efficacy**		
	Papageorgiou,2018	12 (NR)	−0.51 (−0.83 to −0.19)
	**Adverse effects**		
	Papageorgiou,2018	4 (NR)	−0.17 (−0.53 to 0.19)
	**Negative is favorable intervention effect**		
	Papageorgiou,2018	12 (NR)	−0.29 (−0.59 to −0.00)
	**Positiveisfavorable intervention effect**		
	Papageorgiou,2018	4 (NR)	−0.49 (−0.90 to −0.07)

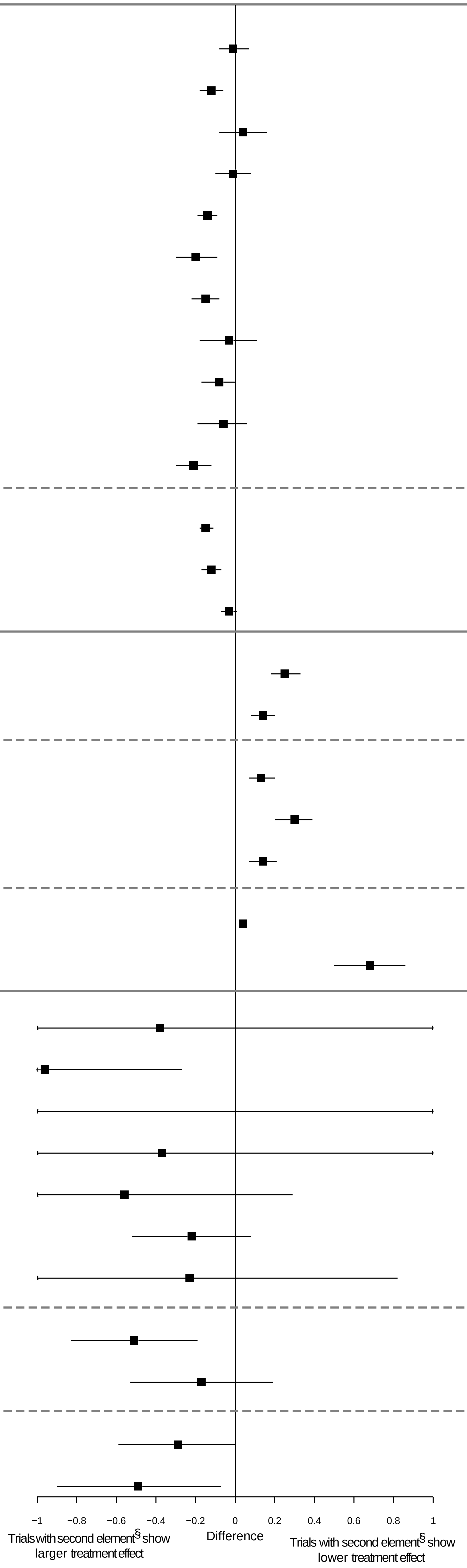

MA, meta-analyses; RCT, randomized controlled trial; NR, not reported; CI, confidence interval

*Values are numbers of MA (RCTs) unless stated otherwise.

§For example, first trial vs subsequent trial, subsequent trial is regarded as second element.
