# Supplementary material for: Trial-level characteristics associate with treatment effect estimates: a systematic review of meta-epidemiological studies
Source: BMC Med Res Methodol. 2022 Jun 15;22:171. doi: 10.1186/s12874-022-01650-5 (PMC9202161; doi:10.1186/s12874-022-01650-5)
Supplement: Supplementary file 12 — Additional file 12: Appendix 12. Results of additional subgroup analyses. [file 12874_2022_1650_MOESM12_ESM.zip › Appendix 12-C-2.pdf]

Appendix 12-C-2

Trial−level characteristics	Subgroup	No. of MA (RCTs)*	Difference of effect size (95%CI)
Blinding of outcome assessors	**Facial wrinkles**		
	Hrobjartsson,2013	NR (3)	−0.39 (−1.03 to 0.25)
	**Angina pectoris**		
	Hrobjartsson,2013	NR (2)	−0.56 (−0.90 to −0.23)
	**Parkinson disease**		
	Hrobjartsson,2013	NR (2)	−0.17 (−0.49 to 0.15)
	**Other diseases other than aforementioned**		
	Hrobjartsson,2013	NR (9)	−0.06 (−0.21 to 0.10)
	**Periodontics and implantology**		
	Saltaji,2018(b)	NR	0.01 (−0.17 to 0.20)
	**Dental public health**		
	Saltaji,2018(b)	NR	0.41 (−0.23 to 1.06)
	**Other dental specialty**		
	Saltaji,2018(b)	NR	−0.11 (−0.26 to 0.05)
	**Observer bias main objective**		
	Hrobjartsson,2013	NR (4)	−0.07 (−0.41 to 0.28)
	**Observer bias not main objective**		
	Hrobjartsson,2013	NR (12)	−0.27 (−0.47 to -0.06)
	**Parallel group**		
	Hrobjartsson,2013	NR (13)	−0.27 (−0.49 to −0.06)
	**Crossover/split−body**		
	Hrobjartsson,2013	NR (3)	−0.08 (−0.35 to 0.20)
	**Same type of outcome assessors (neurologists vs neurologists)**		
	Hrobjartsson,2013	NR (10)	−0.13 (−0.27 to 0.01)
	**Not same type of outcome assessors (neurologists vs physiotherapists)**		
	Hrobjartsson,2013	NR (6)	−0.30 (−0.69 to 0.09)
	**Same type of blinding procedure (clinical exam vs clinical exam)**		
	Hrobjartsson,2013	NR (6)	−0.09 (−0.24 to 0.06)
	**Not same type of blinding procedure (clinical exam vs video of clinical exam)**		
	Hrobjartsson,2013	NR (10)	−0.30 (−0.57 to −0.02)
	**All seen by both blinded and non-blinded assessors**		
	Hrobjartsson,2013	NR (6)	−0.29 (−0.49 to −0.09)
	**A minority seen only by one type of assessor**		
	Hrobjartsson,2013	NR (10)	−0.20 (−0.46 to 0.06)
	**Small treatment benefit (effect size＜−0.5) in overall meta−analysis**		
	Saltaji,2018(b)	NR	−0.09 (−0.22 to 0.03)
	**Large treatment benefit (effect size≥−0.5) in overall meta−analysis**		
	Saltaji,2018(b)	NR	0.15 (−0.16 to 0.46)
	**Low heterogeneity (τ^2<0.06) between trials in overall meta−analysis**		
	Saltaji,2018(b)	NR	−0.07 (−0.21 to 0.07)
	**High heterogeneity (τ^2≥0.06) between trials in overall meta−analysis**		
	Saltaji,2018(b)	NR	−0.04 (−0.26 to 0.18)
	**Individual patient data**		
	Hrobjartsson,2013	NR (1)	−0.16 (−0.65 to 0.34)
	**Correlation data with no individual patient data**		
	Hrobjartsson,2013	NR (6)	−0.46 (−0.83 to −0.10)
	**Basic outcomedata with no information on correlation**		
	Hrobjartsson,2013	NR (9)	−0.06 (−0.20 to 0.07)
	**SMD standardized by SD of blinded control group**		
	Hrobjartsson,2013	NR (16)	−0.26 (−0.44 to −0.08)
	**SMD standardized separately for blinded and non-blinded assessors**		
	Hrobjartsson,2013	NR (16)	−0.23 (−0.40 to −0.06)
	**Correlation accounted for by correlation coefficient**		
	Hrobjartsson,2013	NR (7)	−0.36 (−0.64 to −0.08)
	**Correlation accounted for by correlation coefficient or median correlation coefficient**		
	Hrobjartsson,2013	NR (16)	−0.21 (−0.35 to −0.07)
	**Increased precision in crossover/split−body trials was accounted for**		
	Hrobjartsson,2013	NR (16)	−0.22 (−0.39 to −0.06)

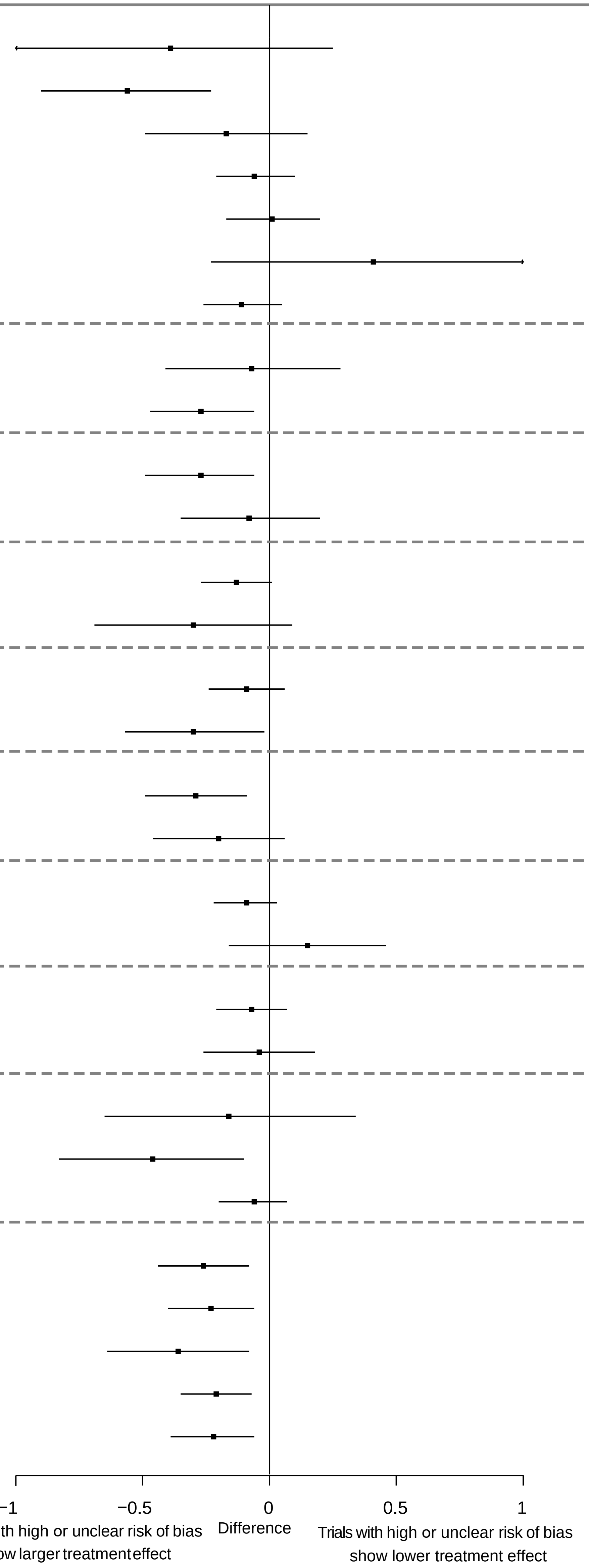

MA, meta-analyses; RCT, randomized controlled trial; CI, confidence interval; NR, not reported; SMD, standardized mean difference; SD, standard deviation
*Values are numbers of MA(RCTs) unless stated otherwise.
