# Supplementary material for: Trial-level characteristics associate with treatment effect estimates: a systematic review of meta-epidemiological studies
Source: BMC Med Res Methodol. 2022 Jun 15;22:171. doi: 10.1186/s12874-022-01650-5 (PMC9202161; doi:10.1186/s12874-022-01650-5)
Supplement: Supplementary file 12 — Additional file 12: Appendix 12. Results of additional subgroup analyses. [file 12874_2022_1650_MOESM12_ESM.zip › Appendix 12-C-5.pdf]

Appendix 12-C-5

Trial−level characteristics	Subgroup	No. of MA (RCTs)*	Difference of effect size (95%CI)
Blinding of personnel	**Small treatment benefit (effect size＜−0.5) in overall meta−analysis**		
	Saltaji,2018(b)	NR	−0.03 (−0.10 to 0.04)
	**Large treatment benefit (effect size≥−0.5) in overall meta−analysis**		
	Saltaji,2018(b)	NR	0.34 (−0.12 to 0.81)
	**Low heterogeneity (τ^2<0.06) between trials in overall meta−analysis**		
	Saltaji,2018(b)	NR	−0.03 (−0.09 to 0.04)
	**High heterogeneity (τ^2≥0.06) between trials in overall meta−analysis**		
	Saltaji,2018(b)	NR	0.45 (−0.16 to 1.06)
	**Periodontics and implantology**		
	Saltaji,2018(b)	NR	0.32 (−0.10 to 0.74)
	**Dental public health**		
	Saltaji,2018(b)	NR	−0.43 (−1.31 to 0.46)
	**Other dental specialty**		
	Saltaji,2018(b)	NR	−0.03 (−0.10 to 0.04)
Blinding of principal−investigator	**Small treatment benefit (effect size＜−0.5) in overall meta−analysis**		
	Saltaji,2018(b)	NR	0.02 (−0.08 to 0.12)
	**Large treatment benefit (effect size≥−0.5) in overall meta−analysis**		
	Saltaji,2018(b)	NR	−0.08 (−0.53 to 0.37)
	**Low heterogeneity (τ^2<0.06) between trials in overall meta−analysis**		
	Saltaji,2018(b)	NR	0.03 (−0.07 to 0.12)
	**High heterogeneity (τ^2≥0.06) between trials in overall meta−analysis**		
	Saltaji,2018(b)	NR	−0.13 (−0.55 to 0.28)
	**Periodontics and implantology**		
	Saltaji,2018(b)	NR	0.05 (−0.29 to 0.40)
	**Dental public health**		
	Saltaji,2018(b)	NR	−0.09 (−0.43 to 0.24)
	**Other dental specialty**		
	Saltaji,2018(b)	NR	0.01 (−0.09 to 0.12)
Double blinding#	**Small treatment benefit (effect size＜−0.5) in overall meta−analysis**		
	Saltaji,2018(b)	NR	−0.12 (−0.26 to 0.02)
	**Large treatment benefit (effect size≥−0.5) in overall meta−analysis**		
	Saltaji,2018(b)	NR	0.23 (−0.21 to 0.68)
	**Low heterogeneity (τ^2<0.06) between trials in overall meta−analysis**		
	Saltaji,2018(b)	NR	−0.04 (−0.19 to 0.10)
	**High heterogeneity (τ^2≥0.06) between trials in overall meta−analysis**		
	Saltaji,2018(b)	NR	−0.25 (−0.54 to 0.04)
	**Periodontics and implantology**		
	Saltaji,2018(b)	NR	−0.02 (−0.37 to 0.33)
	**Dental public health**		
	Saltaji,2018(b)	NR	−0.11 (−0.73 to 0.51)
	**Other dental specialty**		
	Saltaji,2018(b)	NR	0.10 (−0.25 to 0.05)
Blinding of participants and outcome assessors	**Small treatment benefit (effect size＜−0.5) in overall meta−analysis**		
	Saltaji,2018(b)	NR	−0.20 (−0.33 to −0.06)
	**Large treatment benefit (effect size≥−0.5) in overall meta−analysis**		
	Saltaji,2018(b)	NR	0.05 (−0.60 to 0.70)
	**Low heterogeneity (τ^2<0.06) between trials in overall meta−analysis**		
	Saltaji,2018(b)	NR	−0.17 (−0.30 to −0.03)
	**High heterogeneity (τ^2≥ 0.06) between trials in overall meta −analysis**		
	Saltaji,2018(b)	NR	−0.35 (−0.73 to 0.04)
	**Periodontics and implantology**		
	Saltaji,2018(b)	NR	−0.35 (−1.02 to 0.32)
	**Dental public health**		
	Saltaji,2018(b)	NR	0.10 (−0.77 to 0.97)
	**Other dental specialty**		
	Saltaji,2018(b)	NR	−0.18 (−0.32 to 0.05)
Blinding of participants,personnel and outcome assessors	**Small treatment benefit (effect size＜−0.5) in overall meta−analysis**		
	Saltaji,2018(b)	NR	−0.14 (−0.26 to −0.02)
	**Large treatment benefit (effect size≥−0.5) in overall meta−analysis**		
	Saltaji,2018(b)	NR	−0.27 (−1.20 to 0.65)
	**Low heterogeneity (τ^2<0.06) between trials in overall meta−analysis**		
	Saltaji,2018(b)	NR	−0.14 (−0.26 to −0.02)
	**High heterogeneity (τ^2≥0.06) between trials in overall meta−analysis**		
	Saltaji,2018(b)	NR	−0.17 (−1.34 to 0.99)
	**Periodontics and implantology**		
	Saltaji,2018(b)	NR	−0.25 (−1.20 to 0.70)
	**Dental public health**		
	Saltaji,2018(b)	NR	−0.05 (−1.24 to 1.14)
	**Other dental specialty**		
	Saltaji,2018(b)	NR	−0.14 (−0.26 to −0.02)
Using an appropriate method of blinding	**Small treatment benefit (effect size＜−0.5) in overall meta−analysis**		
	Saltaji,2018(b)	NR	−0.12 (−0.24 to 0.01)
	**Large treatment benefit (effect size ≥−0.5) in overall meta−analysis**		
	Saltaji,2018(b)	NR	0.25 (−0.04 to 0.54)
	**Low heterogeneity (τ^2<0.06) between trials in overall meta−analysis**		
	Saltaji,2018(b)	NR	−0.05 (−0.19 to 0.09)
	**High heterogeneity (τ^2≥0.06) between trials in overall meta−analysis**		
	Saltaji,2018(b)	NR	−0.08 (−0.31 to 0.14)
	**Periodontics and implantology**		
	Saltaji,2018(b)	NR	0.04 (−0.16 to 0.25)
	**Dental public health**		
	Saltaji,2018(b)	NR	0.44 (−0.09 to 0.96)
	**Other dental specialty**		
	Saltaji,2018(b)	NR	−0.11 (−0.25 to 0.04)

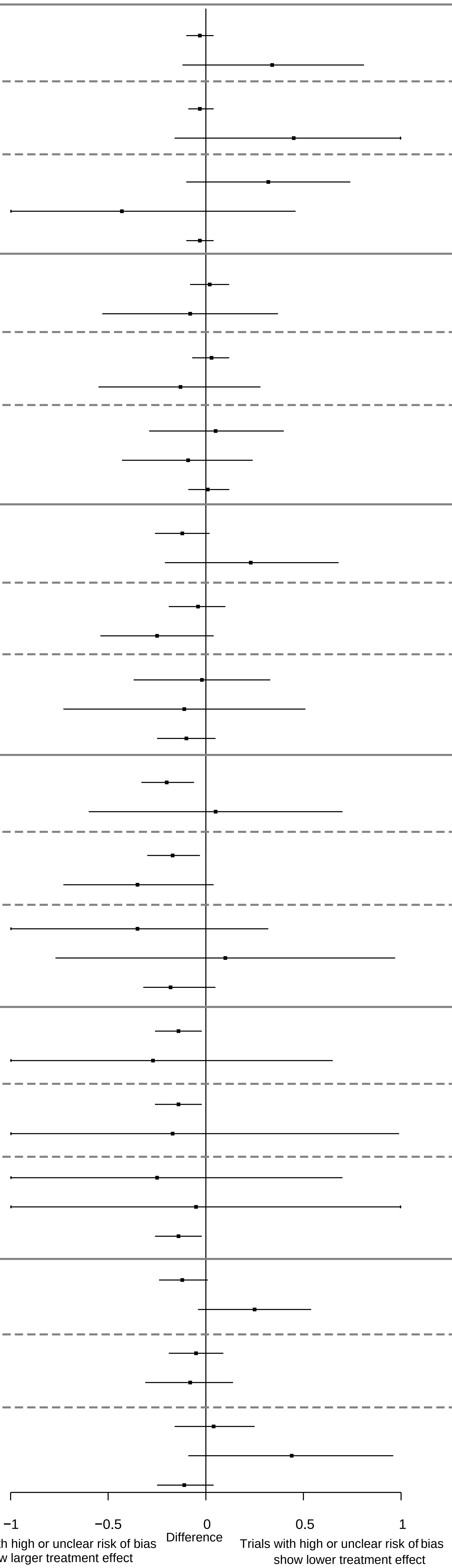

MA, meta-analyses; RCT, randomized controlled trial; CI, confidence interval; NR, not reported
*Values are numbers of MA(RCTs) unless stated otherwise.
#Described as double-blinding
