# Supplementary figures and images for: Trial-level characteristics associate with treatment effect estimates: a systematic review of meta-epidemiological studies
Source: BMC Med Res Methodol. 2022 Jun 15;22:171. doi: 10.1186/s12874-022-01650-5 (PMC9202161; doi:10.1186/s12874-022-01650-5)

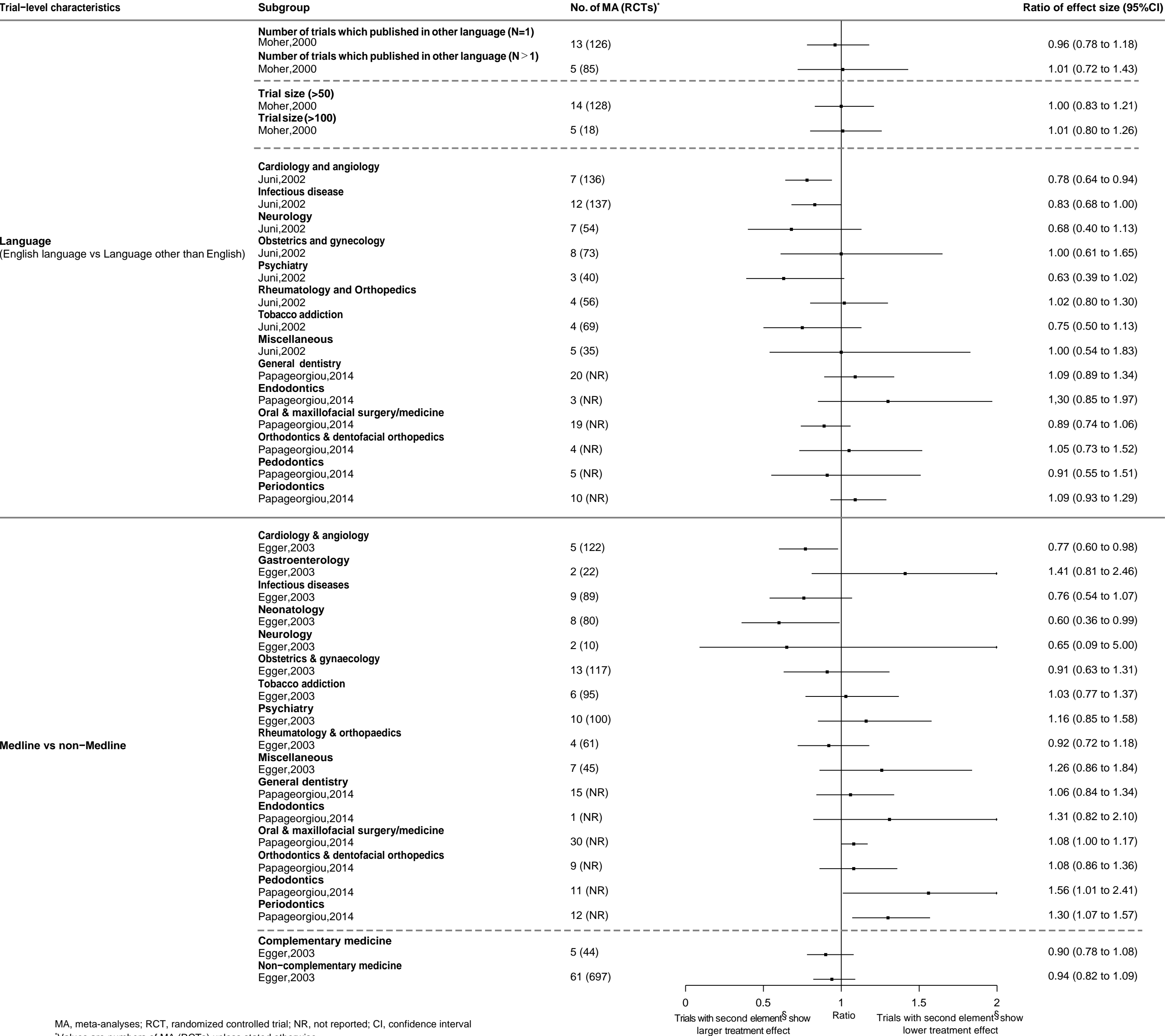

Supplement: Supplementary file 12 — Additional file 12: Appendix 12. Results of additional subgroup analyses. [file 12874_2022_1650_MOESM12_ESM.zip › Appendix 12-B-4.pdf]

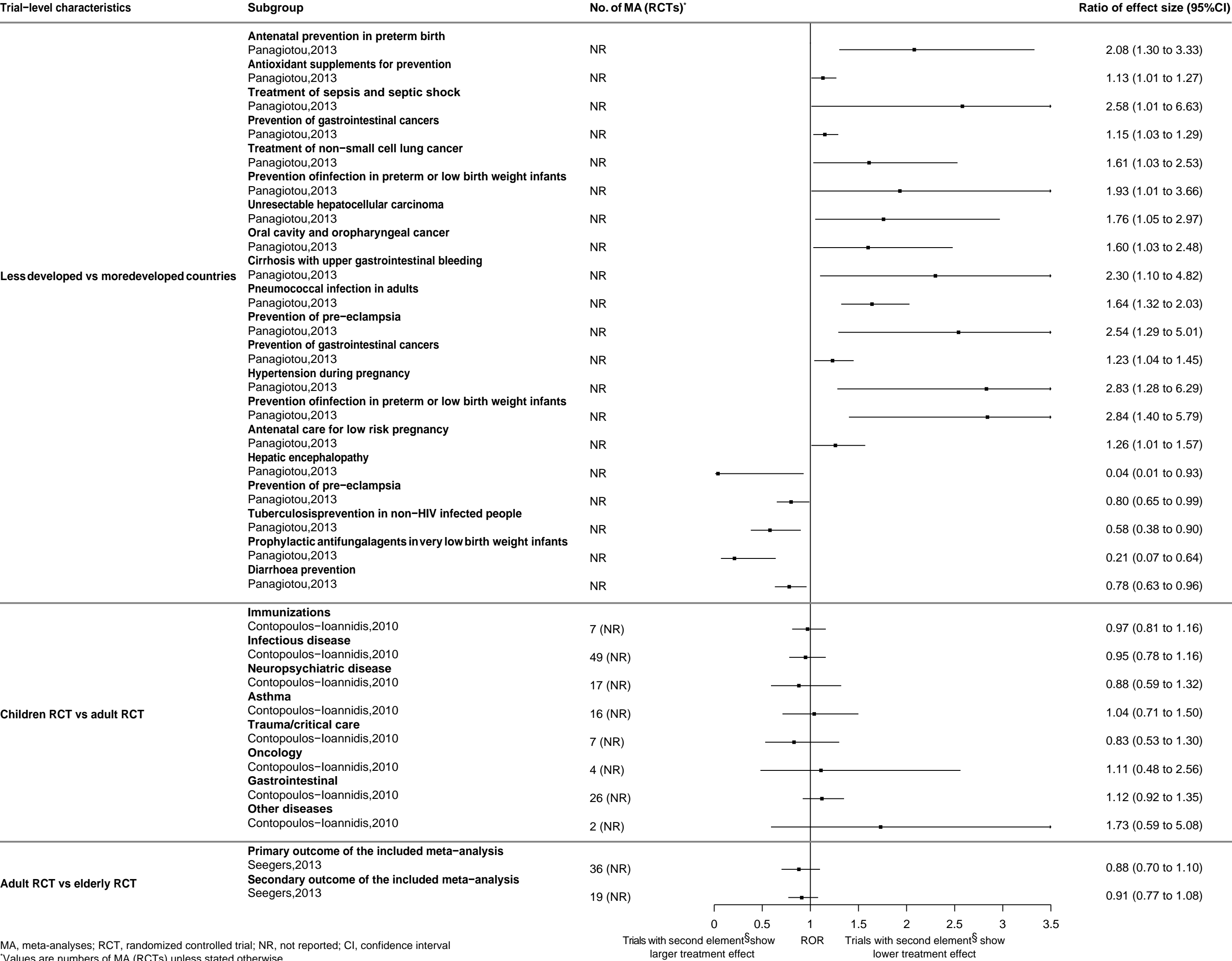

Supplement: Supplementary file 12 — Additional file 12: Appendix 12. Results of additional subgroup analyses. [file 12874_2022_1650_MOESM12_ESM.zip › Appendix 12-B-7.pdf]

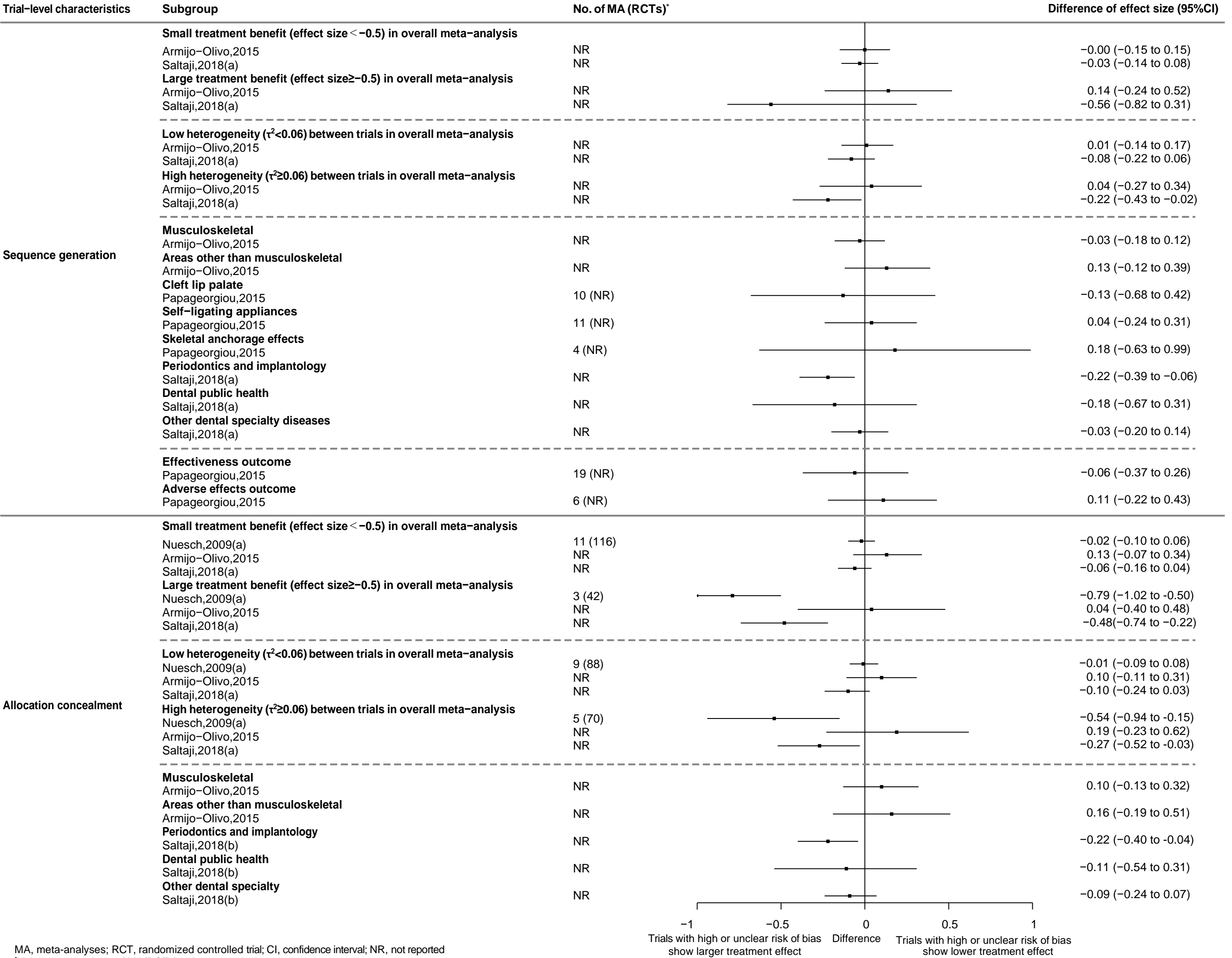

Supplement: Supplementary file 12 — Additional file 12: Appendix 12. Results of additional subgroup analyses. [file 12874_2022_1650_MOESM12_ESM.zip › Appendix 12-C-3.pdf]

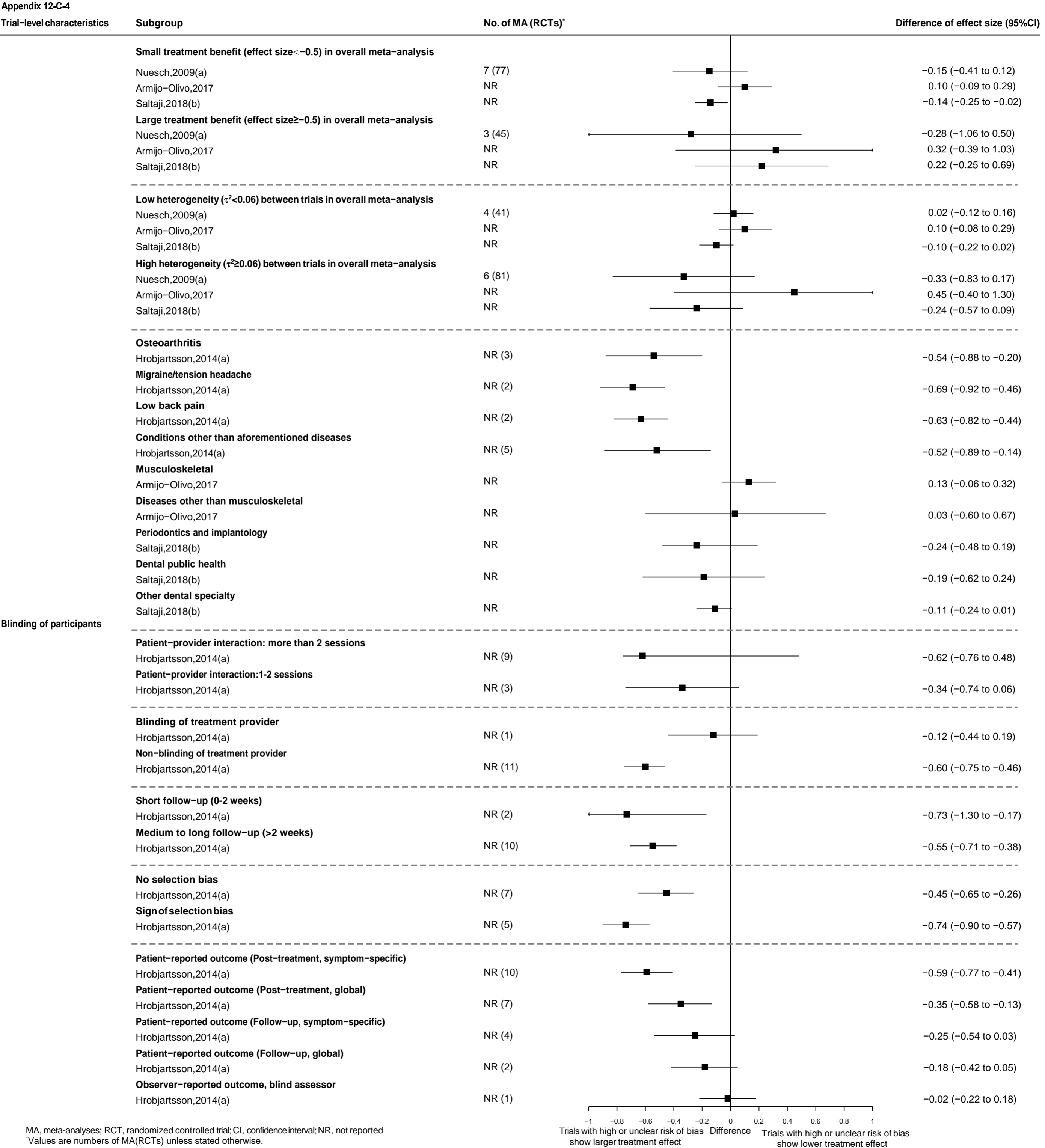

Supplement: Supplementary file 12 — Additional file 12: Appendix 12. Results of additional subgroup analyses. [file 12874_2022_1650_MOESM12_ESM.zip › Appendix 12-C-4.pdf]

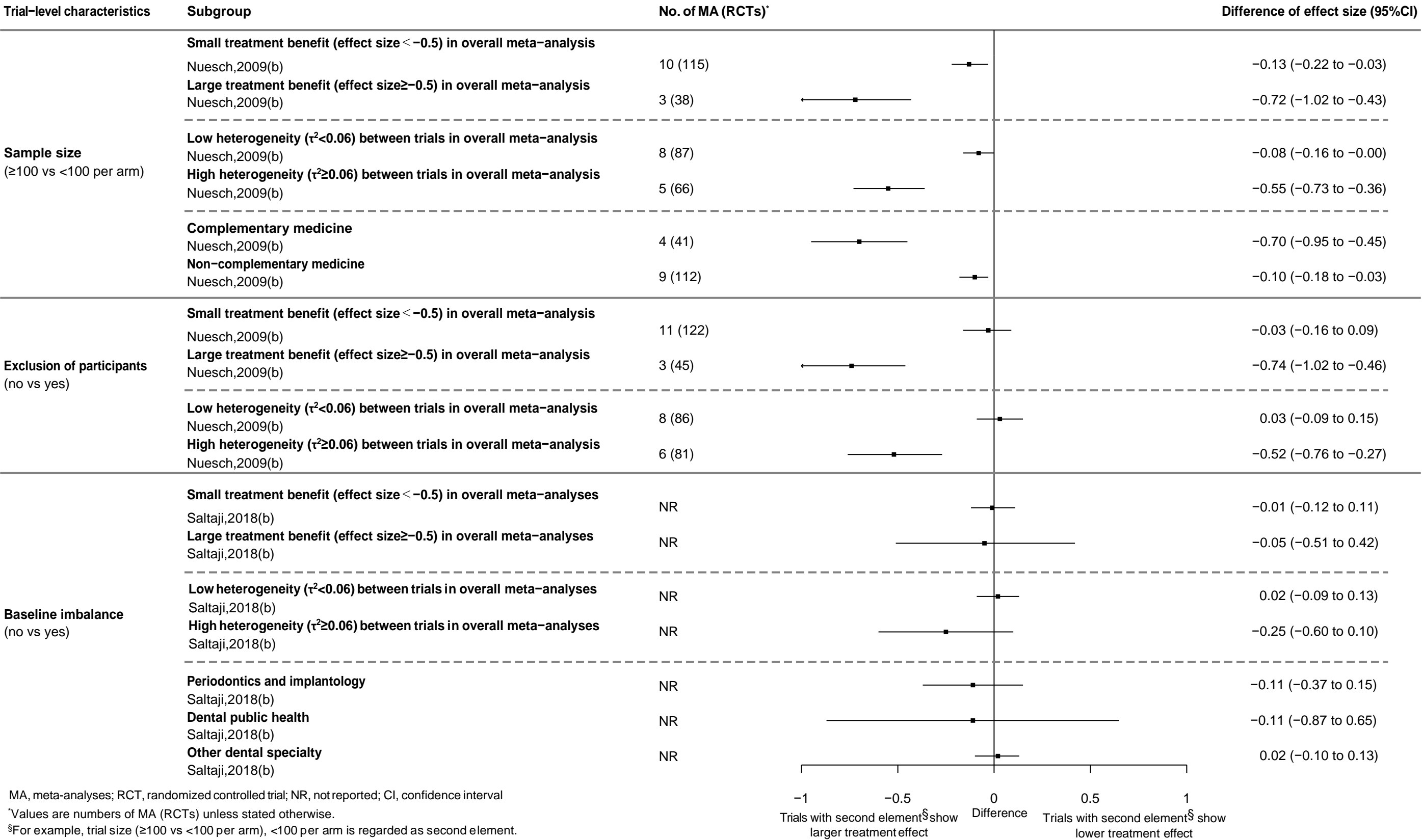

Supplement: Supplementary file 12 — Additional file 12: Appendix 12. Results of additional subgroup analyses. [file 12874_2022_1650_MOESM12_ESM.zip › Appendix 12-C-6.pdf]
